# Synthetic Polyploidisation Enhances *Fusarium graminearum* Tolerance in Wheat by Reshaping the Transcriptome and Strengthening the Microbiome

**DOI:** 10.1111/pbi.70295

**Published:** 2025-08-10

**Authors:** Xin He, Xiaolin Wang, Hao Li, Jiayong Shen, Mengchen Jiang, Xiaowei Zhang, Zeng Dong, Kai Sun, Fang Nie, Zhiwei Chen, Yun Zhou, Guoyong An, Ertao Wang

**Affiliations:** ^1^ State Key Laboratory of Crop Stress Adaptation and Improvement, College of Agriculture, School of Life Sciences Henan University Kaifeng China; ^2^ National Key Laboratory of Plant Molecular Genetics, Chinese Academy of Sciences Center for Excellence in Molecular Plant Sciences, Institute of Plant Physiology and Ecology, Shanghai Institutes for Biological Sciences Chinese Academy of Sciences Shanghai China; ^3^ South China Institute for Soybean Innovation Research, Guangdong Basic Research Center of Excellence for Precise Breeding of Future Crops, Guangdong Laboratory for Lingnan Modern Agriculture, College of Agriculture South China Agricultural University Guangzhou China; ^4^ College of Life Sciences Nanjing Normal University Nanjing China; ^5^ Biotechnology Research Institute Shanghai Academy of Agricultural Sciences Shanghai China

**Keywords:** dehydrin, disease tolerance, *Fusarium graminearum*, microbiome homeostasis, polyploidisation, wheat

## Abstract

Polyploidisation is a natural evolutionary mechanism that enhances plant stress tolerance and environmental adaptability; however, its impact on microbiome homeostasis remains poorly understood. In this study, we selected a nascent euploid synthetic hexaploid wheat line (HG116; 2*n* = 6*x* = 42, BBAADD) by selfing a triploid F1 hybrid of *Triticum turgidum* L. ssp. *durum* (Langdon, LDN; 2*n* = 4*x* = 28, BBAA) and *Aegilops tauschii* Coss. (SY41; 2*n* = 2*x* = 14, DD). We investigated the effects of synthetic polyploidisation on gene expression in roots, the root‐associated microbiome and tolerance to *Fusarium graminearum*. Transcriptomic analysis revealed that polyploidisation in HG116 predominantly upregulated genes, which were enriched in stress‐ and defence‐related pathways, particularly those involved in responses to pathogens and biotic stress. Microbiome profiling showed that HG116 recruited beneficial bacterial taxa and suppressed potential fungal pathogen growth in its rhizosphere and root endosphere compared to its parental lines. In *F. graminearum* inoculation experiments, HG116 demonstrated tolerance comparable to that of the *F. graminearum*‐resistant variety, in contrast to its susceptible parental varieties. Moreover, HG116 maintained microbial homeostasis by enriching Gram‐positive bacteria such as Actinobacteria and Firmicutes. *F. graminearum* inoculation also triggered extensive transcriptional reprogramming in HG116, including the upregulation of dehydrin, universal stress protein and defence‐related genes, which might collectively contribute to *F. graminearum* tolerance. These findings support the possibility that synthetic polyploidisation could enhance wheat's tolerance to *F. graminearum* by reshaping transcriptomic and microbial networks, offering valuable insights for developing more resilient wheat cultivars.

## 1 Introduction

Wheat ( *Triticum aestivum* ), the crop with the largest cultivation area of arable land globally, contributes approximately 20% of the world's dietary protein and energy, making it indispensable for global food security. However, wheat production faces significant challenges, including environmental stress and pathogen attack, which reduce yield and quality. As a devastating fungal pathogen, *F. graminearum* primarily infects staple cereal crops (wheat, barley, maize and rice), causing severe diseases that lead to substantial yield losses. Moreover, its production of mycotoxins, particularly deoxynivalenol (DON), represents a significant threat to food safety and human health (Pinto et al. 2022). While traditional approaches such as fertiliser and pesticide use have been employed to combat various diseases, these have limitations, including environmental concerns and health risks associated with excessive agrochemical use (Sharma and Singhvi 2017). Plants host diverse microbiomes on their surfaces, within internal tissues, and in the surrounding soil, collectively referred to as the plant microbiome. The plant microbiome has emerged as a promising alternative for sustainable disease management, offering potential solutions to enhance wheat tolerance/resistance against *F. graminearum*.

Recent advancements in high‐throughput sequencing technologies have revealed that plant‐associated microbiomes play critical roles in crop productivity and resilience (Müller et al. 2016; Verma and Suman 2018). In wheat, these microbial communities facilitate nutrient uptake, enhance plant growth and bolster stress tolerance/resistance through dynamic interactions and microbial diversity (Trivedi et al. 2020). For example, plant growth‐promoting rhizobacteria (PGPRs) mitigate abiotic stresses such as drought and salinity stress by altering endogenous phytohormone levels and gene expression (Barnawal et al. 2017), while psychrotrophic Pseudomonads enhance wheat productivity in cold environments (Mishra et al. 2011). Additionally, microbial taxa such as Proteobacteria, Actinobacteria, and Ascomycota, which dominate the wheat rhizosphere, contribute to nutrient uptake and pathogen defence (Bulgarelli et al. 2015; Gruet et al. 2022; Verma and Suman 2018). These results highlight the potential of microbiome‐based strategies for managing diseases.

Polyploidisation, or whole‐genome duplication, refers to the phenomenon in which organisms acquire three or more complete sets of chromosomes through whole‐genome replication (Lei and Bennett 1997; Song et al. 2012; Tossi et al. 2022). This natural evolutionary mechanism, which is common in the plant kingdom, equips plants with enhanced environmental adaptability, including stress tolerance/resistance (Tossi et al. 2022; Van de Peer et al. 2021; Wei et al. 2016). Genetic and epigenetic changes resulting from polyploidisation give rise to morphological and physiological changes that enhance plant adaptability, such as increased organ size and higher secondary metabolite contents (Nejadsadeghi et al. 2014; Tossi et al. 2022; Wang, Cao, et al. 2021). Polyploid plants can enhance stress tolerance/resistance by altering cell size and structure, increasing the content of osmotic regulators, enhancing the activity of antioxidant systems, regulating biomembrane systems, increasing gene expression, regulating epigenetic changes and so on (Hias et al. 2018; Ma et al. 2012; Tossi et al. 2022; Wang, Cao, et al. 2021; Wei et al. 2019, 2016). Specifically, polyploidisation amplifies disease tolerance by creating gene redundancy. This redundancy, evident in expanded gene families like nucleotide‐binding leucine‐rich repeat (NLR) receptors, provides functional backup copies that enable diversification of pathogen recognition mechanisms (Andersen et al. 2020; Hao et al. 2023). These clusters, maintained through tandem duplications and subgenome interactions, allow functional specialisation. Some NLRs detect pathogens via variable leucine‐rich repeats while others incorporate kinase domains for signalling diversification (Chakraborty et al. 2018). The redundancy also permits neo‐functionalisation through mechanisms like alternative splicing, where transcripts include/exclude integrated domains to modulate defence responses (Andersen et al. 2020; Chakraborty et al. 2018). The epigenetic buffering system (coordinating subgenome expression) enables wheat to balance diverse defence responses and developmental plasticity (Li, Zhang, et al. 2023).

Common bread wheat ( *Triticum aestivum* L.; 2*n* = 6*x* = 42, BBAADD) is a typical allohexaploid wheat originating from natural hybridisation between domesticated allotetraploid durum wheat ( *T. turgidum* ssp. *durum* (Desf.) MacKey; AABB, 2*n* = 4*x* = 28) and the wild diploid *Ae. tauschii* Coss. (2*n* = 2*x* = 14, DD), followed by genome duplication, making it a product of polyploidisation and long‐term domestication (Wang, Wang, Xie, et al. 2022). The two rounds of polyploidisation created an extremely complex genome in bread wheat, making this an excellent model for studying the structure, function and epigenetic changes of allopolyploid genomes (Li et al. 2018). The D subgenome, introduced during wheat's second polyploidisation, significantly contributes to stress resilience. Genomic analyses reveal that D‐subgenome‐specific genes from *Ae. tauschii* enhance pathogen recognition (e.g., NLR receptor clusters) and root exudate‐mediated microbiome remodelling (e.g., triterpenoid biosynthesis), synergistically fortifying wheat against biotic stresses (Gaurav et al. 2022; Wang et al. 2024). Domestication and polyploidisation are key drivers of wheat evolution that have not only increased wheat yield and adaptability but also influenced its rhizosphere microbiome to sustain belowground nutrient cycling and enhance plant growth (Yue et al. 2023). Different wheat genotypes resulting from domestication exhibit significant variations in rhizosphere microbiome diversity and community structure. For example, domesticated bread wheat shows lower bacterial diversity than its wild relative (Özkurt et al. 2020). Wild wheat harbours a more diverse rhizosphere bacterial community, dominated by Firmicutes and Actinobacteria, while domesticated wheat is enriched in Proteobacteria and Bacteroidetes (Hassani et al. 2020). Similarly, domesticated wheat varieties exhibit lower abundances of potential fungal pathogens, such as Sordariomycetes, but a greater prevalence of obligate symbiotic fungi like Glomeromycetes (Spor et al. 2020). Notably, polyploidisation‐induced changes in root architecture and exudate composition create selective pressure favouring beneficial microorganisms with biocontrol potential. Hexaploid wheat secretes specialised root exudates (e.g., benzoxazinoids and tricin flavonoids) that recruit antagonistic microbes like *Pseudomonas* spp. to suppress pathogens such as *F. graminearum*, while simultaneously priming systemic resistance via jasmonate signalling (Hu et al. 2018; Polturak et al. 2023; Zhalnina et al. 2018). These metabolites also reduce symbiotic dependencies, as modern wheat cultivars show lower reliance on arbuscular mycorrhizal fungi than wild relatives (Blaschkauer and Rachmilevitch 2023; Tkacz et al. 2020). However, hybrids retaining wild subgenomes (e.g., D from *Ae. tauschii*) exhibit enriched microbial functional diversity, including beneficial taxa like *Trichoderma* fungi and *Bacillus* bacteria (Tkacz et al. 2020). Collectively, these adaptations highlight how polyploidisation and domestication reconfigure plant‐microbiome interactions to balance pathogen tolerance with ecosystem stability—a process underexplored in subgenome‐specific contexts.

Microbiome dysbiosis, an imbalance between beneficial and pathogenic microbial populations, is a hallmark of plant diseases (Trivedi et al. 2020). Recent findings underscore the critical roles of rhizosphere microbiomes in shaping disease resistance in wheat. For example, in a study comparing four wheat cultivars with differential Fusarium head blight (FHB) resistance, resistant varieties demonstrated significantly higher rhizosphere bacterial diversity yet lower fungal diversity compared to susceptible counterparts. Notably, fungal abundance was paradoxically elevated in resistant cultivars, suggesting a compositional shift toward functionally relevant microbiota that may contribute to disease suppression (Li, Tang, et al. 2023). Similarly, wheat varieties resistant to dwarf bunt (*Tilletia controversa*) displayed higher fungal diversity and abundance than susceptible varieties whether or not they were inoculated with *Tilletia controversa*. Resistant varieties were enriched in beneficial taxa such as Sordariomycetes, Mortierellomycetes, Chryseobacterium and Massilia, while susceptible varieties were dominated by potentially pathogenic taxa, including Dothideomycetes, *Pseudomonas* and Nocardioides (Xu et al. 2021). Moreover, beneficial rhizosphere bacteria such as *Pseudomonas* and *Bacillus* contribute to disease suppression through mechanisms including antibiotic production, induced systemic resistance and pathogen competition (Karlsson et al. 2021; Legrand et al. 2017). However, while protective microbiome underpins disease resistance, the unique role of polyploidisation in restructuring the wheat microbiome for disease tolerance enhancement lacks mechanistic insight.

In this study, we used a three‐step approach to address this issue. We (1) took an euploid synthetic hexaploid wheat (SHW; HG116; 2*n* = 6*x* = 42, BBAADD) line at S1 generation as a model, which was generated by selfing the triploid F1 hybrid of *T. turgidum* L. ssp. *durum* (Langdon, LDN; 2*n* = 4*x* = 28, BBAA) and *Ae. tauschii* Coss. (SY41; 2*n* = 2*x* = 14, DD) (Li et al. 2016); (2) investigated the effects of synthetic hexaploidisation on gene expression in wheat roots and root‐associated microbiomes; and (3) combined multi‐omics with experimental approaches to investigate how SHW regulates gene expression and rhizosphere microbiome homeostasis to defend *F. graminearum* compared to diploid, tetraploid and hexaploid wheat. Our findings advance the understanding of wheat polyploidisation and highlight the potential of leveraging microbiome‐mediated mechanisms for sustainable disease management in wheat production systems.

## 2 Results

### 2.1 Development, Karyotyping and Traits of Synthetic Hexaploid Wheat Line HG116

We developed the SHW line HG116 by crossing the tetraploid *T. turgidum* L. ssp. *durum* accession Langdon (LDN; 2*n* = 4*x* = 28, BBAA) as the female parent with the diploid *Ae. tauschii* Coss. accession SY41 (2*n* = 2*x* = 14, DD) as the male parent. We pollinated Langdon (LDN) with SY41 pollen to produce triploid F1 hybrids; no embryo rescue or hormone treatment was applied to the F1 seeds. The synthesised allohexaploid F1 plants were self‐pollinated to produce S0 generation seeds by the fusion of unreduced gametes, leading to the formation of SHW (Li et al. 2016). Subsequently, selfing of the S0 generation yielded S1 generation HG116 plants, with a chromosome number of 2*n* = 6*x* = 42, BBAADD.

**FIGURE 1:**
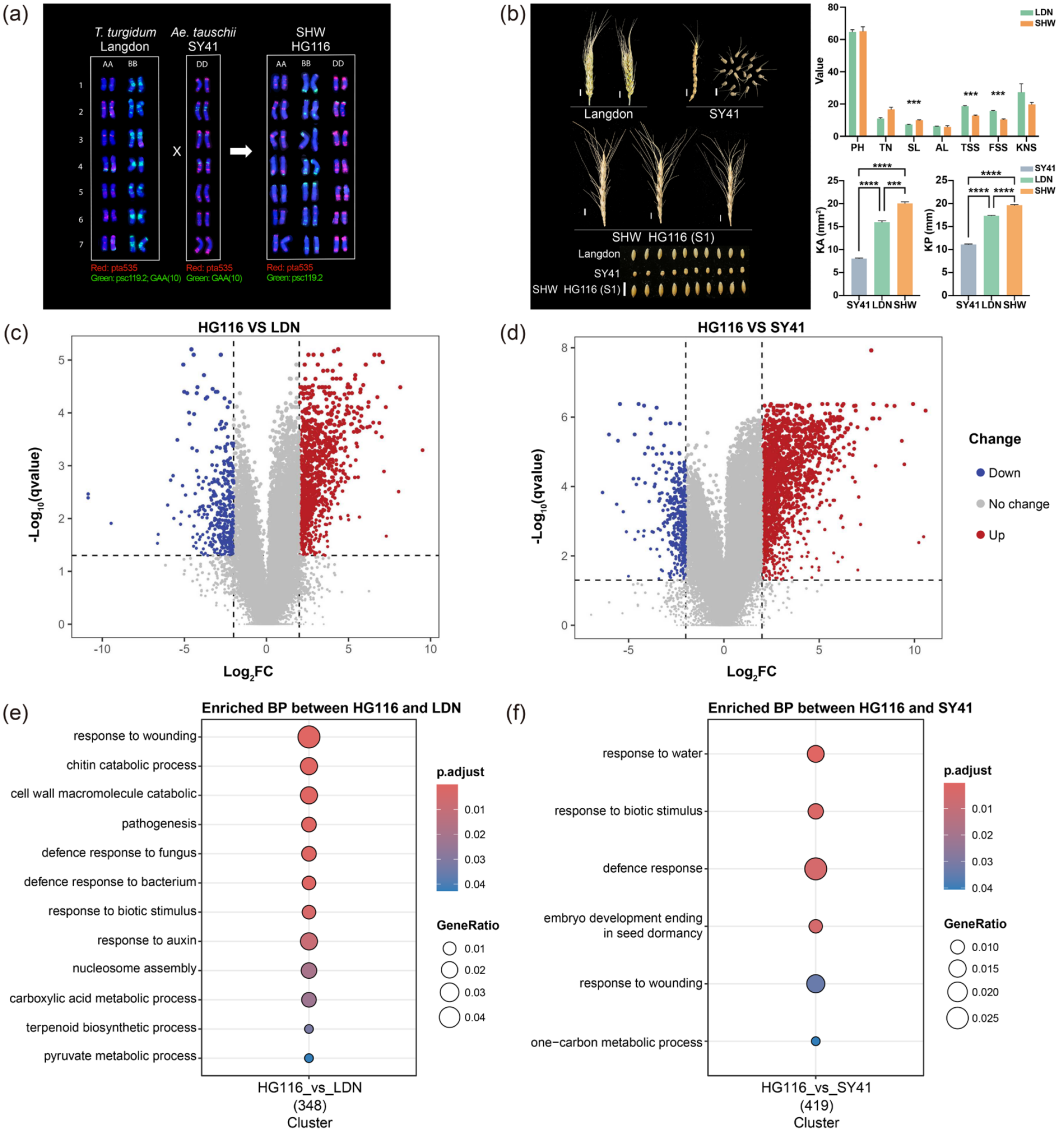
Analysis of karyotyping, traits and root transcriptome differences among different ploidy‐level wheat accessions. (a) FISH (Fluorescence in situ hybridisation) karyotypes of HG116 with its parents Langdon (LDN) and SY41. Chromosome groups A, B and D were distinguished by the different coloured signals. Red represents oligo‐pTa535 signals, whereas green represents oligo‐pSc119.2 and oligo‐(GAA)10 signals. (b) Phenotypic characterisations of spikes and kernels, as well as statistics of agronomic traits, for HG116 and its parents LDN and SY41. Bar = 1 cm. AL, Awn length (cm); FSS, Fertile spikelets (number); KA, Kernel area; KNS, Kernel number per spike (number); KP, Kernel perimeter; PH, plant height (cm); SL, spike length (cm); TN, Tiller number; TSS, total spikelets (number). Data are means ± SE. ****p < 0.0001. (c, d) Differential gene expression analysis showing up and downregulated genes between HG116 and LDN (c) and SY41 (d). The upregulated genes are indicated in red, whereas the downregulated genes are indicated in blue. The black horizontal dashed line corresponds to the *q* value of 0.05. Left and right black vertical dashed lines correspond to log_2_FC of −2 and 2 (FC, fold change), respectively. (e, f) Gene ontology (GO) annotations of differentially expressed genes (DEGs with |log_2_FC| > 2 and *q* < 0.05) between HG116 and LDN (e) and SY41 (f). The GeneRatio and BH‐adjusted enrichment *p* value of the biological processes are indicated using the size and colour of the bubble points, respectively. The colour of dots indicates high or low enrichment for a specific GO category. The size of dots display the overlap between the input gene lists and the collection of gene sets.

To confirm that HG116 was indeed a hexaploid and to assess its chromosome number and potential structural variations, we performed karyotyping on LDN (BBAA), SY41 (DD) and HG116. With the aid of a probe set of oligo‐pTa535, oligo‐pSc119.2 and oligo‐(GAA)_10_, the A, B, D chromosomes of both HG116 and its parent lines could be clearly identified and tracked. LDN (BBAA), SY41 (DD) and HG116 were confirmed to possess 28, 14 and 42 chromosomes, respectively, indicating that all lines were complete euploids without chromosomal structural variations. Furthermore, these results confirm that HG116 completely inherited its genetic information from both LDN and SY41 (Figure 1a).

The parental line SY41 exhibited moniliform spikes with hard glumes that caused spike shattering at maturity. By contrast, neither LDN nor HG116 showed spike shattering. Although SY41 had hard, non‐threshable glumes, LDN exhibited easy threshing, while their derivative, HG116, was not easily threshable. HG116 had significantly longer spikes, lower total spikelets and lower fertile spikelet number per spike than LDN, but no significant differences in plant height, tiller number or awn length. HG116 also carried significantly larger kernel area and perimeter compared to both LDN and SY41 (Figure 1b). These findings indicate that HG116 possesses a relatively stable chromosomal constitution and exhibits superior spike length and kernel size traits compared to its parents.

### 2.2 Effects of Synthetic Polyploidisation on the Wheat Root Transcriptome

To investigate whether polyploidisation affects the wheat root transcriptome, we performed RNA sequencing (RNA‐seq) of the roots of three wheat accessions with different ploidy levels: HG116, LDN and SY41. Since LDN contained the AABB genomes, while SY41 contained only DD genomes, we divided the transcriptome data into two groups (AABB and DD) for comparative analysis.

We identified differentially expressed genes (DEGs) based on |log_2_(fold change)| > 2 and *q* < 0.05. In HG116 roots, 1034 DEGs were upregulated and 382 were downregulated compared to LDN (Figure 1c; Table S1a), whereas 1703 DEGs were upregulated and 364 were downregulated compared to SY41 (Figure 1d; Table S1b), suggesting that synthetic polyploidisation predominantly led to gene upregulation in wheat roots.

Notably, more DEGs were present in root tissue of SHW line HG116 compared to its diploid parent SY41 (DD_HG116_vs_SY41: 2067 DEGs; Figure 1d; Table S1b) and its allotetraploid parent LDN (AABB_HG116_vs_LDN: 1416 DEGs; Figure 1c; Table S1a), even though collectively, the AA and BB subgenomes are twice as large as the DD subgenome (Appels et al. 2018). These results suggest that the expression of genes in the D subgenome was more strongly affected by the synthetic polyploidisation than that of genes in the other subgenomes.

GO enrichment analysis of the DEGs between HG116 and the two parents (LDN and SY41) showed that DEGs in both the AABB and D subgenomes shared enriched pathways related to stress responses, including response to biotic stimulus and response to wounding (Figure 1e,f). DEGs in the AABB subgenomes also showed significant enrichment of other stress and defence responses, involving pathways such as defence response to fungus, defence response to bacterium, response to auxin and nucleosome assembly (Figure 1e). DEGs of the D subgenome between HG116 and SY41 were also enriched in the GO terms response to water and defence response (Figure 1f). These results suggest that synthetic polyploidisation might have endowed HG116 with stronger disease and stress tolerance compared to its parents.

Moreover, DEGs in the AABB subgenomes exhibited broader and more diverse biological processes, including chitin catabolic process, terpenoid biosynthetic process, pyruvate metabolic process, carboxylic acid metabolic process and cell wall macromolecule catabolic process (Figure 1e), whereas DEGs in the D subgenome showed unique enrichment of the GO terms embryo development ending in seed dormancy and one‐carbon metabolic process (Figure 1f). This suggests that the AABB subgenomes might play crucial roles in maintaining the basic metabolic activities and structural integrity of wheat, whereas the D subgenome might be highly important for the survival, reproduction and adaptability of plants. Collectively, these results highlight the complexity of polyploidisation‐induced changes in gene expression in wheat roots and suggest that the functional differences between these subgenomes might contribute to the variation in stress tolerance and adaptability among different wheat varieties.

### 2.3 Effects of Synthetic Polyploidisation on the Wheat Root‐Associated Microbiome

**FIGURE 2:**
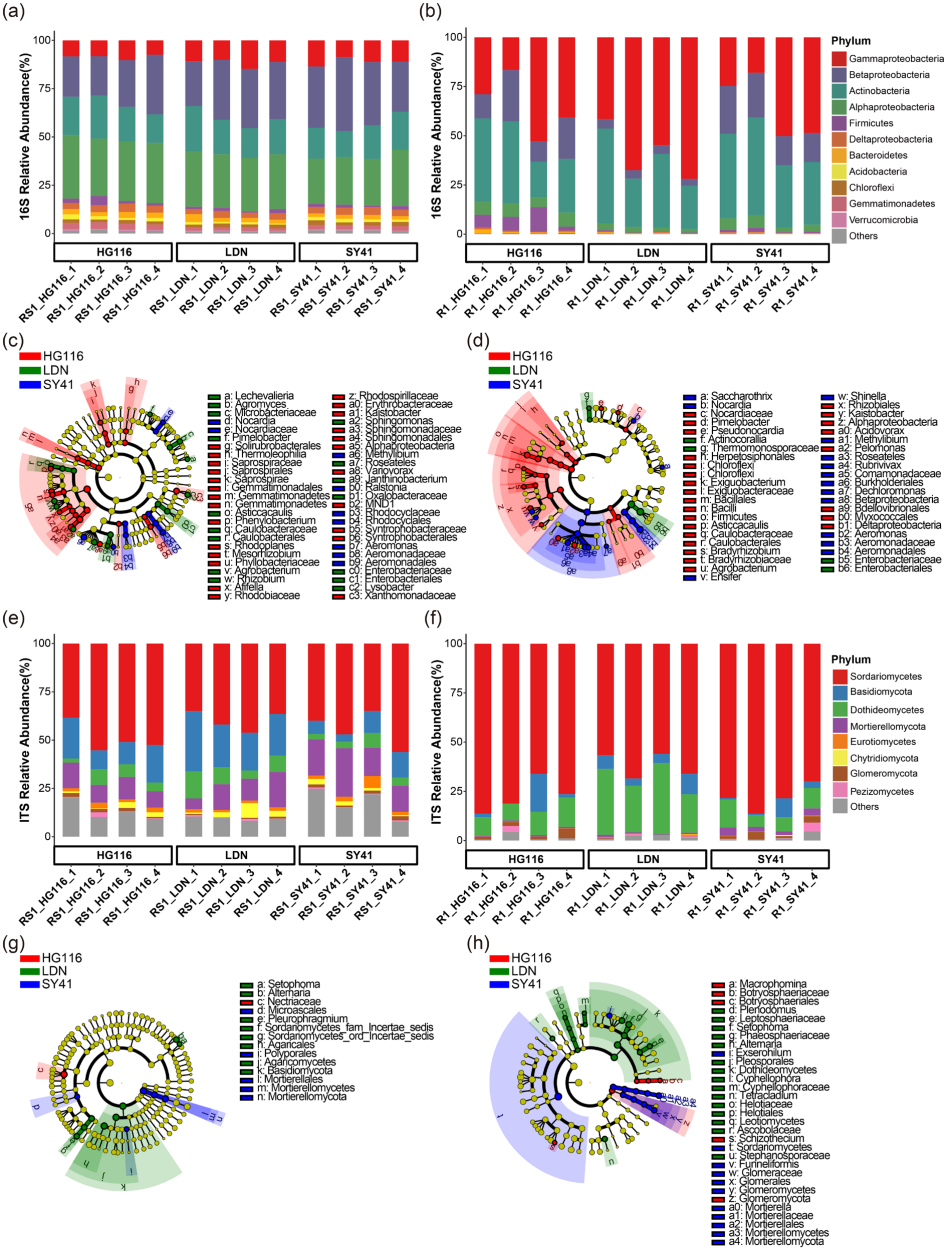
Root‐associated bacterial and fungal compositions and biomarkers among different ploidy‐level wheat accessions. (a, b) Comparing phylum‐level distribution of the rhizosphere (a) and root endophytic (b) bacterial microbiota among HG116, LDN and SY41 using 16S rRNA genes. (c, d) LDA effect size taxonomic cladogram comparing rhizosphere (c) and root endophytic (d) bacterial samples categorised by HG116, LDN and SY41. (e, f) Comparing the phylum‐level distribution of the rhizosphere (e) and root endophytic (f) fungal microbiota among HG116, LDN and SY41 using ITS rRNA genes. (g, h) LDA effect size taxonomic cladogram comparing rhizosphere (g) and root endophytic (h) fungal samples categorised by HG116, LDN and SY41. For (c), (d), (g) and (h), significantly discriminant taxon nodes are coloured and branch areas are shaded according to the highest‐ranked variety for that taxon. If the taxon is not significantly differentially represented between sample groups, the corresponding node is coloured yellow. Significantly discriminant taxa from bacterial phylum to genus level are labelled on the right.

We evaluated the effects of synthetic polyploidisation on the assembly of the bacterial and fungal microbiota in the rhizosphere and root endosphere. We detected significant differences in the relative abundances of bacterial and fungal phyla among the three wheat varieties with different ploidy levels (Kruskal–Wallis test, *p* < 0.05; Figure 2). At the bacterial phylum level, Betaproteobacteria exhibited the highest relative abundance in the rhizosphere of diploid SY41, followed by tetraploid LDN and then synthetic hexaploid HG116 (Figure 2a; Table S2a). Additionally, Gammaproteobacteria exhibited the lowest relative abundance in both the rhizosphere and root endophytic microbiota of HG116 compared to LDN and SY41 (Figure 2a,b; Table S2a,b). By contrast, Alphaproteobacteria, Deltaproteobacteria, Firmicutes, Bacteroidetes and Acidobacteria showed the highest relative abundances in both the rhizosphere and root endophytic bacterial microbiota of HG116 compared to its parental accessions LDN and SY41 (Figure 2a,b; Table S2a,b).

These findings were validated at the bacterial family level (Figure S1a,b; Table S2e,f) and further supported by LEfSe analysis to identify significant biomarkers that distinguished between HG116 and its parents LDN and SY41 (LDA score (log_10_) > 2.0; *p* < 0.05; Figure 2c,d). We identified 25, 16 and 9 bacterial biomarkers in the rhizosphere bacterial microbiomes of HG116, LDN and SY41, respectively. Many of the biomarkers associated with HG116 (e.g., Phyllobacteriaceae, Rhodobiaceae, *Variovorax*, *Afifella*) support plant health (e.g., promoting nutrient uptake, stress and disease tolerance) and ecosystem function. LDN and SY41 were associated with more Gammaproteobacteria families, such as Enterobacteriaceae and Aeromonadaceae, which might not provide the same benefits as the biomarkers in HG116 (Figure 2c). We identified 47 bacterial biomarkers in the root endophytic bacterial microbiome. HG116 had the greatest number of biomarkers, including Exiguobacteraceae, Nocardiaceae, Caulobacteraceae and Bradyrhizobiaceae, which are associated with nitrogen fixation, pathogen suppression and plant growth promotion. LDN harboured only four biomarkers (e.g., Enterobacteriaceae, Thermomonosporaceae), whereas SY41 had 14 (e.g., Comamonadaceae, Aeromonadaceae) (Figure 2d). These results suggest that HG116 has a more diverse and beneficial endophytic bacterial community than its parents.

At the fungal phylum level, Sordariomycetes exhibited higher relative abundance in the rhizosphere of HG116 compared to LDN and SY41 (Figure 2e; Table S2c) and in the root endosphere of HG116 compared to LDN (Figure 2f; Table S2d). At the fungal family level, Mortierellaceae showed the highest relative abundance in both the rhizosphere and root endophytic fungal microbiota of SY41 compared to HG116 and LDN (Figure S1c,d; Table S2g,h). However, unlike for the bacterial microbiota, the relative abundances of most fungal families did not exhibit a clear pattern in line with different ploidy levels. LEfSe identified 14 and 31 fungal biomarkers in the rhizosphere and root endophytic fungal microbiomes of the three wheat accessions, respectively (Figure 2g,h). Specifically, we identified five biomarkers in the root endophytic fungal microbiome of HG116, including arbuscular mycorrhizal fungi (Glomeromycota) and other beneficial or neutral fungi such as Botryosphaeriaceae, Botryosphaeriales, *Macrophomina* and *Schizothecium*. By contrast, LDN harboured 15 biomarkers, including potential pathogens such as *Alternaria* and *Plenodomus*. SY41 contained 11 biomarkers, including potential pathogens such as *Exserohilum*, arbuscular mycorrhizal fungi (Glomeromycetes‐Glomerales‐Glomeraceae‐*Funneliformis*), and saprophytic clades (Mortierellomycota‐Mortierellomycetes‐Mortierellales‐Mortierellaceae‐*Mortierella*).

These results suggest that synthetic polyploidisation had a more pronounced effect on the bacterial than on the fungal community composition of the wheat rhizosphere and root endophytic microbiota. Synthetic polyploidisation in wheat appeared to facilitate the recruitment of beneficial bacteria while repelling potentially pathogenic fungi in the roots. Therefore, synthetic polyploidisation endowed wheat with root‐associated microbial communities with a high degree of adaptability and flexibility, enabling them to adjust microbial abundances in response to environmental or genetic changes.

### 2.4 Disease Tolerance in Synthetic Hexaploid Wheat is Associated With High Dehydrin Gene Expression

**FIGURE 3:**
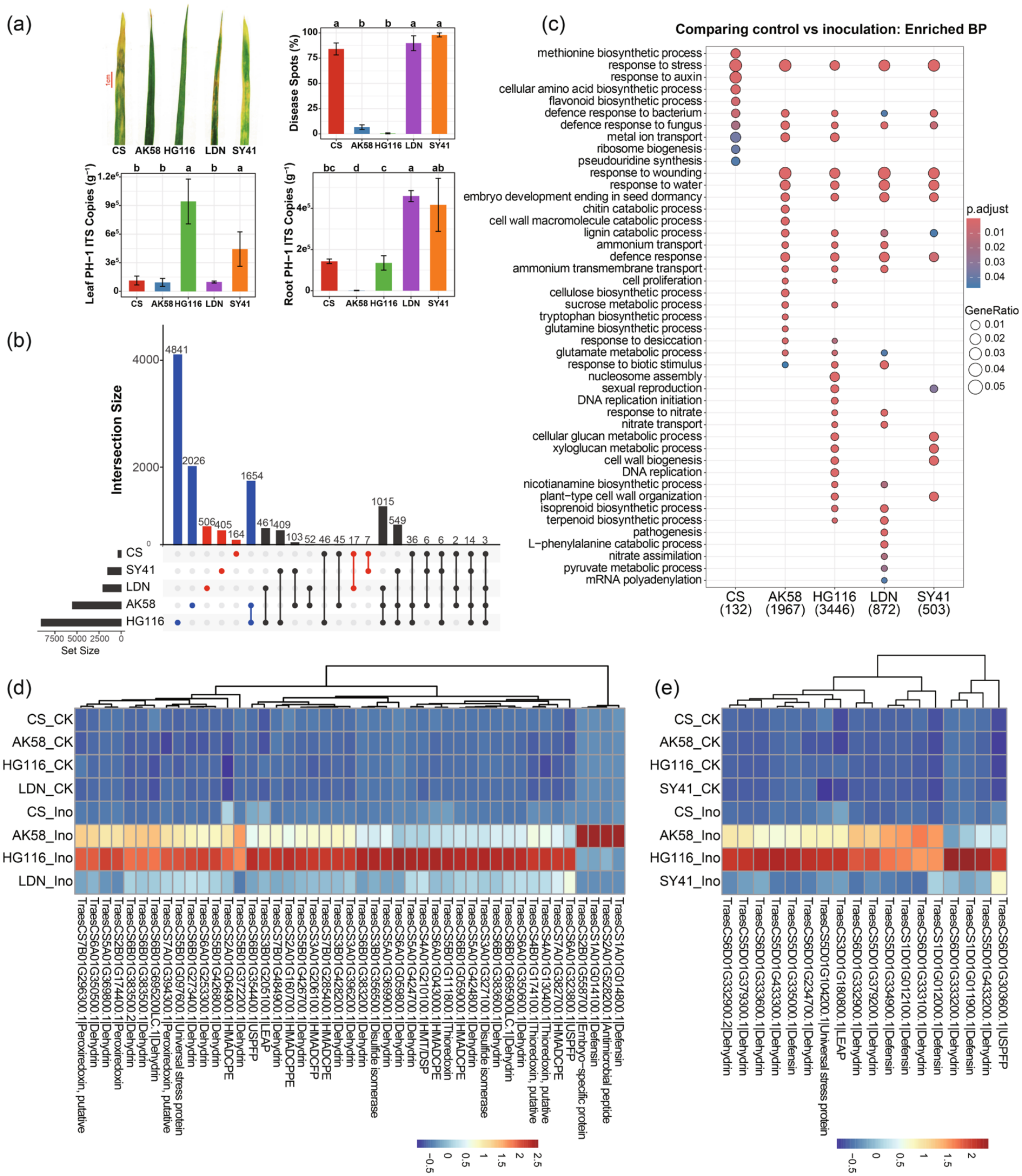
Comparative analysis of the root transcriptome among wheat varieties with different disease resistances following *Fusarium graminearum* infection. (a) Comparisons of leaf phenotypes and relative disease spots area, and PH‐1 ITS copies in leaf and root between tolerant/resistant wheats and susceptible wheats following *F. graminearum* strain PH‐1 inoculation. CK represented control, Ino represented inoculation. Post hoc test was indicated by letters at the top; sample groups with the same letter are indistinguishable at 95% confidence. *n* = 4 biological replicates. (b) UpSet plots of differentially expressed genes (DEGs with |log_2_FC| > 2 and *q* < 0.05) between wheat varieties with different disease resistances and their respective controls. (c) Gene ontology (GO) annotations of DEGs (with |log_2_FC| > 2 and *q* < 0.05) between wheat varieties with different disease resistances and their respective controls. The GeneRatio and BH‐adjusted enrichment *p* value of the biological processes were indicated using the size and colour of the bubble points, respectively. The colour of dots indicated high or low enrichment for a specific GO category. The size of dots displays the overlap between the input gene lists with the collection of gene sets. (d, e) Heatmap bar plot of the expression levels of hub DEGs in AABB‐MEturquoise module (d), DD‐MEblue module (e) (correlated with HG116) and AABB‐MEmagenta module (d) (correlated with AK58) across wheat varieties with different disease resistances, being enriched in the following biological processes: response to water, response to stress, metal ion transport, cell redox homeostasis, defence response and defence response to fungus (Figure S5a–c). The expression levels of hub DEGs had been standardised using *Z* score. The colour indicates high or low expression levels for hub DEGs. CK represents control, Ino represents inoculation. HMADCFP, heavy metal‐associated domain‐containing family protein; HMADCPE, heavy metal‐associated domain containing protein, expressed; HMADCPPE, heavy metal‐associated domain‐containing protein, putative, expressed; HMT/DSP, heavy metal transport/detoxification superfamily protein; LEAP, late embryogenesis abundant protein; USPFP, universal stress protein family protein.

Both the root transcriptome and root‐associated microbiomes underscore the potential role of polyploidisation in enhancing disease tolerance in wheat. To test this hypothesis, we inoculated the SHW line HG116 and its parents LDN and SY41, along with the dwarf resistant variety AK58 (2*n* = 6*x* = 42, BBAADD) and the susceptible variety CS (2*n* = 6*x* = 42, BBAADD), with *F. graminearum* strain PH‐1 to assess whether polyploidisation influences pathogen tolerance in wheat. HG116 exhibited tolerance to *F. graminearum* strain PH‐1 comparable to that of the dwarf resistant variety AK58. By contrast, the susceptible variety CS, as well as the diploid parent SY41 and allotetraploid parent LDN, showed no resistance to the pathogen (Figure 3a). The relative disease spot areas on the leaves of HG116 and AK58 were significantly smaller than those of CS, LDN and SY41. However, PH‐1 absolute abundance in root samples did not differ significantly between HG116 and the disease‐susceptible variety CS. Notably, HG116 showed highest PH‐1 absolute abundance in leaf (Figure 3a). These results suggest that HG116 maintains its health not by suppressing pathogen proliferation but by tolerating the presence of the pathogen. These findings confirm the notion that synthetic polyploidisation induces *F. graminearum* tolerance in wheat.

To uncover the genetic basis of this tolerance, we analysed the effect of *F. graminearum* infection on the wheat root transcriptome. Following PH‐1 inoculation, HG116 and AK58 displayed more DEGs than the susceptible varieties, with 1654 DEGs shared between HG116 and AK58 (Figure 3b; |log_2_(fold change)| > 2 and *q* < 0.05). GO enrichment analysis revealed that DEGs across all wheat varieties with varying fitness when challenged with PH‐1 shared enriched pathways related to stress and defence responses, including response to stress, defence response to bacterium and defence response to fungus (Figure 3c). Additionally, DEGs across AK58, HG116, LDN and SY41 were significantly enriched in response to wounding, response to water and defence response (Figure 3c). These results suggest that potential mechanisms by which SHW may achieve *F. graminearum* tolerance involve extensive transcriptional reshaping, though functional validation is required to establish causal relationships. We performed hierarchical clustering analysis of all DEGs enriched in categories such as response to stress, response to water, defence response, defence response to fungus, defence response to bacterium and response to wounding (Figures S2 and S3). Numerous dehydrin and defensin genes were overexpressed in HG116 and AK58 roots following PH‐1 inoculation (Figure S2). Additionally, some genes encoding the pathogenesis‐related protein PR‐4 were upregulated in CS AK58, HG116 and SY41, while several genes encoding chymotrypsin inhibitors were downregulated in all wheat varieties following PH‐1 inoculation (Figure S3). Previous studies have demonstrated that dehydrins can maintain cellular homeostasis by their multiple mechanisms and modulate defence signalling pathways to upregulate PR genes (Brini et al. 2011; Hanin et al. 2011), implying that the significant upregulation of dehydrin genes may have a potential contribution to *F. graminearum* tolerance of HG116, possibly via coordinated regulation of the maintenance of normal plant cell functions, defence‐related signal transduction and PR gene induction.

Weighted gene co‐expression network analysis (WGCNA) using transcriptome data from PH‐1‐inoculated and uninoculated roots identified 11 unique gene co‐expression modules in each subgenome (Figure S4a,b). Correlation analysis revealed that the MEturquoise module (*r* = 0.73, *p* = 2 × 10^−6^) in the AABB subgenomes and the MEblue module (*r* = 0.75, *p* = 8 × 10^−7^) in the DD subgenomes were most strongly associated with the tolerant line HG116 (Figure S4a,b). Similarly, the MEmagenta module (*r* = 0.94, *p* = 9 × 10^−16^) in the AABB subgenomes was highly correlated with the resistant variety AK58 (Figure S4a). Module membership (MM) and gene significance (GS) analyses of HG116 and AK58 further supported the significant correlations between these modules and tolerance/resistance traits (Figure S4c–e). We identified hub genes within these key modules using stringent criteria (|GS| > 0.5 and |MM| > 0.9) (Figure S4c–e; Table S3). This dual‐threshold strategy prioritised genes serving as central nodes within their modules while maintaining a biologically relevant connection to the tolerance phenotype, following standard WGCNA practice. Hub genes in the AABB MEturquoise and DD MEblue modules associated with HG116 were significantly enriched in the GO terms response to water and response to stress. Hub genes in the AABB MEturquoise module were enriched in metal ion transport and cellular redox homeostasis, whereas those in the DD MEblue module were enriched in defence response (Figure S5a,b). Hub genes in the AABB MEmagenta module associated with AK58 were enriched in defence response to fungus and defence response pathways (Figure S5c). Hub genes in these modules were enriched in KEGG pathways closely related to plant defence and immune responses, including the MAPK signalling pathway, plant hormone signal transduction, protein processing in the endoplasmic reticulum, inositol phosphate metabolism and phosphatidylinositol signalling system (Figure S5d–f).

Finally, we compared the expression levels of hub DEGs across the six key biological processes—response to water, response to stress, metal ion transport, cellular redox homeostasis, defence response to fungus and defence response—among wheat varieties with varying fitness when challenged with PH‐1 (Figure 3d,e), finding that disease resistance in AK58 might be associated with defensin and antimicrobial peptide genes. Furthermore, after PH‐1 inoculation, many dehydrin genes, some defensin genes and genes encoding universal stress protein and heavy metal‐associated domain‐containing protein were significantly upregulated in HG116 compared to its susceptible parents SY41 and LDN. These findings further support our notion that PH‐1 inoculation induced the massive production of dehydrins in SHW, thereby enhancing its tolerance to *F. graminearum*. The definitive conclusions about their functional contributions to *F. graminearum* tolerance await further genetic validation.

### 2.5 *Fusarium graminearum* Infection Decreases Diversity but Increases Abundance of Bacteria and Fungi in the Wheat Rhizosphere

To determine whether *F. graminearum* infection affected the assembly and ecological functions of the root‐associated microbiome in SHW HG116 compared to its diploid parent SY41 and allotetraploid parent LDN, we collected samples inoculated with PH‐1 under identical conditions and analysed them in parallel with the uninoculated controls.

**FIGURE 4:**
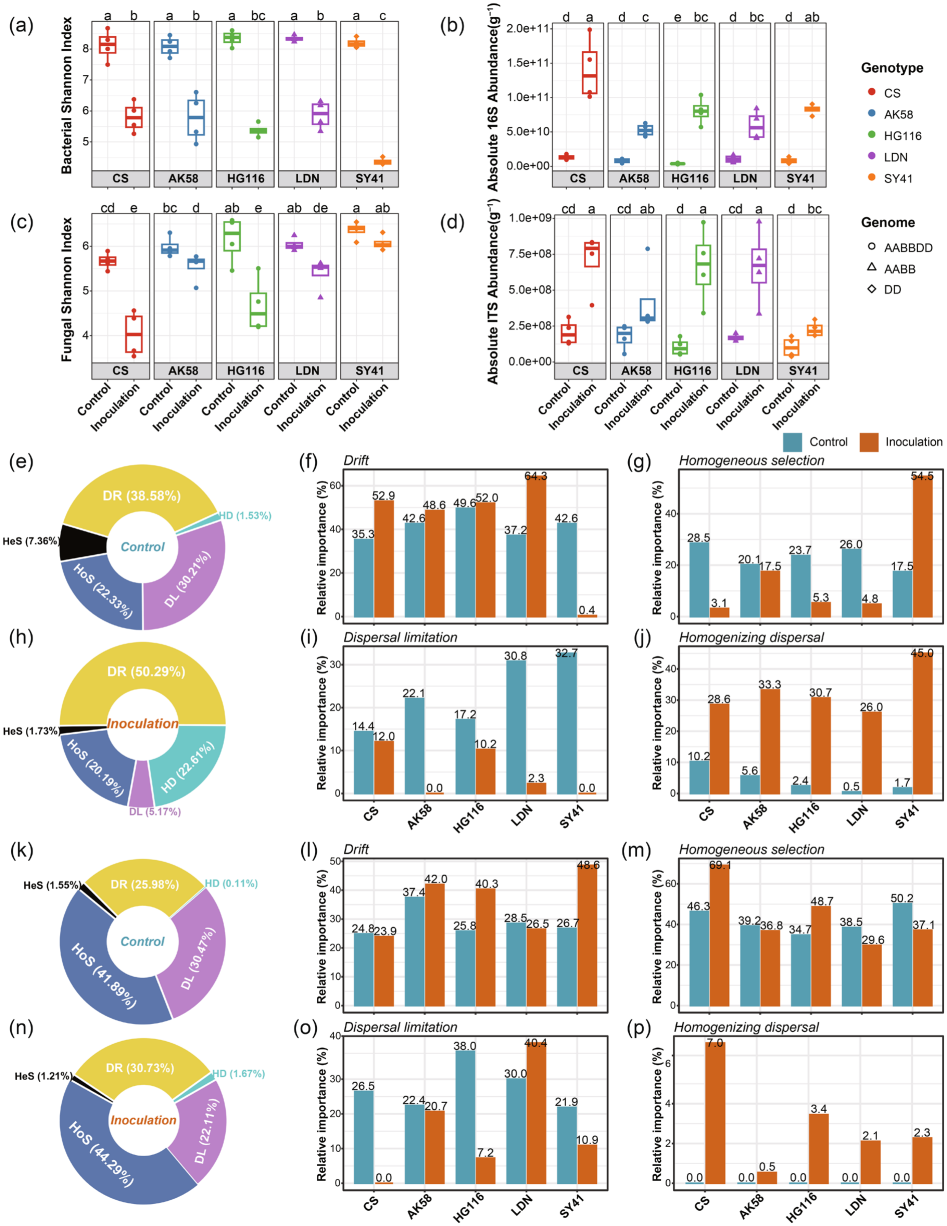
*Fusarium graminearum* infection altered the assembly of wheat rhizosphere‐associated microbiomes. (a–d) Comparison of the bacterial Shannon index (a) and absolute abundance (b) and the fungal Shannon index (c) and absolute abundance (d) between wheat varieties with different disease resistances and their respective controls. (e–p) The relative importance of different ecological processes in rhizosphere bacterial (e–j) and fungal (k–p) community assembly was estimated by iCAMP under control (e, k) and under inoculation (h, n). Specifically, the relative importance of Drift (f, l), Homogeneous selection (g, m), Dispersal limitation (i, o) and Homogenising dispersal (j, p) in rhizosphere (bacterial, fungal) community assembly of each wheat variety under control (aqua bar) and inoculation (orange bar). From (a) to (d), the colour and shape of each point represented the wheat variety (genotype) and genome, respectively. The post hoc test was indicated by letters at the top; sample groups with the same letter are indistinguishable at 95% confidence. *n* = 4 biological replicates. For (f), (g), (i), (j), (l), (m), (o) and (p), the digits on the top of each bar were the relative importance of each ecological process in governing the turnovers in rhizosphere bacterial and fungal community assembly of each wheat variety.

We first examined differences in the diversity of bacterial and fungal communities between control and inoculated samples. Alpha diversity metrics (Shannon's diversity and Faith's PD) revealed that PH‐1 infection significantly reduced the alpha diversity of rhizosphere bacterial and fungal microbiota in all wheat varieties (Kruskal–Wallis test, false discovery rate (FDR)‐adjusted *p* value (*q*) < 0.05; Figure 4a,c; Figure S6a,c). Faith's PD index for the root endophytic bacterial microbiota significantly declined following PH‐1 infection (Figure S6b), although the Shannon index did not decrease (Figure S7a). Similarly, PH‐1 infection did not significantly influence the Shannon index of root endophytic fungal microbiota (Figure S7c). By contrast, Faith's PD index of root endophytic fungal microbiota increased after PH‐1 infection in all varieties except LDN (Figure S6d). These findings suggest that the effects of *F. graminearum* infection on alpha diversity were stronger in the rhizosphere microbiota than in the root endophytic microbiota, with significant decreases in alpha diversity observed across the rhizosphere microbiota.

Quantitative microbiome profiling (QMP) revealed that the absolute abundances of rhizosphere bacterial and fungal microbiota significantly increased in all wheat varieties following PH‐1 infection (Figure 4b,d). By contrast, the absolute abundances of root endophytic fungal microbiota significantly decreased in all wheat varieties following PH‐1 infection (Figure S7d), while those of root endophytic bacterial microbiota significantly decreased in all varieties except HG116 and SY41 (Figure S7b).

Beta diversity analysis using PCoA based on Bray‐Curtis distance metrics showed that PH‐1 infection significantly affected the β‐diversity of bacterial and fungal microbiota in both the rhizosphere and root endophytic (rhizosphere bacteria: ADONIS2 *R* ^2^ = 0.49713, *p* = 1 × 10^−4^; ANOSIM *R* ^2^ = 0.9997, *p* = 1 × 10^−4^; root endophytic bacteria: ADONIS2 *R* ^2^ = 0.25448, *p* = 1 × 10^−4^; ANOSIM *R* = 0.699, *p* = 1 × 10^−4^; rhizosphere fungi: ADONIS2 *R* ^2^ = 0.19895, *p* = 1 × 10^−4^; ANOSIM *R* = 0.746, *p* = 1 × 10^−4^; root endophytic fungi: ADONIS2 *R* ^2^ = 0.26417, *p* = 1 × 10^−4^; ANOSIM *R* = 0.8487, *p* = 1 × 10^−4^; Figure S6e–h). Analysis based on weighted UniFrac distances revealed a similar trend (Figure S6i–l), indicating that PH‐1 infection is a key driving factor for the β‐diversity of rhizosphere and root endophytic bacterial and fungal microbiota.

To explore the effects of PH‐1 infection on microbial community assembly, we performed iCAMP (infer community assembly mechanisms by phylogenetic bin‐based null model analysis). For the rhizosphere bacterial communities, PH‐1 infection increased the relative importance of drift (DR; from 38.58% to 50.29%) while decreasing homogeneous selection (HoS; from 22.33% to 20.19%) and dispersal limitation (DL; from 30.21% to 5.17%) (Figure 4e–i). This trend was more pronounced in the root endophytic bacterial microbiota, where DR increased from 16.70% to 90.44%, HoS decreased from 26.37% to 3.91%, and DL decreased from 53.77% to 1.72% (Figure S7e–i). Additionally, PH‐1 infection had a stronger effect on the relative importance of homogenising dispersal (HD) in rhizosphere bacterial community assembly, which increased from 1.53% to 22.61% (Figure 4e,h,j). However, changes in the relative importance of HD in root endophytic bacterial communities varied among wheat varieties (Figure S7j).

In the fungal community, PH‐1 infection increased the relative importance of DR and HoS while decreasing DL in both the rhizosphere and root endophytic communities (Figure 4k,n; Figure S7k,n), but this trend varied across wheat varieties (Figure 4l,m; Figure S7l,m). DL consistently decreased in root endophytic fungal communities, whereas HD consistently increased in rhizosphere fungal communities in response to PH‐1 infection (Figure 4o,p; Figure S7o,p).

Overall, DR contributed the most to the root‐associated bacterial community assembly, while HoS contributed the most to the root‐associated fungal community assembly. These findings suggest that bacterial community assembly in wheat roots is more susceptible to stochastic factors, which might compromise community stability. By contrast, fungal community assembly appears to be more influenced by deterministic factors, indicating that pathogen infection imposes selective pressure on wheat root‐associated fungal microbiota.

### 2.6 Conserved and Variety‐Specific Restructuring of the Wheat Rhizosphere and Root Endophytic Microbiomes Following *Fusarium graminearum* Infection

**FIGURE 5:**
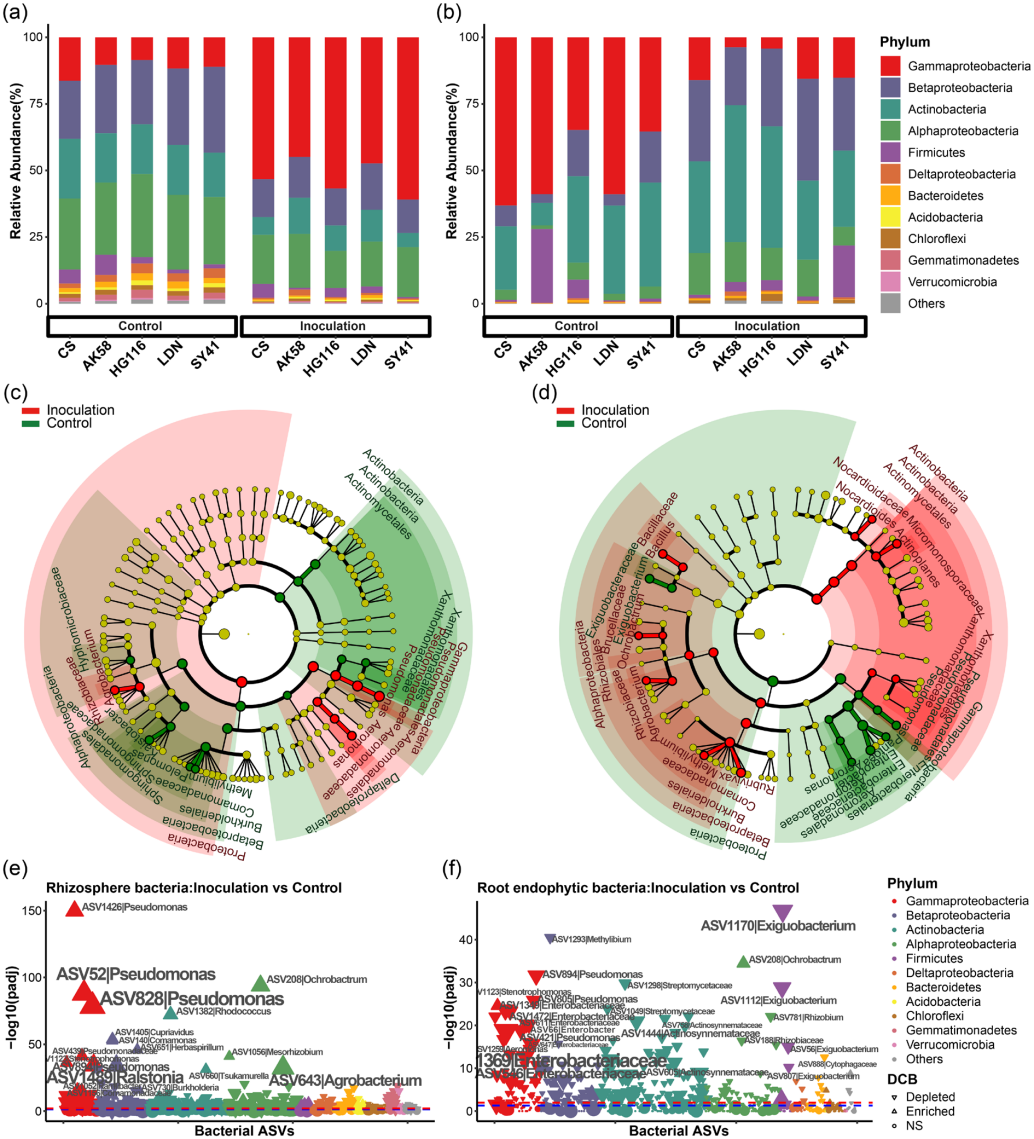
*Fusarium graminearum* infection resulted in wheat root‐associated bacterial microbiota dysbiosis. (a, b) Comparison of the phylum‐level distribution of the rhizosphere (a) and root endophytic (b) bacterial microbiota of wheat varieties with different disease resistances under control and inoculation. Average relative abundance of four biological replicates was displayed in separate stacked bars. (c, d) LDA effect size taxonomic cladogram overall comparing the rhizosphere (c) and root endophytic (d) bacterial microbiota between control and inoculated groups of all wheat varieties. Significantly discriminant bacterial taxon nodes were coloured and branch areas were shaded according to the highest‐ranked variety for that taxon. If the taxon was not significantly differentially represented between sample groups, the corresponding node was coloured yellow. (e, f) Manhattan plots showing the overall variations of Core ASVs in the rhizosphere (e) and root endophytica (f) bacterial microbiota of all wheat varieties following *F. graminearum* infection. Core ASVs that were significantly enriched were depicted as vertical triangles, those that were significantly depleted were depicted as inverted triangles, and others were depicted as circles. The red and blue dashed lines correspond to the FDR‐corrected *p* value of 0.01 and 0.05, respectively. The colour of each point represented the phylum‐level taxonomic affiliation of the core ASVs, and the size corresponds to the baseMean of the core ASVs.

To further evaluate the impact of *F. graminearum* infection on the rhizosphere and root endophytic microbial communities, we compared microbial composition and function between infected and control samples. We observed significant changes in microbial composition across all wheat varieties after inoculation with PH‐1. In the rhizosphere bacterial microbiome, the relative abundance of Gammaproteobacteria significantly increased (*p* = 6.302 × 10^−8^), while that of Betaproteobacteria, Alphaproteobacteria, Actinobacteria, Bacteroidetes, Acidobacteria, Chloroflexi and Gemmatimonadetes significantly decreased (Figure 5a; Table S4a; Kruskal–Wallis test, false discovery rate [FDR]‐adjusted *p* value [*q*] < 0.05). By contrast, in the root endophytic bacterial microbiome, the abundance of Gammaproteobacteria decreased (*p* = 4.229 × 10^−7^), while the abundance of Betaproteobacteria and Alphaproteobacteria increased across all wheat varieties (Figure 5b; Table S4b). In the rhizosphere fungal microbiome, the abundance of Mortierellomycota consistently decreased in all wheat varieties (*p* = 1.783 × 10^−7^; Figure S8a; Table S4c). Although the root endophytic fungal microbiome exhibited inconsistent changes across wheat varieties, significant phylum‐level differences were observed for individual varieties (Figure S8b; Table S4d).

Using LEfSe, we identified microbial taxa significantly enriched in the wheat rhizosphere and root endophytic microbiomes following *F. graminearum* infection. In the rhizosphere bacterial microbiomes, Proteobacteria, particularly Gammaproteobacteria (Pseudomonadales, Pseudomonadaceae, *Pseudomonas*; Aeromonadales, Aeromonadaceae, *Aeromonas*) and Alphaproteobacteria (Rhizobiaceae, *Agrobacterium*), were consistently enriched (Figure 5c). In the root endophytic bacterial microbiomes, Actinobacteria (Actinobacteria, Actinomycetales, Nocardioidaceae, *Nocardioides*, Micromonosporaceae, *Actinoplanes*), Bacillaceae (*Bacillus*), Betaproteobacteria (Burkholderiales, Comamonadaceae, *Rubrivivax*, *Methylibium*), Alphaproteobacteria (Rhizobiales, Rhizobiaceae, *Agrobacterium*, Brucellaceae, *Ochrobactrum*) and Xanthomonadales (Xanthomonadaceae) were enriched (Figure 5d). In the rhizosphere fungal microbiomes, Ascomycota, particularly Sordariomycetes (Hypocreales, Nectriaceae, *Fusarium*), and Psathyrellaceae (*Coprinopsis*) were enriched (Figure S8c). In root endophytic fungal microbiomes, Hypocreales, Nectriaceae and *Fusarium* were significantly enriched (Figure S8d).

We also examined variety‐specific enrichment. In the rhizosphere bacterial microbiomes, Aeromonadales (Aeromonadaceae, *Aeromonas*) were enriched in all allopolyploid wheats, while *Agrobacterium* was enriched in HG116, AK58, LDN and SY41. Oxalobacteraceae was enriched in HG116, *Ralstonia* in SY41, and *Enterobacter*, Brucellaceae and *Ochrobactrum* in CS (Figures S9 and S10). In the root endophytic bacterial microbiomes, Micromonosporaceae was enriched in all hexaploid wheats, Nocardioidaceae in HG116 and AK58 and Stenotrophomonas in LDN (Figures S11 and S12). In the rhizosphere fungal microbiomes, Ascomycota (Hypocreales, Nectriaceae and *Fusarium*) were enriched in HG116, LDN, SY41 and CS, while Dothideomycetes and Pleosporales were enriched in HG116, LDN and CS. Psathyrellaceae, *Coprinopsis*, Pleosporaceae and *Alternaria* were specific to HG116, *Gibberella* to LDN and Agaricales (Bolbitiaceae, *Conocybe*) and Sordariales (Chaetomiaceae) to SY41 (Figures S13 and S14). In the root endophytic fungal microbiomes, Dothideomycetes were enriched in HG116 and LDN, Hypocreales (Nectriaceae, *Fusarium*) in HG116 and SY41, and Ustilaginomycetes (Ustilaginales) in LDN and AK58 (Figures S15 and S16). These findings demonstrate that *F. graminearum* infection significantly enriched specific microbial taxa in both the rhizosphere and root endophytic microbiomes, with enrichment patterns varying among wheat varieties.

We analysed the most discriminating bacterial and fungal ASVs (amplicon sequence variants) between control and inoculation groups using DESeq2 and Manhattan plots. In the rhizosphere microbiota, 767 bacterial and 263 fungal core ASVs exhibited significant changes (*p*_adj_ < 0.05) after *F. graminearum* inoculation (Table S5a,b). Among bacterial ASVs, those from Gammaproteobacteria, Betaproteobacteria, Actinobacteria and Alphaproteobacteria were predominantly enriched (Figure 5e, Table S5a), while fungal ASVs were primarily from Sordariomycetes (Figure S8e, Table S5b). In rhizosphere bacterial microbiota, *Pseudomonas* (ASV52, ASV828, ASV1426, ASV894, ASV421), *Aeromonas* (ASV210) and *Stenotrophomonas* (ASV1123) within Gammaproteobacteria were significantly enriched across all wheat varieties post‐infection (Figures S9 and S10; Table S5e). Additionally, *Ochrobactrum* (ASV208), *Mesorhizobium* (ASV1056) and *Agrobacterium* (ASV643) from Alphaproteobacteria; *Cupriavidus* (ASV1405), *Comamonas* (ASV140), *Herbaspirillum* (ASV651), *Ralstonia* (ASV1489) and *Burkholderia* (ASV730) from Betaproteobacteria and *Rhodococcus* (ASV1382) and *Tsukamurella* (ASV660) from Actinobacteria were enriched in all wheat varieties (Figures S9 and S10; Table S5e). Variety‐specific enrichments included *Rubrivivax* (ASV777, ASV1122) in LDN and AK58, *Bacillus* (ASV169, ASV1329) in HG116 and CS, and Enterobacteriaceae (ASV1349), which was enriched in HG116 but depleted in LDN and SY41. Depleted ASVs included Comamonadaceae (ASV1156) in HG116, LDN, SY41 and AK58 and *Ramlibacter* (ASV1052) in HG116, LDN, SY41 and CS (Figures S9 and S10; Table S5e). In the rhizosphere fungal microbiota, Fusarium ASVs, particularly ASV88 (Sordariomycetes), were enriched in all wheat varieties, with additional enrichment of *Gibberella* (ASV418) in HG116, LDN, SY41 and CS and *Verticillium* (ASV588) in LDN, AK58 and CS (Figures S13 and S14; Table S5f). Enrichment of Sordariomycetes ASVs, such as Sordariales (ASV91), *Magnaporthiopsis* (ASV16) and Chaetomiaceae (ASV43), was higher in SY41. Basidiomycota ASVs such as *Coprinopsis* (ASV715) and *Moesziomyces* (ASV26) were enriched in specific varieties, whereas Ceratobasidiaceae (ASV711) and Auriculariales (ASV287) were depleted (Figures S13 and S14; Table S5f).

In the root endophytic microbiota, 320 bacterial and 209 fungal core ASVs showed significant changes (*p*_adj_ < 0.05) (Table S5c,d). Most bacterial ASVs, including *Pseudomonas* (ASV421, ASV894) and Enterobacteriaceae (ASV1369), were depleted across all wheat varieties, whereas only 22 bacterial ASVs, such as *Pseudomonas* (ASV828), *Ochrobactrum* (ASV208) and *Rubrivivax* (ASV1122), were enriched across all varieties (Figure 5f; Figures S11 and S12; Table S5g). Fungal ASVs showed similar patterns, with enrichment of *Fusarium* (ASV88), *Moesziomyces* (ASV26) and *Acremonium* (ASV554) across all varieties, while *Periconia* (ASV86) was depleted (Figures S8f, S15 and S16; Table S5h). These results highlight significant shifts in the rhizosphere and root endophytic microbial communities following *F. graminearum* inoculation, with taxon‐specific enrichments and depletions varying across wheat varieties.

Functional profiling using PICRUSt2 revealed significant changes in 65.67% of MetaCyc pathways in the rhizosphere bacterial microbiomes and 48.39% in the root endophytic bacterial microbiomes (Figure S17; Table S6). In the rhizosphere bacterial microbiota, PH‐1 infection led to significant changes in pathways across all wheat varieties (Figures S9 and S10), with common enhancement of arginine, ornithine and proline interconversion and L‐arginine degradation II (AST pathway). Variety‐specific changes predominantly involved aromatic compound degradation, carbon metabolism (e.g., TCA cycle VII) and amino acid degradation, suggesting that coordinated metabolic reprogramming occurred under stress, with variety‐specific differences. In the root endophytic bacterial microbiota, no common pathways were significantly altered across all varieties (Figures S11 and S12), with changes primarily involving carbon metabolism, amino acid biosynthesis/degradation and stress‐response pathways, showing distinct metabolic shifts for each variety.

FUNGuild analysis revealed functional shifts in rhizosphere and root endophytic fungal microbiota across wheat varieties following PH‐1 infection. In the rhizosphere, the proportion of the guild Animal Pathogen‐Endophyte‐Fungal Parasite‐Lichen Parasite‐Plant Pathogen‐Wood Saprotroph increased, while Endophyte‐Litter Saprotroph‐Soil Saprotroph‐Undefined Saprotroph decreased across all varieties (Figure S18a). Specific guild changes varied by variety, with, for example, a decrease in Undefined Saprotroph in CS, AK58, HG116 and SY41 but an increase in LDN, and a decrease in Animal Pathogen‐Dung Saprotroph‐Endophyte‐Epiphyte‐Plant Saprotroph‐Wood Saprotroph in CS and AK58 but an increase in HG116, LDN and SY41. In the root endophytes, Animal Pathogen‐Endophyte‐Fungal Parasite‐Lichen Parasite‐Plant Pathogen‐Wood Saprotroph increased in CS, AK58, HG116 and SY41 but decreased in LDN, while Undefined Saprotroph increased in CS and LDN but decreased in AK58, HG116 and SY41 (Figure S18b). Variations in functional groups were inconsistent among varieties and reflected changes in fungal community composition. These results suggest that *F. graminearum* infection significantly alters the functional profiles of wheat root‐associated fungal microbiomes.

### 2.7 Disease Tolerance in Synthetic Hexaploid Wheat is Linked to Resilience Against Gram‐Positive and Gram‐Negative Microbiota Dysbiosis

The above results indicate that *F. graminearum* infection significantly disturbed the rhizosphere and root endophytic bacterial communities, prompting us to compare *F. graminearum*‐tolerant/‐resistant hexaploid wheats (HG116 and AK58) and *F. graminearum*‐susceptible varieties (CS, LDN and SY41). In the rhizosphere, HG116 and AK58 showed higher relative abundances of Gram‐positive Actinobacteria and Firmicutes compared to the disease‐susceptible diploid wheat SY41 (Figure 5a, Table S4a). The most pronounced differences were observed in the root endophytic bacterial microbiota, where HG116 and AK58 displayed increased abundance of Gram‐positive bacteria (Actinobacteria and Firmicutes) and decreased abundance of Gram‐negative bacteria (Gammaproteobacteria and Betaproteobacteria) compared to susceptible varieties LDN and CS (Figure 5b, Table S4b). Compared to SY41, HG116 showed increased abundance of Gram‐positive bacteria (Actinobacteria) and decreased abundance of Gram‐negative bacteria (Gammaproteobacteria), while AK58 showed increased abundance of Gram‐positive bacteria (Actinobacteria) and decreased abundance of Gram‐negative bacteria (Gammaproteobacteria and Betaproteobacteria) (Figure 5b, Table S4b). This balance in microbial homeostasis, characterised by the enrichment of protective Gram‐positive bacteria, appears to be critical for disease tolerance.

LEfSe analysis highlighted distinct Gram‐positive biomarkers, primarily Actinobacteria and Firmicutes, in the root‐associated microbiomes of HG116 and AK58 after infection, with higher occurrence of those belonging to Actinobacteria (Figures S19–S22). Differential Core ASV analysis (DESeq2) revealed significant decreases in Gammaproteobacteria and Betaproteobacteria and increases in Actinobacteria, particularly in root endophytes, of HG116 and AK58 compared to all susceptible varieties (Figures S23 and S24, Table S7a,b). HG116 also showed an increase in Firmicute in the rhizosphere bacterial microbiota compared to its parents LDN and SY41 (Figure S23b,d and Table S7a). Functional comparative analysis revealed that HG116 and AK58 did not share the changes in the same MetaCyc pathways in their rhizosphere bacterial microbiota compared to all susceptible varieties. HG116 and AK58 showed consistent changes in some MetaCyc pathways only compared to SY41 (Figures S25 and S26). However, in the root endophytic bacterial microbiota, compared to LDN, most MetaCyc pathways showed consistent variations in HG116 and AK58. Compared to SY41, both HG116 and AK58 exhibited enhanced octane oxidation and reduced mixed acid fermentation. Compared to all susceptible varieties, HG116 demonstrated enhanced L‐methionine biosynthesis I and a depletion in the superpathway of purine nucleotide de novo biosynthesis II. Similarly, AK58 showed enhanced L‐methionine biosynthesis I and L‐methionine biosynthesis III, respectively, compared to LDN and CS, and a depletion in the superpathway of purine nucleotide de novo biosynthesis II compared to LDN and SY41. In addition, compared to all susceptible varieties, AK58 displayed a decrease in pyrimidine deoxyribonucleotide de novo biosynthesis III, which was also observed in HG116 compared to LDN and CS (Figures S27 and S28). These results indicate that when encountering pathogen invasion, the root‐associated bacterial microbiota of HG116 and AK58 exhibited some unique functions but a certain degree of consistency, which might be crucial for their abilities to defend against diseases capabilities. Together, these results indicate that the SHW HG116 achieved disease‐tolerant levels comparable to disease‐resistant levels of domesticated AK58, largely by enriching Actinobacteria and Firmicutes, thereby mitigating microbial dysbiosis induced by *F. graminearum*. These findings suggest that maintaining homeostasis between Gram‐positive and Gram‐negative bacteria in root‐associated microbiomes is a key factor in disease tolerance and resistance in wheat.

### 2.8 Gram‐Positive Bacteria Function as Key Hubs in Stabilising the Wheat Root Microbiome Under *Fusarium graminearum* Infection

**FIGURE 6:**
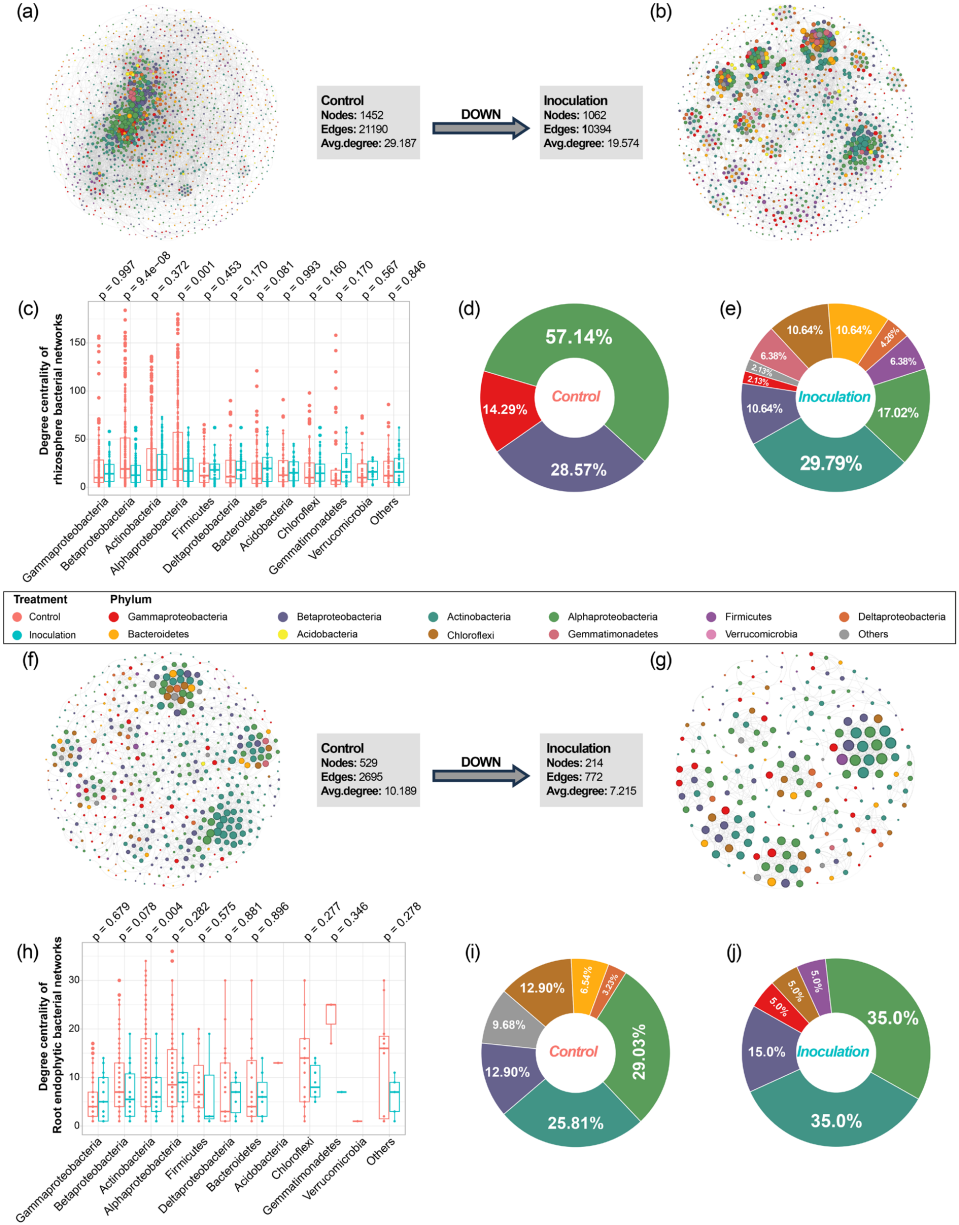
*Fusarium graminearum* infection altered wheat root‐associated bacterial microbial networks. (a, b, f and g) The rhizosphere (a and b) and root endophytic (f and g) bacterial co‐abundance networks of control (a, f) and inoculated (b, g) groups of all wheat varieties on the whole. A connection represented a significant correlation (Spearman's correlation test, *R* > 0.6, *PFDR* < 0.05). Nodes belonging to members of Gammaproteobacteria, Betaproteobacteria, Actinobacteria, Alphaproteobacteria, Firmicutes, Deltaproteobacteria, Bacteroidetes, Acidobacteria, Chloroflexi, Gemmatimonadetes and Verrucomicrobia were coloured. The size of each node was proportional to the degree of each node. Avg. degree was the average of the degrees across all nodes in the rhizosphere bacterial co‐abundance networks. (c, h) Comparing the distribution of degree centrality of bacterial phyla in rhizosphere (c) and root endophytic (h) bacterial co‐abundance networks between control and inoculated groups of all wheat varieties. Statistical significance was determined via Wilcoxon test and with FDR correction (*α* = 0.05). (d, e, i and j) Pie graph showed the proportion of bacterial phyla appearing in hub nodes of the rhizosphere (d and e) and root endophytic (i and j) bacterial co‐abundance networks of control (d and i) and inoculated (e and j) groups. The colour of each slice corresponded to the colour of the node in the rhizosphere bacterial co‐abundance networks.

To assess the impact of *F. graminearum* infection on the stability of the root‐associated microbiome, we constructed microbial co‐abundance networks using core ASVs (Spearman's correlation, *R* < 0.6, *PFDR* < 0.05). The complexity of the bacterial networks in the rhizosphere and root endophytic population significantly decreased after infection, with reductions of 26.9%, 50.9% and 32.9% in nodes, edges and average degree in the rhizosphere and 59.5%, 71.4% and 29.2% in root endophytes, respectively (Figure 6a,b,f,g). By contrast, fungal networks increased in complexity, with 1.34‐, 4.32‐ and 3.21‐fold increases in nodes, edges and average degree for the rhizosphere fungal network and increases of 24.9%, 83.8% and 47.1% for the root endophytic fungal networks, respectively (Figure S29a,b,f,g). These findings reveal that *F. graminearum* infection restructures the root‐associated micro‐ecology by differentially modulating bacterial and fungal interaction networks. The significant reduction in bacterial network nodes and edges indicates the loss of keystone taxa—highly connected taxa essential for maintaining disease‐suppressive functions through metabolic cross‐talk and quorum sensing. The marked decline in their degree centrality corresponds to diminished functional redundancy, impairing the bacterial community's capacity for competitive exclusion of pathogens. Concurrently, the enhanced fungal network connectivity reflects pathogen‐mediated restructuring of interactions among hub fungal taxa, potentially facilitating niche occupation via self‐reinforcing interaction hubs. Collectively, these dual effects—bacterial network destabilisation and fungal alliance consolidation—disrupt plant‐protective microbial barriers, establishing an ecological niche conducive to pathogen dominance.

Actinobacteria emerged as key hub nodes in the rhizosphere bacterial network post‐infection, comprising 29.8% of hubs despite their absence as hubs in healthy controls. In the root endophytic networks, Actinobacteria hubs increased by 35.6%, reaching 35.0% of total hubs, even as their degree centrality decreased (Figures 6c–e, 6h–j). This suggests a functional prioritisation of Actinobacteria under pathogen pressure, with their persistent hub dominance implying functional redundancy—a trait linked to their genomic capacity for diverse antimicrobial compound synthesis and stress‐resistant sporulation. Concurrently, Firmicutes appeared as novel hubs in both the rhizosphere (6.4%) and root endophytic (5.0%) networks (Figure 6e,j). The collective dominance of these Gram‐positive hubs underscores their role in maintaining microbiome integrity via antagonistic metabolite production and structural resilience during *F. graminearum* invasion, highlighting their critical function as stabilisers of dysbiotic root‐associated microecosystems and promising targets for microbiome‐based disease management. In the fungal networks, *F. graminearum* infection increased the degree centrality of ASVs from Sordariomycetes, Basidiomycota, Dothideomycetes and Glomeromycota in both the rhizosphere and root endophytes (Figure S29c,h). Although the proportion of Sordariomycetes hubs decreased, they remained dominant among rhizosphere fungal hubs, while new hubs emerged from Basidiomycota, Eurotiomycetes and Chytridiomycota (Figure S29d,e). Root endophytic fungal hubs exhibited declines in Sordariomycetes, Basidiomycota, Dothideomycetes and Pezizomycetes but increases in Eurotiomycetes, Mortierellomycota and Glomeromycota (Figure S29i,j). The increased fungal hub diversity and network complexity post‐infection reflect *F. graminearum*'s dual ecological strategy: expanded interactions among Basidiomycota (resource decomposers) and Glomeromycota (symbionts) may enhance microbiome stability through niche complementarity, whereas the pathogen's retention of Sordariomycetes hubs (including Fusarium relatives) and recruitment of stress‐adapted taxa like Eurotiomycetes (secondary metabolite producers) and Mortierellomycota (lipid specialists) likely drive synergistic resource competition—liberating nutrients for pathogen proliferation while suppressing bacterial antagonists. This restructured network establishes a self‐reinforcing equilibrium where heightened connectivity temporarily buffers ecosystem collapse but entrenches pathogen dominance through fungal guild co‐option. Together, these dynamics demonstrate that fungal network complexity under pathogen invasion serves dual roles—stabilising microbial community structure while paradoxically promoting pathogen success in dysbiotic root ecosystems.

### 2.9 Pathogen Infection Alters Wheat Root Microbiome Structure by Consolidating Microbial Interactions With Dominant Bacteria

**FIGURE 7:**
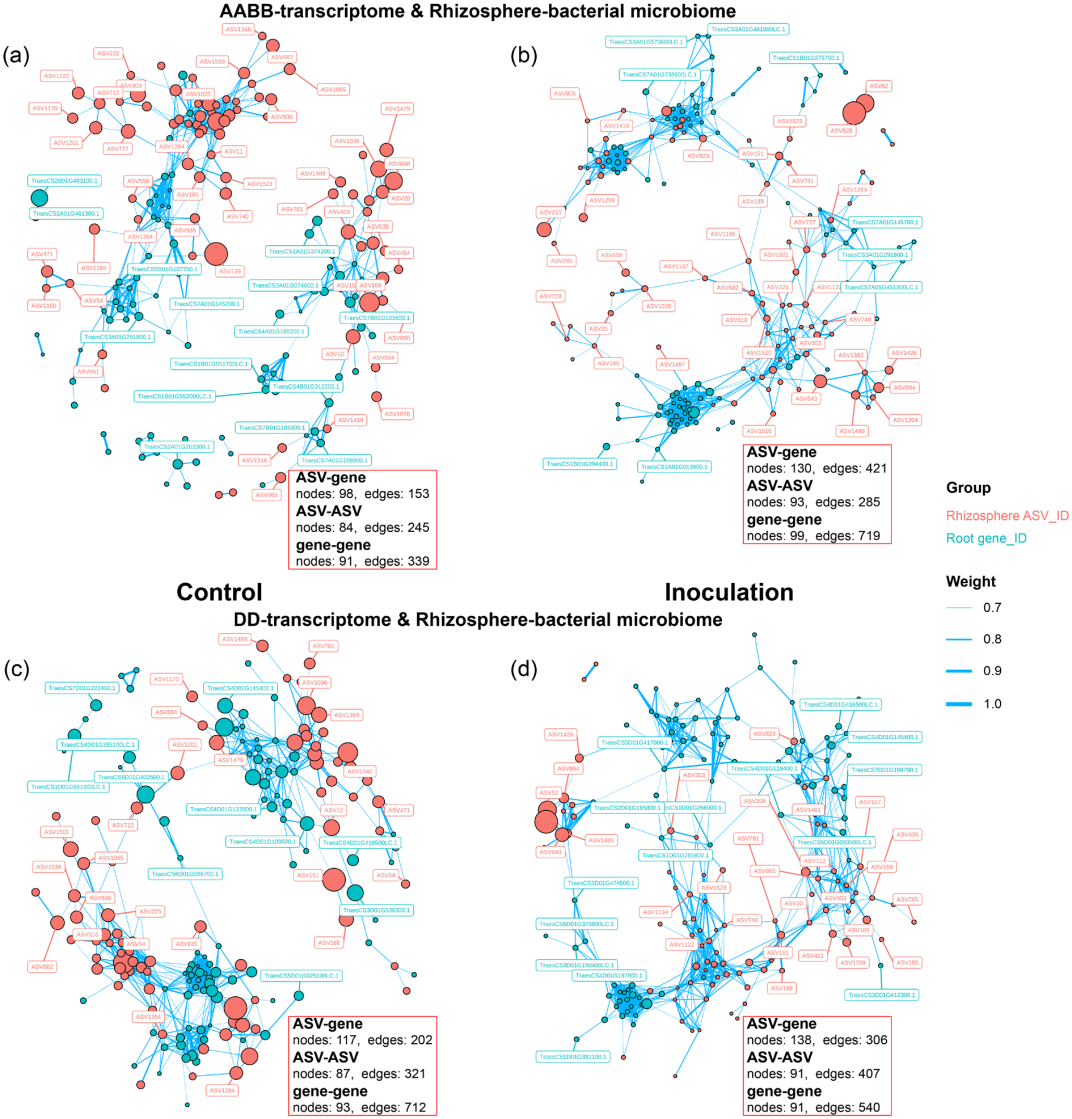
The correlation networks between the rhizosphere bacterial microbiome and root transcriptome. (a–d) The correlation networks between the rhizosphere bacterial microbiome and root transcriptome of wheats with AABB (a and b) subgenomes and with DD subgenome (c and d), under control (a and c) and inoculation (b and d). The rhizosphere bacterial core ASVs were indicated in salmon colour, and wheat root genes were indicated in dark turquoise. Weight indicated the significant correlation between network nodes (Spearman's correlation test, *R* > 0.07, *p* < 0.01). The size of each node indicated the relative abundance of each rhizosphere bacterial core ASV or the expression level of each root gene. ASV‐gene represented the correlation subnetwork between wheat rhizosphere bacterial microbiota and genes; ASV–ASV represented the correlation subnetwork among wheat rhizosphere bacteria, and gene–gene represented the correlation subnetwork among wheat root genes.

Correlation network analysis between root‐associated microbiomes and the root transcriptome following *F. graminearum* infection revealed distinct changes in network complexity and community dynamics. In the rhizosphere, bacterial network complexity increased, with interactions shifting from multiple high‐/moderate‐abundance ASVs to many low‐abundance ASVs (Figure 7a–d; Figures S30a and S31a–d). Stochastic processes dominated rhizosphere bacterial community dynamics, with deterministic contributions decreasing in high‐abundance bacterial assembly but increasing in moderate‐ and low‐abundance communities (Figure S30c,e). This transition suggests that enhanced plant interactions with low‐abundance bacteria may destabilise rhizosphere community structure through unregulated stochastic colonisation.

Conversely, the complexity of the root endophytic bacterial network decreased, shifting to interactions with fewer high‐/moderate‐abundance ASVs (Figures S30b, S31e–h, S32a–d). Deterministic processes intensified in high‐ and low‐abundance endophytic bacterial assemblies (Figure S30d,f), indicating active plant selection—particularly enrichment of dominant stress‐adapted Gram‐positive taxa (Actinobacteria, Firmicutes) for pathogen suppression, while restricting non‐essential low‐abundance bacteria through immune‐mediated filtering.

In the fungal communities, *F. graminearum* infection reduced rhizosphere fungal network complexity in wheats with the AABB subgenomes, disrupting moderate‐abundance fungi while increasing the accumulation of lower‐abundance fungi, a process driven by slight increases in deterministic processes for low‐abundance fungi (Figures S33a,b; S34a,c; S35a,b). By contrast, wheat with the DD subgenome exhibited increased network complexity, recruiting moderate‐ and low‐abundance fungi with potential antagonistic effects on *F. graminearum* (Figures S33c,d; S34a,e; S35c,d). For the root endophytic fungal communities, interactions shifted to focus on low‐abundance and individual dominant ASVs, with deterministic processes increasing in low‐abundance fungal assemblies but decreasing in high‐/moderate‐abundance communities for wheats with both types of genomes (Figures S34b,d,f; S35e–h; S36a–d). This niche‐focused restructuring likely prioritises functionally critical taxa while suppressing pathogen‐compatible fungi. Collectively, subgenome‐specific recruitment of beneficial microbes and niche‐driven community reorganisation represent key plant strategies to balance microbiome homeostasis and enhance pathogen tolerance under infection stress.

## 3 Discussion

### 3.1 Synthetic Polyploidisation Enhances *F. graminearum* Tolerance

As hexaploid wheat cultivars have superior grain traits and adaptability compared with their ancestors, amounts of SHW have been generated through hybridisation between tetraploid wheat and *Ae. tauschii*, which was considered an important bridge to transfer favourable alleles or desirable traits from both tetraploid wheat and *Ae. tauschii* into wheat (Li et al. 2018; Hao et al. 2020). Previously, newly synthesised hexaploid wheat was not only found to inherit traits such as grain length, plant height, plant architecture and endosperm starch composition of tetraploid wheat, but also displayed superior growth vigour in some traits compared with parental lines, including larger spikes and more robust seedling growth (Li et al. 2014). Consistently, the current SHW line HG116 also exhibited similar agronomic traits to tetraploid wheat, such as plant height, tiller number and awn length, and a significantly longer spikes trait, larger kernel area, and perimeter than LDN in both the current study and the previous report (Li et al. 2016). Therefore, wheat polyploidisation could affect many agronomic traits of SHW, mostly exhibiting the trait basis of tetraploid wheat and domestication traits of *Ae. tauschii*.

Furthermore, in this study, we discovered that synthetic polyploidisation enhanced tolerance of *F. graminearum* in SHW. HG116 and AK58 consistently exhibited significantly milder seedling leaf symptoms than CS, LDN and SY41 in our study. Crucially, the observed tolerance phenotypes may be developmental stage‐dependent; these seedling results should not be conflated with FHB resistance in spikes at the adult stage (Yang et al. 2024), as different genetic mechanisms may govern wheat–FHB interactions across developmental stages. Further research is needed to validate HG116's seedling response and elucidate the genetics underlying its stage‐specific disease outcomes.

Unlike disease resistance, which focuses on directly restricting pathogen multiplication, disease tolerance centres on the plant's ability to maintain normal physiological functions, growth and reproduction despite the presence of diseases. In brief, disease tolerance refers to the ability of plants to reduce the negative impacts of disease on their growth and yield by adjusting their physiological and biochemical processes. This ability does not directly confront the pathogen but instead enhances plant resilience, adaptability and stability, allowing plants to maintain good growth status despite disease pressure (Manchester et al. 2010; Maulenbay and Rsaliyev 2024; Pagán and García‐Arenal 2018).

Studies on the disease resistance mechanisms of wheat and other crops have made remarkable progress, including the discovery of resistance genes, the elucidation of signalling pathways associated with disease resistance and the identification of physiological and biochemical indicators and molecular markers related to disease resistance, providing important references for disease resistance breeding. However, applying these findings to crop production to obtain varieties with broad, durable resistance still faces numerous challenges. The diseases encountered during crop production are diverse and constantly evolving. The pathogenic mechanism, infection route and degree of harm differ among diseases, requiring targeted research to breed disease resistance against specific diseases. Most resistance genes discovered to date exhibit specific resistance spectra and performance. Some are only effective against particular diseases or certain physiological races of a disease but ineffective against others. Therefore, it is often difficult to achieve broad, durable disease resistance using a single resistance gene (Pink and Puddephat 1999). Disease resistance is often controlled by a single gene or a major quantitative trait locus, whereas disease tolerance is mainly influenced by multiple loci and their interactions (Kruse et al. 2017; Lozada et al. 2019; Maulenbay and Rsaliyev 2024). These complex interactions among genes collectively regulate the physiological and biochemical mechanisms of plants to enhance their disease tolerance. For instance, plants can achieve disease tolerance through compensatory increases in photosynthesis, an adjustment in resource redistribution from growth to reproduction and changes in phytohormone‐related gene expression (Agnew et al. 2000; Block et al. 2005; Citovsky et al. 2017; Collin et al. 2018; Foulkes et al. 2006; Holmes et al. 2008; Inglese and Paul 2006; Scholes and Farrar 1986; Stare et al. 2015; Zhao et al. 2013). Therefore, disease tolerance confers broader adaptability, allowing plants to cope with the invasion of various pathogens. Since tolerance does not rely on specific resistance genes, it is less susceptible to factors such as disease evolution or genetic recombination. Consequently, disease tolerance might be more durable, maintaining stability over long‐term production practices. Numerous experiments have demonstrated that plant tolerance to pathogens is an efficient, universal defensive strategy against various pathogens that coexists with resistance (Castro and Simón 2016; Foulkes et al. 2006; Jarvie and Shanahan 2009; Mikaberidze and McDonald 2020; Newton 2016; Pagán and García‐Arenal 2018; Parker et al. 2004; Zhan et al. 2002).

Enhancing crop tolerance can help reduce the impact of diseases on agricultural production, improving crop yield and quality and securing food security. Additionally, breeding disease tolerance in crops helps reduce pesticide usage and the cost of agricultural production while promoting sustainable agricultural development (Maulenbay and Rsaliyev 2024). Our findings on the tolerance of SHW to *F. graminearum* provide insight for targeted breeding of wheat.

### 3.2 Upregulated Dehydrin Gene Expression in Roots Enhances the Disease Tolerance of Synthetic Hexaploid Wheat

In this study, transcriptome analysis revealed high levels of expression of many dehydrin genes in the roots of SHW following inoculation with *F. graminearum* strain PH‐1 (Figure 3d,e; Figure S2a). Genome duplication events (polyploidisation and local duplication) drive the expansion of the wheat dehydrin gene family by enhancing gene expression potential through dosage effects, accompanied by subgenome coordination and functional divergence, which may constitute the molecular basis for the burst expression of dehydrin genes during synthetic hexaploid wheat's defence against *F. graminearum* infection (Allagulova et al. 2003; Ezoe et al. 2024; Xie et al. 2023; Yu et al. 2024).

Most studies on dehydrin genes have focused on their roles in protecting plants from abiotic stress, whereas few studies have examined the direct link between dehydrin genes and biotic stress. Turco et al. (2004) discovered that *Phytophthora cinnamomi* infection induced the production of dehydrin‐like proteins in Turkey oak ( *Quercus cerris* ) and holm oak ( *Quercus ilex* ), supporting our finding that plant pathogen infection can induce the expression of plant dehydrin genes. Salicylic acid (SA) and jasmonic acid (JA) induce the expression of various dehydrin genes, such as *BcDh2* from drought‐tolerant *Boea crassifolia* and *DHN5* from barley ( *Hordeum vulgare* ) (Shen et al. 2004; Sun et al. 2009). Both SA and JA play important roles in plant resistance to pathogen invasion, and both phytohormones enhance FHB resistance in wheat (Kachroo and Kachroo 2007; Makandar et al. 2012; Zhang et al. 2018), suggesting that dehydrins might function in plant defence against biotic stress. Brini et al. (2011) performed transcriptome profiling using the Affymetrix ATH1 microarray and heterologously expressed the wheat dehydrin gene *DHN‐5* in Arabidopsis ( *Arabidopsis thaliana* ) to explore potential targets of DHN‐5 that might improve stress tolerance. The authors identified various genes involved in abiotic as well as biotic stress tolerance. Notably, some genes involved in pathogen responses (*PR* genes) were among the most strongly upregulated genes, suggesting that DHN‐5 might modulate the defence response of Arabidopsis against pathogens by activating certain *PR* genes. Furthermore, two WRKY transcription factor genes were downregulated in these plants. WRKY transcription factors participate in defence responses to bacteria and necrotrophic fungal pathogens (Li et al. 2006; Zheng et al. 2006), suggesting that DHN‐5 might also be involved in responses to bacterial and necrotrophic fungal pathogens. Moreover, three genes encoding jasmonate‐ZIM domain (JAZ) proteins, which negatively regulate JA signalling, were strongly downregulated by *DHN‐5* overexpression and positively regulated by MYC2, suggesting that DHN‐5 promotes the expression of some JA‐responsive genes involved in pathogen defence by disturbing MYC2‐dependent transcription. These findings suggest that dehydrin genes enhance disease tolerance by regulating plant defence‐related signalling pathways.

Dehydrins also possess antibacterial properties, as they inhibit the growth of some Gram‐negative bacteria, such as *Escherichia coli* and *Agrobacterium tumefaciens* ; Gram‐positive bacteria such as *Staphylococcus aureus* , *Bacillus pumilus* , *Bacillus subtilis* and *Sarcina lutea*; and fungi such as *F. graminearum*, *Aspergillus niger*, *Botrytis cinerea* and *Alternaria solani*. The K‐segments of dehydrins play major roles in these inhibitory effects (Drira et al. 2016, 2015; Zhai et al. 2011). K‐segments are rich in lysine and form amphiphilic α‐helices (Close 1996; Malik et al. 2017), allowing them to bind to lipid components on biological membranes and the hydrophobic sites of partially denatured proteins, thereby functioning as membrane protectors and molecular chaperones (Brini et al. 2014; Hara et al. 2001). Several α‐helical structures capable of binding to bacterial biofilms have been discovered. For example, certain antimicrobial peptides (AMPs) have an α‐helix structure that can disrupt the phospholipid bilayer structure of bacteria, thus interacting with bacterial biofilms. Moreover, α‐helical structures with different structures and functions could be synthesised by changing the sequence positions of amino acids, increasing their biofilm penetration and clearance capabilities. Therefore, perhaps the K‐segments of dehydrins interact with the cell membrane of *F. graminearum* by forming α‐helical structures, thereby inhibiting pathogens and protecting SHW from pathogen infection.

Interestingly, the robust induction of dehydrin genes in wheat roots following *F. graminearum* infection may involve a potential interplay with extracellular ROS actively generated by the pathogen during invasion. Studies have shown that *F. graminearum* explosively releases extracellular ROS in later infection stages through self‐synthesised enzymatic systems such as NADPH oxidases, triggering oxidative damage in plant cells (Zhang et al. 2012). In response, host plants likely activate dehydrin gene expression via ROS signalling pathways (e.g., MAPK cascades) to mitigate ROS‐induced destruction of cellular membranes and proteins, thereby enhancing oxidative stress tolerance. As an adaptive response to ROS challenge, dehydrins may buy time for plants to mobilise other immune and defence mechanisms, such as the synthesis of antimicrobial peptides (e.g., defensins) and pathogenesis‐related (PR) proteins, which collectively reinforce the barrier against *F. graminearum* colonisation and disease progression.

We hypothesised that SHW can synergistically counteract *F. graminearum* infection through a dehydrin‐mediated multi‐layered defence network (Allagulova et al. 2003; Rorat 2006). Active disease resistance level: Dehydrins induce defence gene expression by regulating JA/SA signalling pathways while directly inhibiting *F. graminearum* via K‐segment‐mediated membrane‐lytic activity. Cellular tolerance level: Dehydrins maintain membrane stability, scavenge ROS, chelate metal ions and regulate osmotic balance to preserve normal physiological functions of SHW cells under pathogen stress. Ecological synergy level: Dehydrins remodel the rhizosphere microbiome by enriching beneficial microbial communities to suppress pathogen spread. Polyploidy‐enhanced mechanism: SHW's gene dosage effects and subgenome‐coordinated expression significantly amplify the dual efficacy of dehydrins in disease resistance and tolerance, enabling both active pathogen defence and cellular homeostasis maintenance, ultimately achieving comprehensive improvement in *F. graminearum* tolerance.

### 3.3 Wheat Root‐Associated Microbiota Homeostasis Driven by Gram‐Positive Actinobacteria and Firmicutes Plays a Key Role in Maintaining the Health of Synthetic Hexaploid Wheat

The plant rhizosphere microbiome exhibits noteworthy parallels with the gut microbiome in terms of nutrient absorption, spatial distribution and formation mechanisms (Hacquard et al. 2015). The homeostasis of the gut microbiome is crucial for human health, as it regulates the immune system and metabolic processes, demonstrating great potential in clinical studies on disease intervention (Schmidt et al. 2018).

**FIGURE 8:**
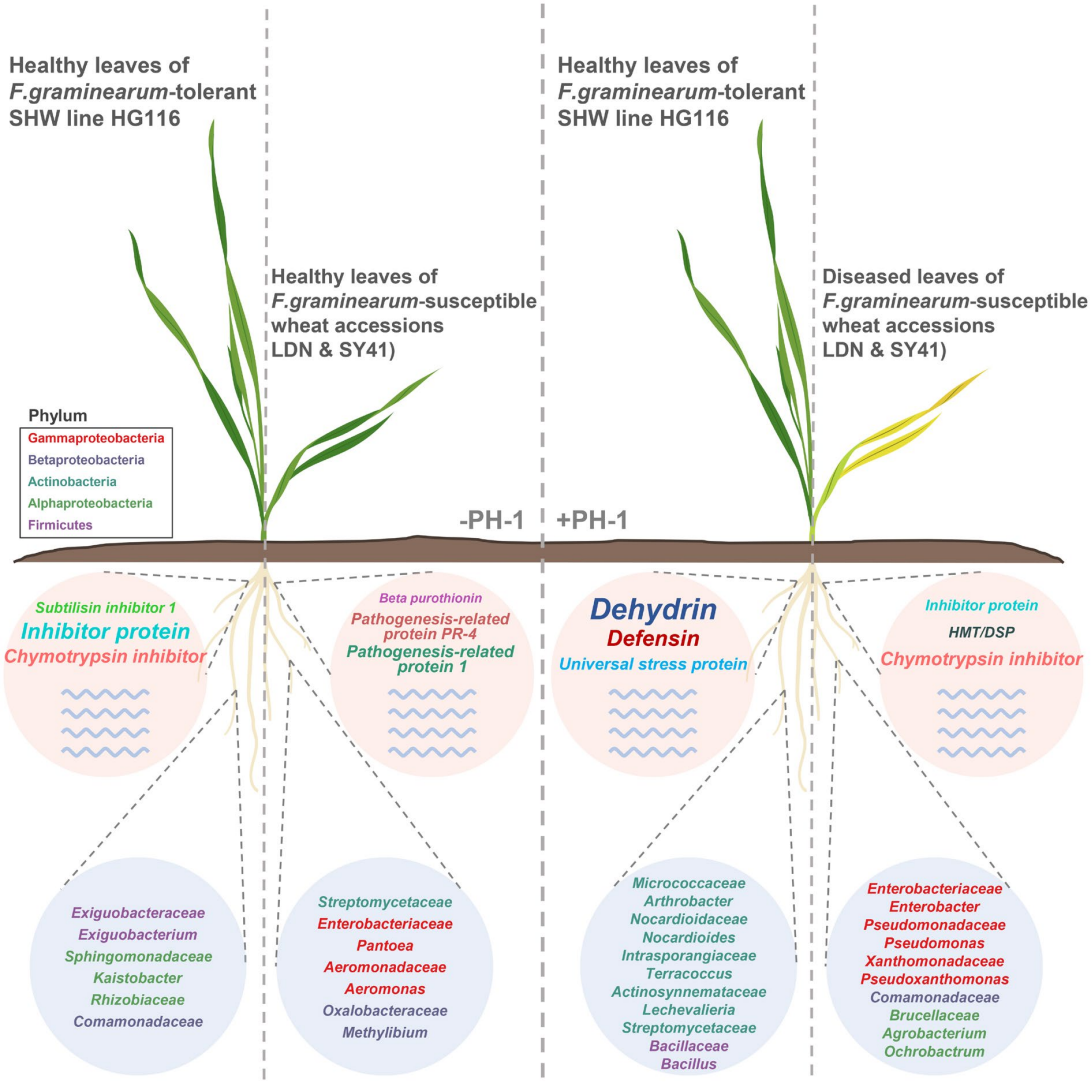
The schematic diagram for illustrating the variation differences in the root transcriptome and root‐associated microbiomes between *Fusarium graminearum*‐tolerant synthetic hexaploid wheat line HG116 and its *F. graminearum*‐susceptible parents LDN and SY41 before and after PH‐1 inoculation. This schematic diagram shows the changes of DEGs significantly enriching in stress‐ and defence‐related pathways between HG116 and its parents LDN and SY41. The sizes of DEGs names were determined by their frequencies of occurrence (for additional details, refer to Figures S37 and S38). Additionally, it displayed the differential root‐associated bacterial biomarkers between HG116 and its parents LDN and SY41 by LEfSe analysis. Due to space limitations, only the family and genus levels are shown here. For details, please refer to Figures S19 and S21. The colours of the biomarkers correspond to their respective phylum. HMT/DSP, heavy metal transport/detoxification superfamily protein. Adapted from ‘Wheat Crop Growth Stages (Zadok's Scale)’, by BioRender.com (2025). Retrieved from https://app.biorender.com/biorender‐templates.

Similarly, homeostasis of the plant rhizosphere microbiome is crucial for plant growth and health. Here, we inoculated wheat materials of different ploidy levels with *F. graminis* strain PH‐1 and observed the increased abundance of Gram‐positive bacteria (Actinobacteria and/or Firmicutes) and decreased abundance of Gram‐negative bacteria (Gammaproteobacteria and/or Betaproteobacteria) in the root‐associated bacterial microbiomes of *F. graminearum*‐tolerant and ‐resistant hexaploid wheats. By contrast, microbiota dysbiosis emerged in *F. graminearum*‐susceptible wheats, characterised by a decrease in Gram‐positive bacteria and an increase in Gram‐negative bacteria (Figure 8; Figures S19–S24).

Microbiota dysbiosis between Gram‐positive and Gram‐negative bacteria negatively affects plant growth and health. Microbiota dysbiosis, characterised by a reduction in Gram‐positive Firmicutes and an increase in Gram‐negative Proteobacteria levels, occurred in the phyllosphere microbiome of Arabidopsis *mfec* (*min7 fls2 efr cerk1*) quadruple mutants; these plants show deficiencies in basic immunity and leaf water balance at high temperature and relative humidity, resulting in leaf chlorosis and/or necrosis (Chen et al. 2020). The abundance of Gram‐positive Actinobacteria and Firmicutes was lower in diseased rhizosphere soil than in healthy rhizosphere soil. Artificial disruption of Gram‐positive bacteria in healthy rhizosphere soil using vancomycin increased the occurrence of bacterial wilt in tomato ( *Solanum lycopersicum* ), indicating that the dysbiosis of Gram‐positive Firmicutes and Actinobacteria in the tomato rhizosphere promotes the onset of bacterial wilt disease (Lee et al. 2020). Microbiota dysbiosis, with depleted Gram‐positive (Actinobacteria and Firmicutes) and enriched Gram‐negative (Proteobacteria and Bacteroidetes) bacteria, was also observed in SGS (Soybean stay‐green syndrome) seeds. However, the specific bacterial genera for dysbiosis were inconsistent across soybean varieties, suggesting that microbiota dysbiosis might be a concurrent phenomenon rather than a causative factor in SGS. Finally, a survey of the microbiomes of wheat plants associated with three common diseases, wheat stem rot, wheat stripe rust and wheat sharp eyespot, also uncovered microbiota dysbiosis (Wang, Wang, Wang, et al. 2022). These findings align with our results, confirming that microbiota dysbiosis is often associated with plant diseases. The maintenance of microbiota homeostasis driven by Gram‐positive Actinobacteria and Firmicutes in the root‐associated bacterial microbiome helps maintain plant health (Figure 8). Our findings lay the foundation for designing defensive microbiomes to enhance plant fitness against diseases, providing a reliable, safe, environmentally friendly control method for agricultural production with great application potential in the field.

### 3.4 Dual‐Mode Pathogen Defence Strategies Mediated by Polyploidy: Microbial Clearance and Host Tolerance

In recent years, polyploidy has emerged as a novel perspective in plant disease resistance regulation, garnering increasing research attention. However, studies linking polyploidisation with plant‐associated microbiomes to investigate their combined effects on host disease resistance remain scarce. The pioneering work by Mehlferber et al. (2022) in Arabidopsis revealed that polyploidisation enhances resistance to bacterial pathogens through phyllosphere microbiome remodelling and activation of the JA pathway. In contrast, our study demonstrates that SHW acquires tolerance to *F. graminearum* by maintaining rhizosphere microbiome homeostasis and upregulating dehydrin gene expression. Although both studies focus on polyploidisation‐driven disease resistance benefits, their mechanisms exhibit significant divergence and underlying convergence in microbiome selection, genetic regulation and defence strategies, providing a multidimensional perspective for understanding polyploidy's role in plant–pathogen interactions.

As an evolutionary innovation for coping with pathogen stress, polyploidisation manifests diverse disease resistance pathways across species. In autotetraploid Arabidopsis, polyploidisation leads to significant enrichment of Gram‐negative Proteobacteria (particularly *Pseudomonas*) in the phyllosphere microbiome, which directly suppresses foliar bacterial pathogens (e.g., *Pseudomonas syringae* ) via siderophore competition and antibiotic secretion (Mehlferber et al. 2022), exemplifying a ‘microbiome‐mediated pre‐adaptive basal resistance’. In contrast, our work highlights a ‘microbiome‐mediated stress tolerance adaptation’ in SHW under *F. graminearum* infection, where enrichment of Gram‐positive Actinobacteria and Firmicutes stabilises rhizosphere microbiome homeostasis without direct pathogen antagonism. This comparison reveals polyploidy's dual capacity to preemptively optimise microbial communities against potential threats and dynamically remodel microbiomes under stress, showcasing plants' evolutionary ingenuity in integrating ‘preemptive defence’ and ‘adaptive plasticity’ through genome doubling.

For autotetraploid Arabidopsis, JA pathway activation results from synergistic interactions among polyploid genetic background, microbiome functions and pathogen stress: gene dosage effects provide the foundational capacity for JA pathway activation, enriched microbiomes amplify JA responses via metabolite signalling and pathogen invasion acts as an environmental trigger to initiate defence signalling cascades. These processes collectively enhance JA pathway intensity, forming an ‘active clearance’ resistance centred on direct pathogen suppression, reflecting polyploid plants' evolutionary strategy to amplify disease resistance by integrating ‘host genome‐microbiome‐environmental signals’. Conversely, for SHW, the marked upregulation of dehydrin genes is pivotal for its resisting *F. graminearum*. Rather than directly killing pathogens, this response likely achieves ‘physiological functional preservation under pathogen coexistence’ through dehydrin‐mediated cellular protection (e.g., membrane stabilisation, ROS scavenging, Heavy Metal Chelation) coupled with microbiome‐driven ecological stabilisation. This comparison reveals that polyploidy not only enhances host‐dominant active immunity but also promotes host‐microbe co‐adaptive tolerance. The tolerance strategy identified in allohexaploid wheat against fungal diseases allows low‐level pathogen persistence, thereby reducing selection pressure on resistance genes and delaying pathogen adaptive evolution and establishing an eco‐friendly disease resistance paradigm characterised by ‘coexistence with pathogens’ rather than ‘eradication’. Such a strategy demonstrates heightened evolutionary robustness in managing chronic and systemic diseases (e.g., soil‐borne fungi) and offers a novel framework for designing crops with enhanced stress resilience.

## 4 Conclusions

*F. graminearum* infection alters the diversity, community structure and ecological functions of the wheat root‐associated microbiome, triggering microbiota dysbiosis. Notably, SHW employs a dual defence strategy to protect itself from pathogens: (i) enriching beneficial Gram‐positive bacteria (Actinobacteria and Firmicutes) in its root‐associated microbiome to maintain microbiome homeostasis and (ii) upregulating many dehydrin genes expression in roots (Figure 8). Based on our findings, we propose that synthetic polyploidisation confers SHW with more stable root‐associated microbiomes and broader expression of dehydrin genes, thereby allowing SHW to acquire tolerance to *F. graminearum* not present in its parents. This links polyploidisation with root‐associated microbiomes, highlighting the important role of the interaction between them to enhance pathogen defence in allohexaploid wheat, providing a genomic‐microbial framework for the prevention and control of *F. graminearum*.

## 5 Experimental Procedures

### 5.1 Plant Materials and Growth Conditions

Five wheat accessions were used in this study, including diploid *Ae. tauschii* Coss. accession SY41 (2*n* = 2*x* = 14, DD), tetraploid *T. turgidum* L. ssp. *durum* accession Langdon (LDN; 2*n* = 4*x* = 28, BBAA), model hexaploid wheat variety'Chinese Sprin' (CS; 2*n* = 6*x* = 42, BBAADD), modern cultivated allohexaploid wheat variety AK58 (2*n* = 6*x* = 42, BBAADD) and new SHW line HG116 (2*n* = 6*x* = 42, BBAADD).

Seeds of all wheat varieties were surface sterilised (incubated for 2 min in 75% ethanol, rinsed four times with sterile water, incubated for 10 min in 25% NaClO and rinsed five times with sterile water) and germinated on sterile 1% water agar plates in the dark (48 h at 4°C and an additional 48 h at 25°C). For the pathogen inoculation experiment, wheat seeds with their white radicles exposed were soaked in *F. graminearum* strain PH‐1 suspension (conidial concentration: 1.0–2.0 × 10^6^/mL) for 1 h and then germinated on sterile 1% water agar plates in the dark (24 h at 25°C). All wheat seedlings were simultaneously transplanted into pots containing soil collected from a botanical garden at Nanjing Normal University (N 32°06′31.10″, E 118°54′21.81″), and placed in a greenhouse (16 h light/8 h dark period at 22°C; photon flux density, 250 mmol m^2^/s). The plants were watered with sterile water based on their growth. For the pathogen inoculation experiment, leaf chlorosis and/or necrosis phenotypes were observed and statistically analysed after 1 month.

### 5.2 Collection of Rhizosphere and Root Endophytic Microbial Samples

To isolate the rhizosphere and root endophytic microbial communities, the roots of each plant were cautiously pulled from the soil and separated from the adhering soil particles using sterile tweezers. The roots were placed into 50 mL Falcon tubes containing 25 mL PBS buffer (140 mM NaCl, 2.7 mM KCl, 10 mM Na_2_HPO_4_, 1.8 mM KH_2_PO_4_) and stirred vigorously to remove all the soil from the root surfaces. The soil was then collected after centrifugation (20 min at 3094 × *g*) and treated as rhizosphere soil (Wang, Feng, et al. 2021). Root endophytic samples were obtained after three sonication procedures. For each procedure, the cleaned roots were placed in fresh PBS buffer and sonicated (30 s at 42 Hz, 30 s pause) to remove tightly adhering soil particles. After collecting the root endophytic samples, the remaining clean roots were quickly frozen in liquid nitrogen and stored at −80°C until DNA and RNA extraction. For each wheat genotype, four biological replicates were randomly collected from uninoculated and inoculated samples.

### 5.3 DNA Extraction, High‐Throughput Sequencing of Amplicons (16S and ITS rRNA) and Data Processing

DNA extraction was performed using a FastDNA SPIN Kit for Soil (MP Biomedicals, Irvine, CA, USA). For quantitative microbiome profiling (QMP), the microbial loads (absolute abundances) were estimated using the spike‐in based microbiome profiling method (Wang et al. 2020). Soil‐ and plant‐associated samples were weighed before DNA extraction, and synthetic chimeric DNA spikes were added to the DNA samples. For bacterial QMP, 500 pg (~108 million copies) of 16S synthetic spikes were added to the rhizosphere DNA samples, and 50 pg were added to each root DNA sample. For fungal QMP, the amounts of ITS synthetic spikes added were 1% that for bacterial QMP because of the different estimated microbial loads of the samples.

For high‐throughput sequencing of the 16S rRNA and internal transcribed spacer (ITS) regions, the V5–V7 regions of bacterial 16S rRNA gene sequences were amplified from DNA extracts using the 799F‐1193R (799F: AACMGGATTAGATACCCKG; 1193R: ACGTCATCCCCACCTTCC) universal primers (Bai et al. 2015), and the fungal ITS regions were amplified using the fITS7‐ITS4 (fITS7: GTGARTCATCGAATCTTTG; ITS4: TCCTCCGCTTATTGATATGC) universal primers (Waldor et al. 2016). Library construction and sequencing (Illumina MiSeq, PE 2 × 300 bp) were conducted by Shanghai Hanyu Biotech Co. (Shanghai, China).

Raw Illumina FASTQ files were filtered for quality and their taxonomy analysed using QIIME 2 (Bolyen et al. 2018). Briefly, primers of imported sequences were removed using Cutadapt (Martin 2011). DADA2 (Callahan et al. 2016) was used to filter and denoise the sequences, remove chimeras, identify representative ASV sequences and create an ASV table. For bacterial 16S rRNA gene amplicon sequences, representative ASV sequences were taxonomically annotated using a pre‐trained Naive Bayes classifier based on the bacterial 16S rRNA Greengenes reference database (13_8 release) (DeSantis et al. 2006) and the sequences of 16S synthetic spikes. From this taxonomic annotation, all unassigned sequences and sequences annotated as mitochondria, chloroplasts, and Archaea were removed. Finally, the taxonomic information for the representative bacterial ASVs was annotated with RDP Classifier (using 16S rRNA training set 18) (Wang et al. 2007), which provides copy number adjustment for 16S rRNA gene sequences. For fungal ITS amplicon sequences, representative ASV sequences were taxonomically annotated using the fungal ITS classifiers trained on the UNITE reference database (Nilsson et al. 2019) and the sequences of ITS synthetic spikes. From this taxonomic annotation, only sequences annotated as fungi were retained. The metadata and sequencing stats for bacterial and fungal microbiome profiling are provided in Table S9. QMP of bacteria and fungi was performed based on the number of spike reads and whole bacterial and fungal reads. The absolute quantification of genes (16S and ITS rRNA) for each ASV per gram of sample (QAGA) was estimated using this equation: QAGAi = S/SS*SAi, where i represents the IDs of each ASV, S is the number of synthetic spikes added per gram of sample, SS is the number of sequenced reads assigned to synthetic spikes, and SA is the number of sequenced reads assigned to each ASV (Wang, Feng, et al. 2021). The phrase ‘PH‐1 ITS copies’ denotes the absolute abundance of ITS for PH‐1 per gram of sample, which was also quantified using this methodology. After the synthetic spike reads were removed, the filtered ASV sequences and the resulting ASV table were used to determine taxonomic distributions and alpha (Shannon's species diversity and Faith's phylogenetic diversity indices) and beta (Bray‐Curtis distances and weighted UniFrac distances) diversities. For alpha and beta diversity calculations, samples were rarefied to the same number of reads using the approach previously described for Arabidopsis phyllosphere microbiome study (Chen et al. 2020). After QIIME2 processing, the filtered representative sequences and ASV tables were used to determine taxonomic abundances and for subsequent statistical analyses in R (see ‘Statistical Analysis and Data Visualisation’ for details).

### 5.4 RNA Extraction, High‐Throughput Sequencing and Data Processing

To extract RNA for gene expression analysis, the complete frozen root systems of plants were pulverized in liquid nitrogen and RNAs were extracted using TRIzol reagent following the manufacturer's protocols (Invitrogen, Carlsbad, CA, USA). RNA quality and integrity were determined using agarose gel electrophoresis and a 2100 Bioanalyzer. High‐quality RNA was treated with DNase (5 U/μL) (TaKaRa, Japan) at 37°C for 30 min, purified using Dynabeads Oligo (dT) 25 (Life Technologies, USA) and used for cDNA library construction and sequencing (Illumina HiSeq, PE 2 × 150 bp) by Shanghai Hanyu Biotech Co. (Shanghai, China). Illumina raw sequencing reads from RNA samples were preprocessed using Trimmomatic (Bolger et al. 2014); reads with a quality score < 20 and reads < 75 bp long were discarded. Clean reads were mapped to the genome of *Triticum aestivum* Chinese Spring (IWGSC RefSeq v1.0, https://wheat‐urgi.versailles.inrae.fr/), and the expression level of each gene mapped to Chinese Spring was calculated by Kallisto software (Bray et al. 2016).

### 5.5 Statistical Analysis and Data Visualisation

For RNA‐seq data, Sleuth R package, which makes use of quantification uncertainty estimates obtained via Kallisto (as described in RNA processing) as for accurate differential analysis of genes (Pimentel et al. 2016), was used to calculate DEGs between sample groups. Hierarchical clustering analysis and visualisation were performed using the ‘Heatmap’ function in R from the ComplexHeatmap package (Gu et al. 2016) and OmicStudio tools at https://www.omicstudio.cn/tool using the DEGs. Genes were clustered based on Euclidean distance and the complete‐linkage method. UpSet plots were generated using the R package UpSetR. Weighted gene co‐expression network analysis (WGCNA) was performed using the R package WGCNA. Hub genes within each co‐expression module (e.g., MEturquoise) were defined as genes exhibiting both strong intramodular connectivity, indicated by absolute module membership (|MM|) > 0.8 [or > 0.9 if stricter], and significant association with the tolerance trait, indicated by absolute gene significance (|GS|) > 0.5. Annotation information for GO enrichment analysis was downloaded from https://wheat‐urgi.versailles.inra.fr/Seq‐Repository/Assemblies. GO enrichment analysis was performed and the results visualised using the R packages ClusterProfiler and enrichplot. KEGG enrichment analysis was performed on the eggNOG‐mapper platform to identify over‐represented biological processes. TBtools software was used to sort the gene functional annotation results. The results of KEGG enrichment analysis were visualised on the OmicShare platform (https://www.omicshare.com/tool). Correlation network analysis between the root‐associated bacterial microbiome and root transcriptome was performed on the Tutools platform (http://www.cloudtutu.com).

For microbiota data analysis, PCoA plots based on the Bray‐Curtis and weighted UniFrac dissimilarities of bacterial and fungal ASVs were used to visualise the effects of *F. graminearum* infection on the wheat root‐associated microbiome and permutational multivariate analysis of variance (PERMANOVA, R‐vegan function Adonis) (Anderson 2001) and analysis of similarities (ANOSIM, R‐vegan function ANOSIM) (Clarke 1993) with 9999 permutations were used to test significant differences between groups based on the Bray‐Curtis and weighted UniFrac dissimilarities. Differences in alpha diversities and taxa with differential relative/absolute abundance between uninoculated and PH‐1‐inoculated samples of different wheat varieties were identified via the non‐parametric Kruskal–Wallis rank test with false discovery rate (FDR) correction (*α* = 0.05) using the Kruskal function in the R package agricolae. Significant taxa with FDR < 0.05 were assigned as taxa with elevated/decreased absolute abundance or enriched/depleted relative abundance in diploid wheats and polyploids before and after inoculation with PH‐1. Data visualisation was performed using the ggplot2 R package. LEfSe (linear discriminant analysis effect size) (Segata et al. 2011) was used to determine the features (differential microbial taxa) most likely to explain differences among wheat varieties with different ploidy levels, between uninoculated and PH‐1‐inoculated samples of different wheat varieties and between disease‐resistant and ‐susceptible wheat varieties. PICRUSt 2.0 (Phylogenetic Investigation of Communities by Reconstruction of Unobserved States) (Douglas et al. 2020) was used to predict the functional abundances of bacteria based only on 16S marker gene sequences. Differential abundance analysis and visualisation of the outputs from PICRUSt2 were performed using the R package ggpicrust2. Functional predictions of fungal microbiota were performed using the FUNGuild database and FUNGuildR package in R. The DESeq2 R package (Love et al. 2014) was used to identify significantly enriched/depleted differential core ASVs. Functional profiles between uninoculated and PH‐1‐inoculated samples of different wheat varieties and between disease‐resistant and ‐susceptible wheat varieties were inferred using PICRUSt2. Gephi software was used to visualise root‐associated microbiome networks. Relative disease spots areas were acquired using ImageJ software.

### 5.6 ND‐FISH Karyotyping

Mitotic chromosome spreads of examined stocks were karyotyped by sequential non‐denaturing fluorescence in situ hybridisation (ND‐FISH) according to the method described previously (Li et al. 2019). Root tip specimens measuring 1–2 cm in length underwent sequential treatment involving exposure to 1.0 MPa nitrous oxide gas for 2 h, fixation in 90% acetic acid solution for 10 min, followed by thorough rinsing with distilled water, with subsequent slide preparation employing the standard air‐drying technique (Li et al. 2019). Custom‐designed oligonucleotide probes, including 5′‐modified oligo‐pTa535, Tamra‐labelled oligo‐(GAA)10, oligo‐pSc119.2 and 6‐FAM‐conjugated oligo‐pTa71, were commercially obtained from Invitrogen (Shanghai, China), with probe sequences adapted from previous publications (Tang et al. 2014; Cui et al. 2018). For sequential two‐round ND‐FISH, slides were washed and rehybridised with another set of probes according to Komuro et al. (2013). ND‐FISH signals were observed and captured using an Olympus BX‐53 epifluorescence microscope equipped with a DP80 microscope digital camera and cellSens Standard 1.8 software (Olympus Corporation, Tokyo, Japan), with final image optimisation performed using Adobe Photoshop CS6 (Adobe Systems Incorporated, San Jose, USA).

## Author Contributions

Conceptualization: X.W., X.H., G.A. and E.W.; methodology: X.H. and X.W.; investigation: X.H., X.W., H.L., J.S., M.J., Z.D., F.N. and Z.C.; writing – original draft: X.H.; writing – review and editing: X.H. and X.W.; funding acquisition: X.W., H.L. and E.W.; resources: H.L., X.Z., K.S., and Y.Z.; supervision: X.W., H.L., Y.Z., G.A. and E.W.

## Conflicts of Interest

The authors declare no conflicts of interest.

## Supporting information


**Figure S1:** Root‐associated bacterial and fungal family‐level compositions among different ploidy‐level wheat varieties.
**Figure S2:** Hierarchical clustering analysis heatmap to compare the expression levels of differentially expressed genes (DEGs with |log_2_FC| > 2 and *q* < 0.05) enriched in response to stress, response to water and defence response between wheat varieties with different disease resistances and their respective controls after PH‐1 inoculation.
**Figure S3:** Hierarchical clustering analysis heatmap to compare the expression levels of differentially expressed genes (DEGs with |log_2_FC| > 2 and *q* < 0.05) enriched in defence response to fungus, defence response to bacterium and response to wounding respectively between wheat varieties with different disease resistances and their respective controls after PH‐1 inoculation.
**Figure S4:** WCGNA of differentially expressed genes (DEGs with |log_2_FC| > 2 and *q* < 0.01) among wheat varieties with different disease resistances before and after PH‐1 inoculation.
**Figure S5:** GO and KEGG pathway enrichment analysis of hub DEGs in three key modules correlated with HG116 and AK58 identified by WCGNA.
**Figure S6:** *F. graminearum* infection altered the diversity of wheat root‐associated microbiomes.
**Figure S7:** *F. graminearum* infection altered the assembly of wheat root endophytic associated microbiomes.
**Figure S8:** *F. graminearum* infection resulted in wheat root‐associated fungal microbiota dysbiosis.
**Figure S9:** The variations of rhizosphere bacterial communities and functions of *F. graminearum*‐tolerant SHW HG116 and its susceptible parents LDN and SY41 following PH‐1 infection.
**Figure S10:** The variations of rhizosphere bacterial communities and functions of *F. graminearum*‐resistant allohexaploid AK58 and susceptible variety CS following PH‐1 infection.
**Figure S11:** The variations of root endophytic bacterial communities and functions of *F. graminearum*‐tolerant SHW HG116 and its susceptible parents LDN and SY41 following PH‐1 infection.
**Figure S12:** The variations of root endophytic bacterial communities and functions of *F. graminearum*‐resistant allohexaploid AK58, and susceptible variety CS following PH‐1 infection.
**Figure S13:** The variations of rhizosphere fungal communities and functions of *F. graminearum*‐tolerant SHW HG116 and its susceptible parents LDN and SY41 following PH‐1 infection.
**Figure S14:** The variations of rhizosphere fungal communities and functions of *F. graminearum*‐resistant allohexaploid AK58 and susceptible variety CS following PH‐1 infection.
**Figure S15:** The variations of root endophytic fungal communities of *F. graminearum*‐tolerant SHW HG116 and its susceptible parents LDN and SY41 following PH‐1 infection.
**Figure S16:** The variations of root endophytic fungal communities of *F. graminearum*‐resistant allohexaploid AK58 and susceptible variety CS following PH‐1infection.
**Figure S17:** Heatmap of functional profiles with significant differences predicted by PICRUSt2 between wheat varieties with different disease resistances and their respective controls.
**Figure S18:** Functional shifts in rhizosphere and root endophytic fungal microbiota across wheat varieties following PH‐1 infection.
**Figure S19:** Comparison of the differential rhizosphere bacterial biomarkers between *F. graminearum*‐tolerant SHW HG116 and other susceptible wheat varieties by LEfSe analysis.
**Figure S20:** Comparison of the differential rhizosphere bacterial biomarkers between *F. graminearum*‐resistant allohexaploid AK58 and other susceptible wheat varieties by LEfSe analysis.
**Figure S21:** Comparison of the differential root endophytic bacterial biomarkers between *F. graminearum*‐tolerant SHW HG116 and other susceptible wheat varieties by LEfSe analysis.
**Figure S22:** Comparison of the differential root endophytic bacterial biomarkers between *F. graminearum*‐resistant allohexaploid AK58 and other susceptible wheat varieties by LEfSe analysis.
**Figure S23:** Comparison of the differential rhizosphere bacteria Core ASVs between *F. graminearum*‐tolerant and ‐resistant hexaploid wheats (HG116 and AK58) and other susceptible wheat varieties based on DESeq2 analysis.
**Figure S24:** Comparison of the differential root endophytic bacteria Core ASVs between *F. graminearum*‐tolerant and ‐resistant hexaploid wheats (HG116 and AK58) and other susceptible wheat varieties based on DESeq2 analysis.
**Figure S25:** Bubble plot of significantly enriched functional profiles inferred by PICRUSt2 in rhizosphere bacterial microbiota of *F. graminearum*‐tolerant and ‐resistant hexaploid wheats (HG116 and AK58) compared to other susceptible wheat varieties following PH‐1 infection.
**Figure S26:** Bubble plot of significantly depleted functional profiles inferred by PICRUSt2 in rhizosphere bacterial microbiota of *F. graminearum*‐tolerant and ‐resistant hexaploid wheats (HG116 and AK58) compared to other susceptible wheat varieties following PH‐1 infection.
**Figure S27:** Bubble plot of significantly enriched functional profiles inferred by PICRUSt2 in root endophytic bacterial microbiota of *F. graminearum*‐tolerant and ‐resistant hexaploid wheats (HG116 and AK58) compared to other susceptible wheat varieties following PH‐1 infection.
**Figure S28:** Bubble plot of significantly depleted functional profiles inferred by PICRUSt2 in root endophytic bacterial microbiota of *F. graminearum*‐tolerant and ‐resistant hexaploid wheats (HG116 and AK58) compared to other susceptible wheat varieties following PH‐1infection.
**Figure S29:** *F. graminearum* infection altered wheat root‐associated fungal microbial networks.
**Figure S30:** The distribution of degree centrality of all subnetworks and betaNTI of bacterial microbiota in the correlation networks between the root‐associated bacterial microbiome and root transcriptome.
**Figure S31:** The comparison of ASV‐gene subnetworks in the correlation networks between the root‐associated bacterial microbiome and root transcriptome between control and inoculated groups.
**Figure S32:** The correlation networks between the root endophytic bacterial microbiome and root transcriptome.
**Figure S33:** The correlation networks between the rhizosphere fungal microbiome and root transcriptome.
**Figure S34:** The distribution of degree centrality of all subnetworks and betaNTI of fungal microbiota in the correlation networks between the root‐associated fungal microbiome and root transcriptome.
**Figure S35:** The comparison of ASV‐gene subnetworks in the correlation networks between the root‐associated fungal microbiome and root transcriptome between control and inoculated groups.
**Figure S36:** The correlation networks between the root endophytic fungal microbiome and root transcriptome.
**Figure S37:** Hierarchical clustering analysis heatmap to compare the expression levels of differentially expressed genes (DEGs with |log_2_FC| > 2 and *q* < 0.05) enriched in stress and defence‐related pathways between HG116 and LDN and SY41 before PH‐1 inoculation.
**Figure S38:** Gene ontology (GO) annotations and Hierarchical clustering analysis of differentially expressed genes (DEGs with |log_2_FC| > 2 and *q* < 0.05) between HG116 and LDN and SY41 after PH‐1 inoculation.

**Table S1:** DEGs among the different ploidy‐level wheat accessions.
**Table S2:** The difference significance test and data of phylum and family‐level relative abundance of root‐associated microbiota among the different ploidy‐level wheat accessions.
**Table S3:** The hub genes in three key modules associated with the *F. graminearum*‐tolerant SHW line HG116 and *F. graminearum*‐resistant variety AK58.
**Table S4:** The difference significance test and data of phylum‐level relative abundance of root‐associated microbial communities between control and inoculated samples of all wheat varieties.
**Table S5:** The discriminating amplicon sequence variants (ASVs) between control and PH‐1 inoculated samples in the root‐associated microbiota of all wheat varieties.
**Table S6:** The difference analysis of MetaCyc pathways of root‐associated bacteria microbiomes between control and PH‐1 inoculated samples of all wheat varieties.
**Table S7:** The discriminating bacterial ASVs in the root‐associated bacterial microbiota between the *F. graminearum*‐tolerant/resistant allohexaploids and the *F. graminearum*‐susceptible wheat varieties following PH‐1 infection.
**Table S8:** The full names of the abbreviations for the descriptions of MetaCyc pathways in Figures S25–S28.
**Table S9:** Bacterial and fungal metadata and sequencing statistics.

## Data Availability

Some of the data obtained in this study are provided in the Tables S1‐S9. The complete data and code that support the findings of this study are available upon request from the corresponding author.
