# Supplementary material for: Synthetic Polyploidisation Enhances Fusarium graminearum Tolerance in Wheat by Reshaping the Transcriptome and Strengthening the Microbiome
Source: Plant Biotechnol J. 2025 Aug 10;23(11):5252–78. doi: 10.1111/pbi.70295 (PMC12576463; doi:10.1111/pbi.70295)
Supplement: Supplementary file 1 — Figure S1: Root‐associated bacterial and fungal family‐level compositions among different ploidy‐level wheat varieties. Figure S2: Hierarchical clustering analysis heatmap to compare the expression levels of differentially expressed genes (DEGs with |log2FC| > 2 and q < 0.05) enriched in response to stress, response to water and defence response between wheat varieties with different disease resistances and their respective controls after PH‐1 inoculation. Figure S3: Hierarchical clustering analysis heatmap to compare the expression levels of differentially expressed genes (DEGs with |log2FC| > 2 and q < 0.05) enriched in defence response to fungus, defence response to bacterium and response to wounding respectively between wheat varieties with different disease resistances and their respective controls after PH‐1 inoculation. Figure S4: WCGNA of differentially expressed genes (DEGs with |log2FC| > 2 and q < 0.01) among wheat varieties with different disease resistances before and after PH‐1 inoculation. Figure S5: GO and KEGG pathway enrichment analysis of hub DEGs in three key modules correlated with HG116 and AK58 identified by WCGNA. Figure S6: F. graminearum infection altered the diversity of wheat root‐associated microbiomes. Figure S7: F. graminearum infection altered the assembly of wheat root endophytic associated microbiomes. Figure S8: F. graminearum infection resulted in wheat root‐associated fungal microbiota dysbiosis. Figure S9: The variations of rhizosphere bacterial communities and functions of F. graminearum‐tolerant SHW HG116 and its susceptible parents LDN and SY41 following PH‐1 infection. Figure S10: The variations of rhizosphere bacterial communities and functions of F. graminearum‐resistant allohexaploid AK58 and susceptible variety CS following PH‐1 infection. Figure S11: The variations of root endophytic bacterial communities and functions of F. graminearum‐tolerant SHW HG116 and its susceptible parents LDN and SY41 following PH‐1 infection [file PBI-23-5252-s001.pdf]

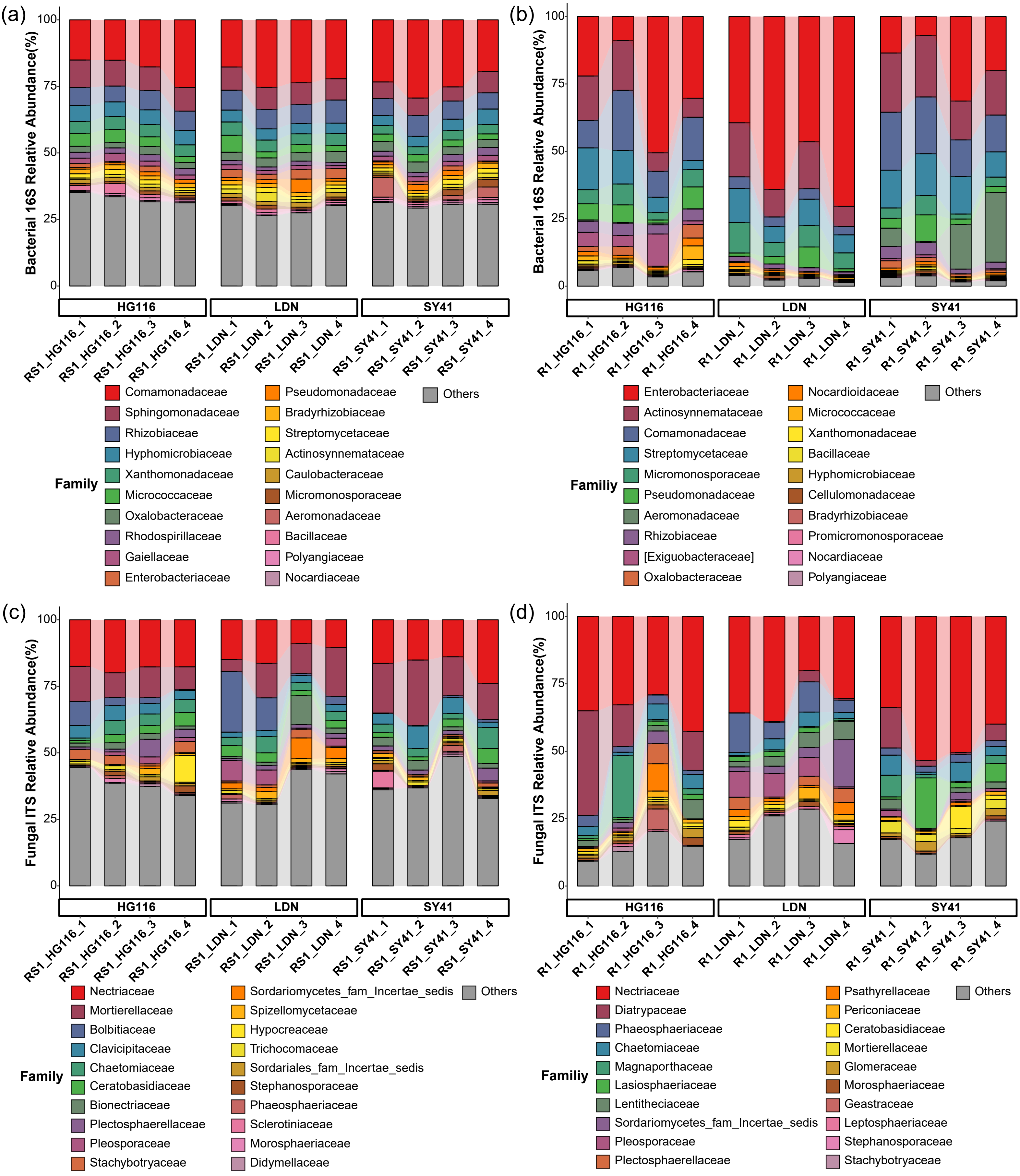

Figure S1 Root-associated bacterial and fungal family-level compositions among different ploidy-level wheat varieties.
(a-b) Comparing of the distribution of top 20 bacterial families of the rhizosphere (a) and root endophytic (b) bacterial microbiota among HG116, LDN and SY41 using 16S rRNA genes. (c-d) Comparing of the distribution of top 20 fungal families of the rhizosphere (c) and root endophytic (d) fungal microbiota across HG116, LDN and SY41 using ITS rRNA genes. bars. Major contributing families were displayed in different colors and minor contributing families were grouped and displayed in grey.

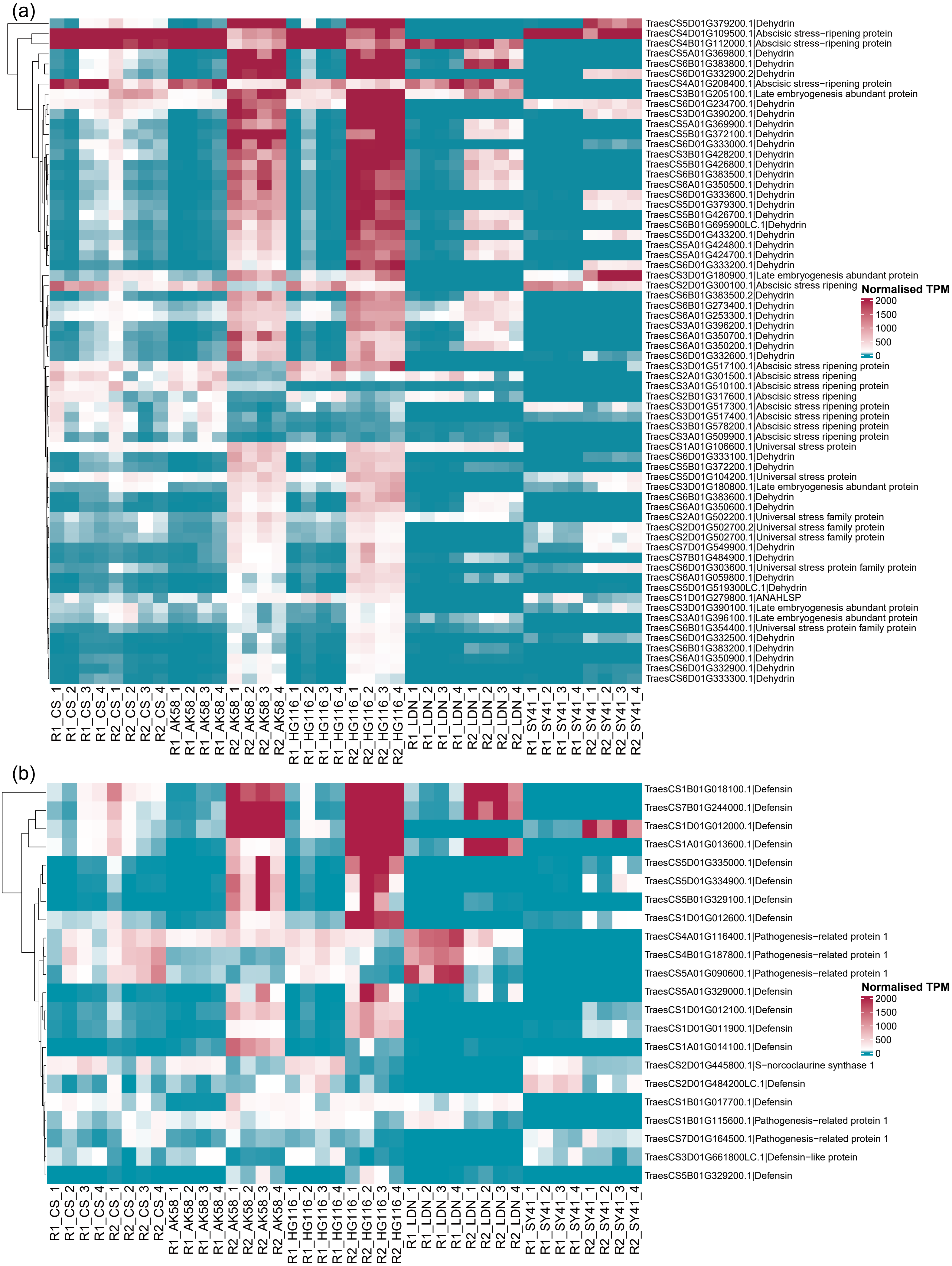

Figure S2 Hierarchical clustering analysis heatmap to compare the expression levels of differentially expressed genes (DEGs with $|\log_2 FC| > 2$ and $q < 0.05$) enriched in response to stress and response to water (a), and response (b) between wheat varieties with different disease resistances and their respective controls after PH-1 inoculation. The color indicated high or low expression levels for DEGs. ANAHLSP: Adenine nucleotide alpha hydrolases-like superfamily protein.

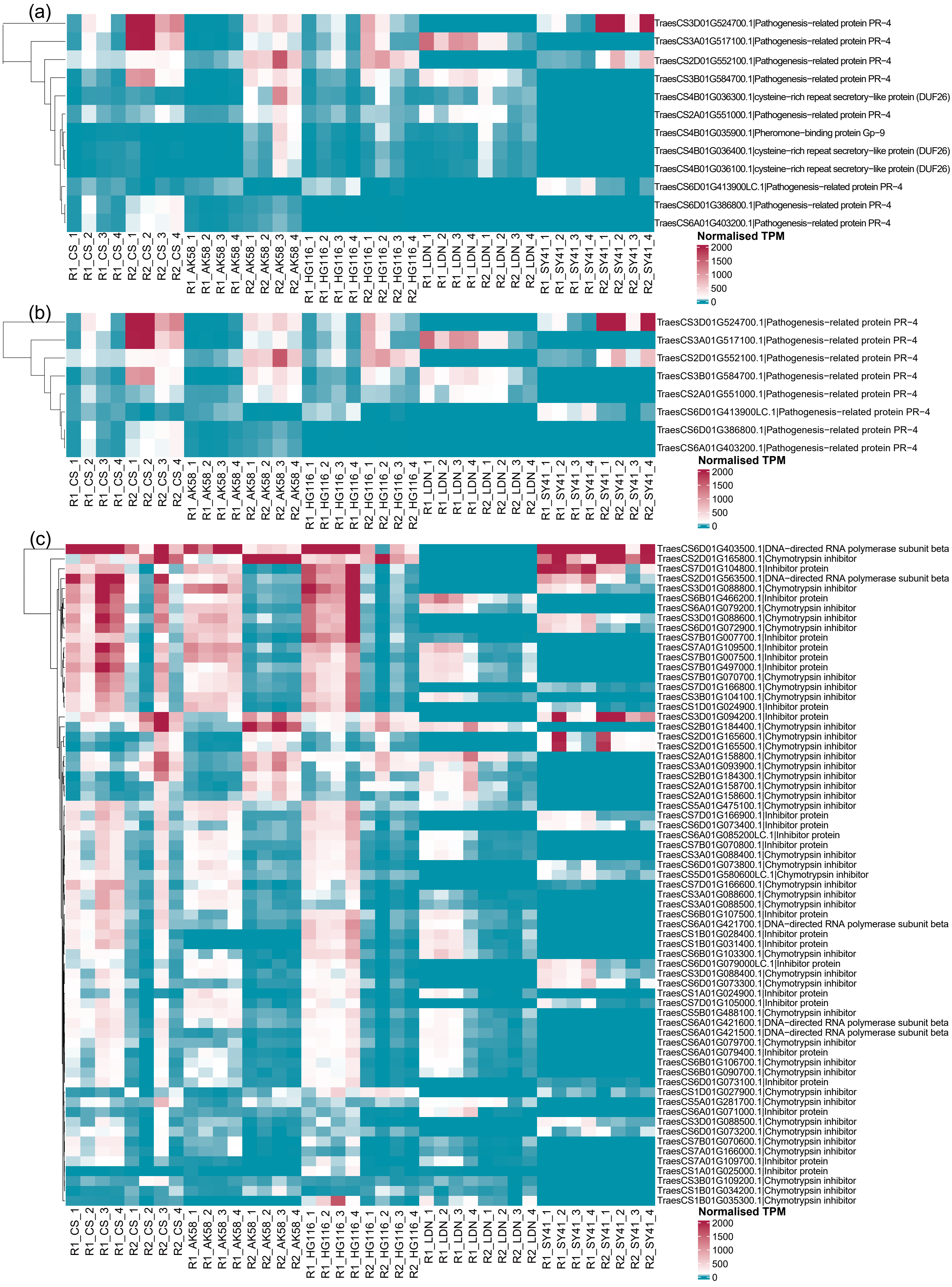

Figure S3 Hierarchical clustering analysis heatmap to compare the expression levels of differentially expressed genes (DEGs with $|\log_2 FC| > 2$ and $q < 0.05$), enriched in defence response to fungus (a), defence response to bacterium (b) and response to wounding (c) respectively between wheat varieties with different disease resistances and their respective controls after PH-1 inoculation. The color indicated high or low expression levels for hub DEGs.

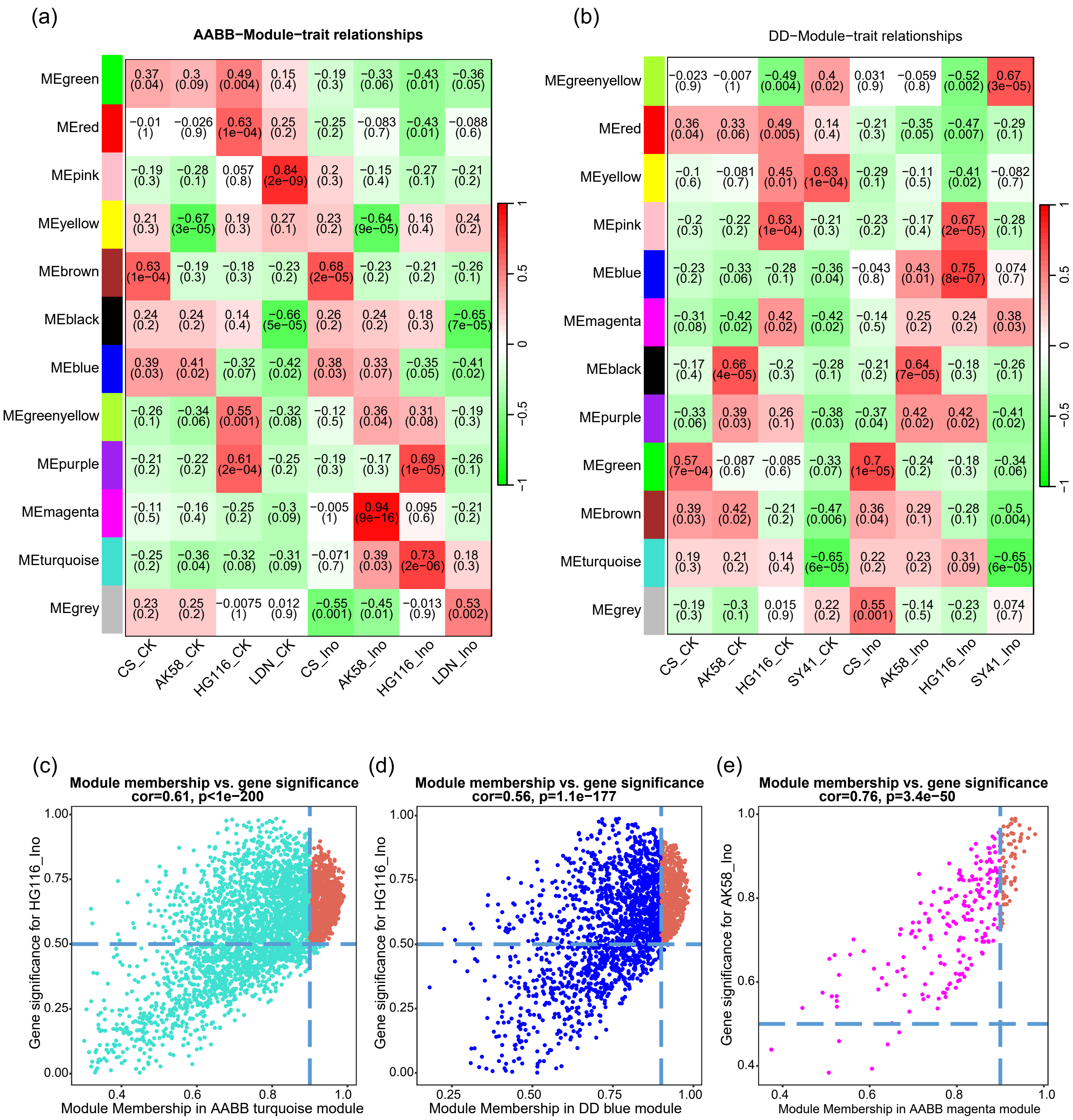

Figure S4 WCGNA of differentially expressed genes (DEGs with $|\log_2 FC| > 2$ and $q < 0.01$) among wheat varieties with different disease resistances before and after PH-1 inoculation. (a-b) The heatmap of relationships between modules and resistance traits. (a) Module-trait associations on AABB subgenomes. (b) Module-trait associations on D subgenome. Each column corresponds to a trait, and each row corresponds to a ME. The number in the rectangle is the correlation coefficient, and the number in brackets is the corresponding pvalue. The table is color-coded by correlation based on the color legend. (c-e) Scatterplots of GS for HG116 and AK58 (y-axis) versus MM (x-axis) in following three modules: AABB-MEturquoise module (c) and DD-MEblue module (d) accosiated with HG116_Ino, and AABB-MEmagenta module accosiated with AK58_Ino (e). Light steel blue lines indicated the screening criteria, set as |GS|>0.5 and |MM|>0.9 in this study. Warm coral-red dots located in the top right corner of each graph represented the hub genes in each module.

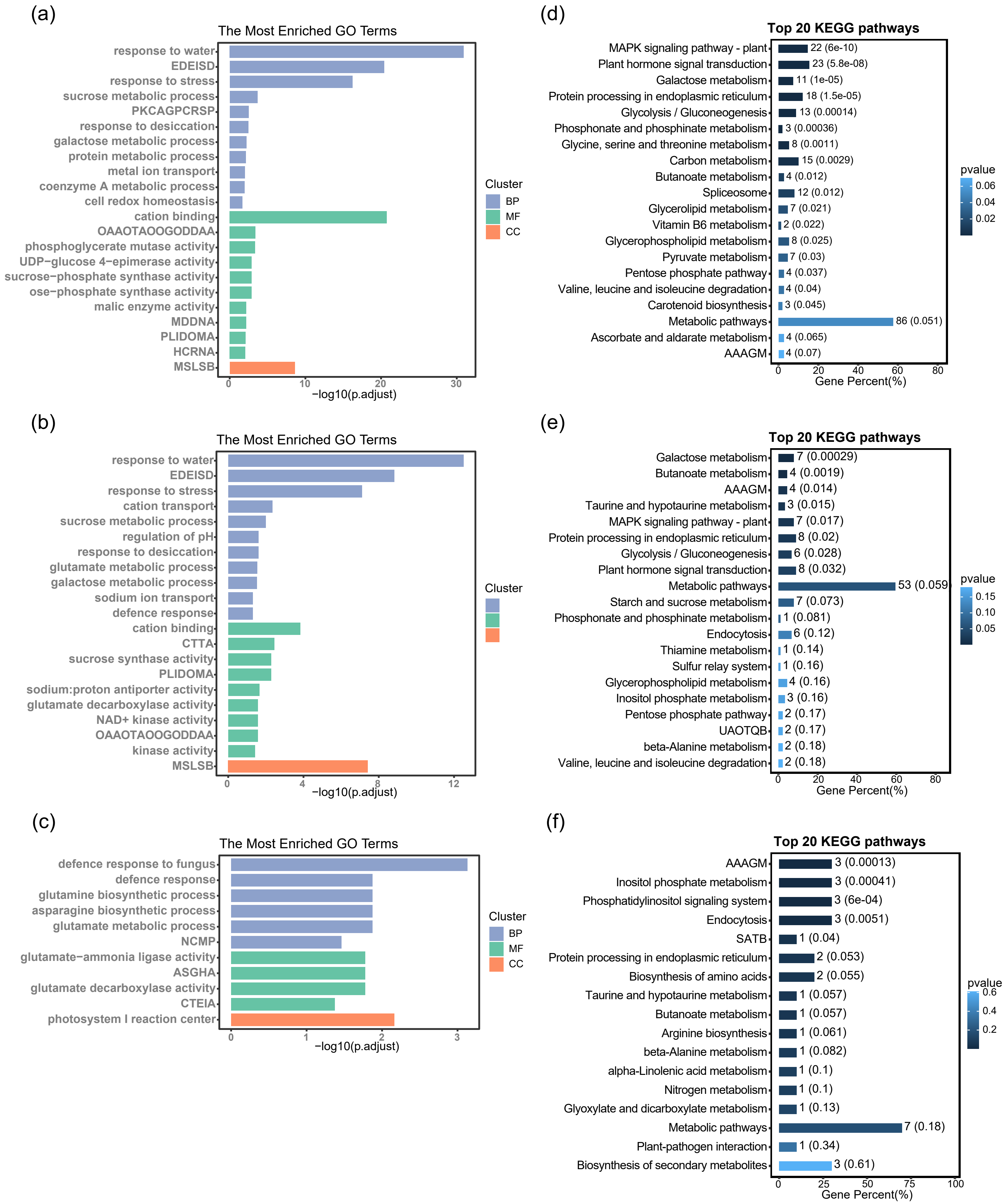

Figure S5 GO and KEGG pathway enrichment analysis of hub DEGs in three key modules correlated with HG116 and AK58 identified by WCGNA.
(a-c) GO enrichment analysis of hub DEGs in AABB-MEturquoise module (a) and DD-MEblue module (b) correlated with HG116_Ino, and AABB-MEmagenta module (c) correlated with AK58_Ino. (d-e) KEGG pathway enrichment analysis of hub DEGs in AABB-MEturquoise module (d) and DD-MEblue module (e) correlated with HG116_Ino, and AABB-MEmagenta module (f) correlated with AK58_Ino. EDEISD: embryo development ending in seed dormancy; PKCAGPCRSP: protein kinase C-activating G-protein coupled receptor signaling pathway; OAAOTAOOGODDAA: oxidoreductase activity, acting on the aldehyde or oxo group of donors, disulfide as acceptor; MDDNA: malate dehydrogenase (decarboxylating) (NAD+) activity; PLIDOMA: protein-L-isoaspartate (D-aspartate) O-methyltransferase activity; HCRNA: hydroxymethylglutaryl-CoA reductase (NADPH) activity; MSLSB: monolayer-surrounded lipid storage body; CTTA: cation transmembrane transporter activity; NCMP: nitrogen compound metabolic process; ASGHA: asparagine synthase (glutamine-hydrolyzing) activity; CTEIA: cysteine-type endopeptidase inhibitor activity; AAAGM: Alanine, aspartate and glutamate metabolism; UAOTQB: Ubiquinone and other terpenoid-quinone biosynthesis; SATB: Sesquiterpenoid and triterpenoid biosynthesis.

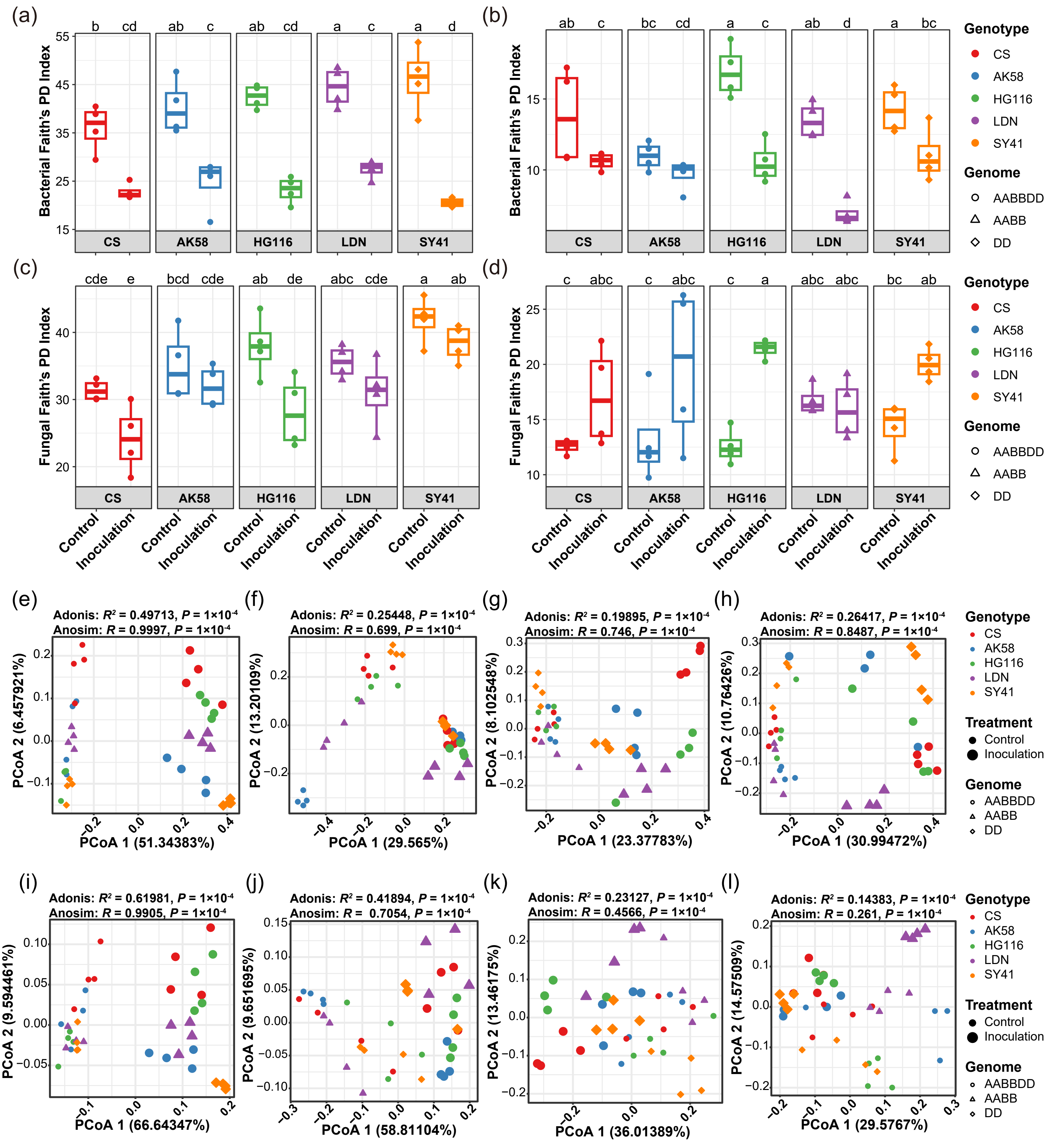

Figure S6 *F. graminearum* infection altered the diversity of wheat root-associated microbiomes.
(a-d) Comparison of Faith's PD index for rhizosphere bacteria (a) and fungi (c), and root endophytic bacteria (b) and fungi (d) respectively, between wheat varieties with different disease-resistances and their respective controls. Post hoc test results, indicated by letters at the top, show that sample groups with the same letter are indistinguishable at 95% confidence. Each comparison includes 4 biological replicates (n = 4). (e-h) Principal coordinate analysis (PCoA) of Bray-Curtis distances in the rhizosphere bacteria (e) and fungi (g) microbiota, and root endophytic bacteria (f) and fungi (h) microbiota, respectively, following infection.(i-l) Principal coordinate analysis (PCoA) of weighted UniFrac distances distances in the rhizosphere bacteria (i) and fungi (k) microbiota, and root endophytic bacteria (j) and fungi (l) microbiota, respectively, following infection. From (a) to (l), the color and shape of each point represented the wheat variety (genotype) and genome, respectively. From (a) to (d), post hoc test was indicated by letters at the top, sample groups with the same letter are indistinguishable at 95% confidence. n = 4 biological replicates. From (e) to (l), the test results of Adonis (PERMANOVA, permutational MANOVA) and Anosim (Analysis of Similarities) were placed on the top of each graph. The size of points represented the treatment, small size represented control and big size represented inoculation.

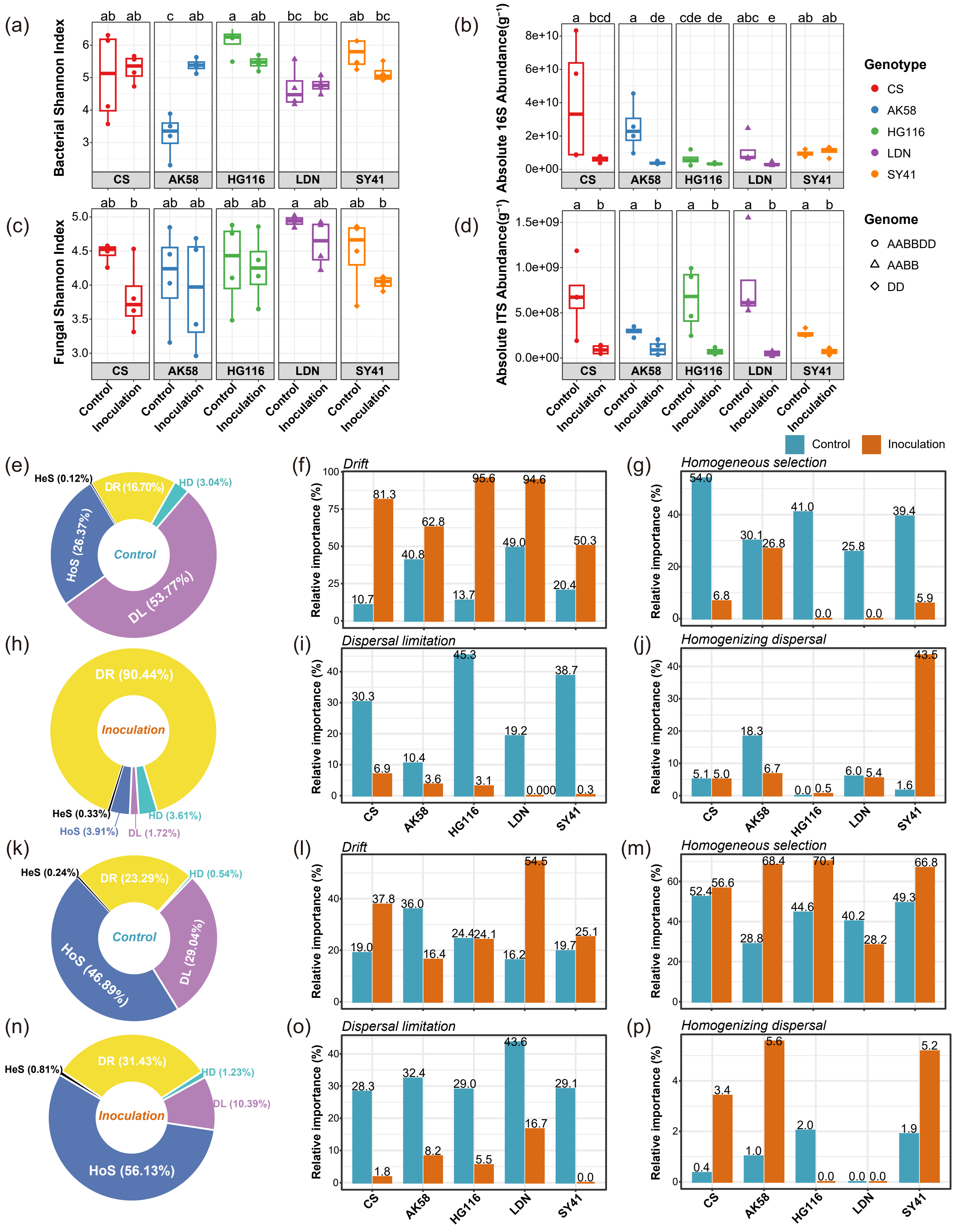

Figure S7 *F. graminearum* infection altered the assembly of wheat root endophytic associated microbiomes.
(a-d) Comparison of the bacterial Shannon index (a) and absolute abundance (b), the fungal Shannon index (c) and absolute abundance (d) between wheat varieties with different disease-resistances and their respective controls. (e-p) The relative importance of different ecological processes in root endophytic bacterial (e-j) and fungal (k-p) community assembly was estimated by iCAMP under control (e, k) and under inoculation (h, n). Specifically, the relative importanceof Drift (f, l), Homogeneous selection (g, m), Dispersal limitation (i, o) and Homogenizing dispersal (j, p) in root endophytic (bacterial, fungal) community assembly of each wheat variety under control (aqua bar) and inoculation (orange bar). From (a) to (d), the color and shape of each point represented the wheat variety (genotype) and genome, respectively. Post hoc test was indicated by letters at the top, sample groups with the same letter are indistinguishable at 95% confidence. n = 4 biological replicates. For (f), (g), (i), (j), (l), (m), (o) and (p), the digits on the top of each bar were the relative importance of each ecological process in governing the turnovers in rhizosphere bacterial and fungal community assembly of each wheat variety.

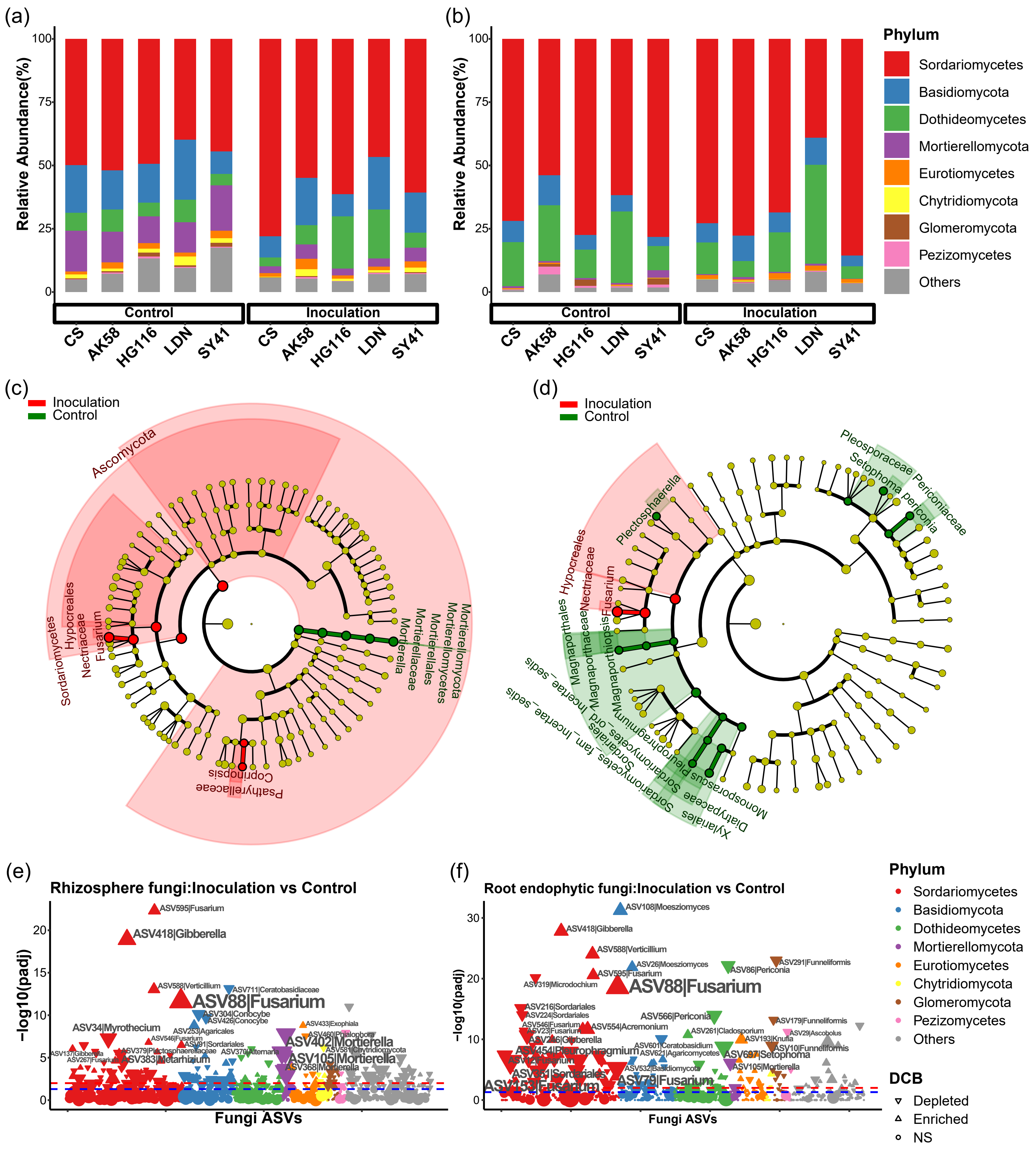

Figure S8 *F. graminearum* infection resulted in wheat root-associated fungal microbiota dysbiosis.
(a-b) Comparison of the phylum-level distribution of the rhizosphere (a) and root endophytic (b) fungal microbiota of wheat varieties with different disease-resistances under control and inoculation. Average relative abundance of 4 biological replicates were displayed in separate stacked bars. (c-d) LDA effect size taxonomic cladogram overall comparing the rhizosphere (c) and root endophytic (d) fungal microbiota between control and inoculated groups of all wheat varieties. Significantly discriminant bacterial taxon nodes were colored and branch areas were shaded according to the highest-ranked variety for that taxon. If the taxon was not significantly differentially represented between sample groups, the corresponding node was colored yellow. (e-f) Manhattan plots showing the overall variations of Core ASVs in rhizosphere (e) and root endophytica (f) fungal microbiota of all wheat varieties following infection. Core ASVs that were significantly enriched were depicted as vertical triangles, that were significantly depleted were depicted as inverted triangles, otherwise depicted as circles. The red and blue dashed line corresponded to the FDR-corrected pvalue of 0.01 and 0.05, respectively. The color of each point represented the phylum-level taxonomic affiliation of the Core ASVs, and the size corresponded to the baseMean of the Core ASVs.

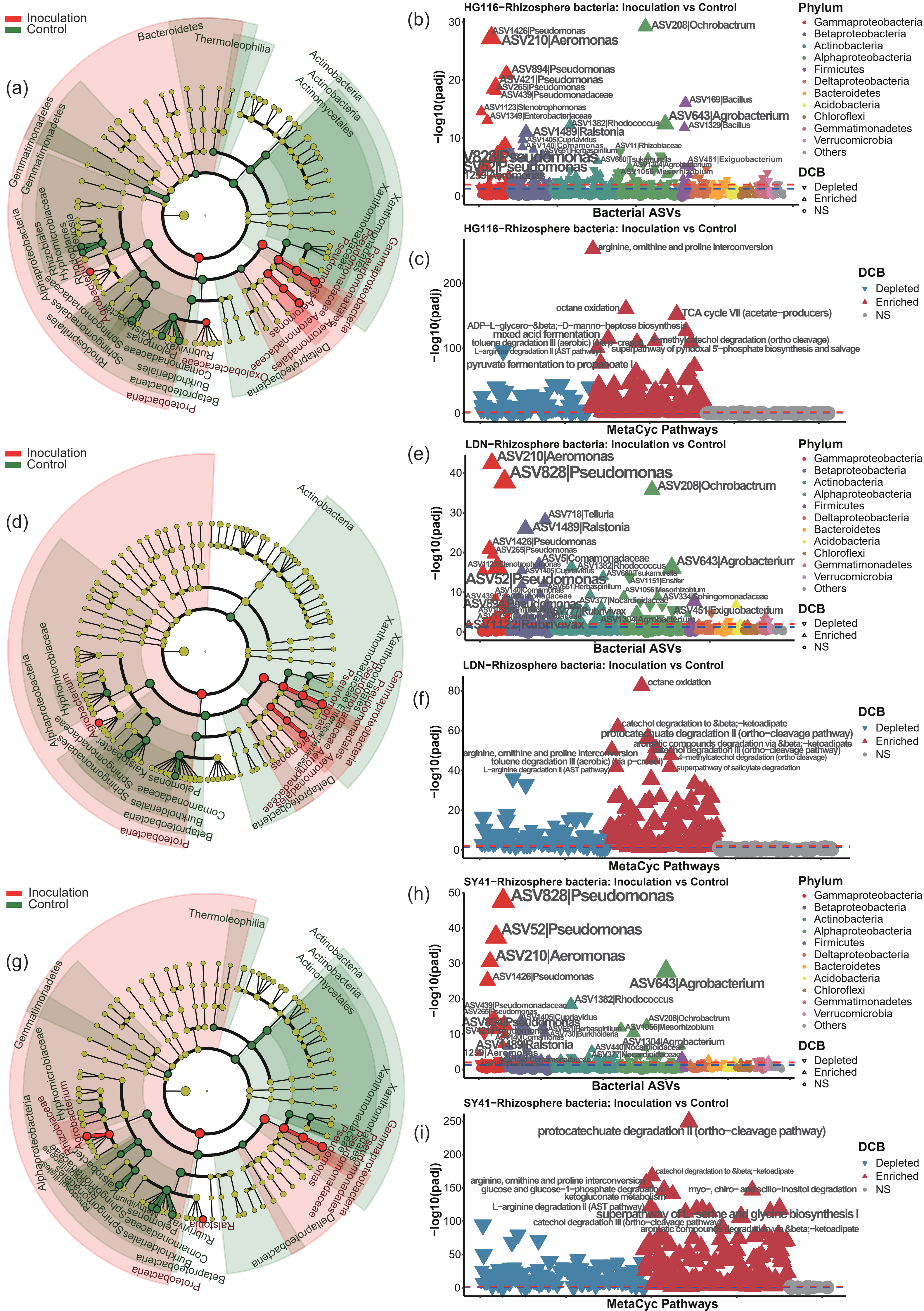

Inoculation
Control
(a)
Bacteroidetes
Thermoleophilia
Actinobacteria
Actinobacteria
Actinomycetales
Gemmatimonadetes
Gemmatimonadetes
Xanthomonadales
Xanthomonadaceae
Gammaproteobacteria
Pseudomonadales
Pseudomonadaceae
Aeromonadaceae
Aeromonadales
Deltaproteobacteria
Oxalobacteraceae
Comamonadaceae
Burkholderiales
Betaproteobacteria
Proteobacteria
Rubrivivax
Polaromonas
Rhodospirillales
Sphingomonadales
Sphingomonadaceae
Alphaproteobacteria
Rhizobiales
Hyphomicrobiaceae
Hyphomonas
(b)
HG116−Rhizosphere bacteria: Inoculation vs Control
−log10(padj)
Bacterial ASVs
ASV1426|Pseudomonas
ASV210|Aeromonas
ASV208|Ochrobactrum
ASV894|Pseudomonas
ASV421|Pseudomonas
ASV265|Pseudomonas
ASV439|Pseudomonadaceae
ASV169|Bacillus
ASV1123|Stenotrophomonas
ASV1349|Enterobacteriaceae
ASV1382|Rhodococcus
ASV643|Agrobacterium
ASV1329|Bacillus
ASV1489|Ralstonia
ASV11|Rhizobiaceae
ASV451|Exiguobacterium
Phylum
Gammaproteobacteria
Betaproteobacteria
Actinobacteria
Alphaproteobacteria
Firmicutes
Deltaproteobacteria
Bacteroidetes
Acidobacteria
Chloroflexi
Gemmatimonadetes
Verrucomicrobia
Others
DCB
Depleted
Enriched
NS
(c)
HG116−Rhizosphere bacteria: Inoculation vs Control
−log10(padj)
MetaCyc Pathways
arginine, ornithine and proline interconversion
octane oxidation
TCA cycle VII (acetate−producers)
mixed acid fermentation
superpathway of pyridoxal 5'−phosphate biosynthesis and salvage
pyruvate fermentation to propanoate I
DCB
Depleted
Enriched
NS
(d)
Inoculation
Control
Actinobacteria
Xanthomonadales
Xanthomonadaceae
Gammaproteobacteria
Alphaproteobacteria
Hyphomicrobiaceae
Hyphomonas
Sphingomonadales
Sphingomonadaceae
Comamonadaceae
Burkholderiales
Betaproteobacteria
Proteobacteria
Deltaproteobacteria
(e)
LDN−Rhizosphere bacteria: Inoculation vs Control
−log10(padj)
Bacterial ASVs
ASV210|Aeromonas
ASV828|Pseudomonas
ASV208|Ochrobactrum
ASV718|Telluria
ASV1489|Ralstonia
ASV1426|Pseudomonas
ASV265|Pseudomonas
ASV5|Comamonadaceae
ASV643|Agrobacterium
ASV52|Pseudomonas
ASV894|Pseudomonas
ASV1151|Ensifer
ASV1056|Mesorhizobium
ASV334|Sphingomonadaceae
ASV451|Exiguobacterium
ASV1304|Agrobacterium
ASV1422|Rubrivivax
Phylum
Gammaproteobacteria
Betaproteobacteria
Actinobacteria
Alphaproteobacteria
Firmicutes
Deltaproteobacteria
Bacteroidetes
Acidobacteria
Chloroflexi
Gemmatimonadetes
Verrucomicrobia
Others
DCB
Depleted
Enriched
NS
(f)
LDN−Rhizosphere bacteria: Inoculation vs Control
−log10(padj)
MetaCyc Pathways
octane oxidation
catechol degradation to β−ketoadipate
protocatechuate degradation II (ortho−cleavage pathway)
arginine, ornithine and proline interconversion
toluene degradation III (aerobic) (via p−cresol)
L−arginine degradation II (AST pathway)
superpathway of salicylate degradation
DCB
Depleted
Enriched
NS
(g)
Inoculation
Control
Thermoleophilia
Actinobacteria
Actinobacteria
Actinomycetales
Gemmatimonadetes
Xanthomonadales
Xanthomonadaceae
Gammaproteobacteria
Pseudomonadales
Deltaproteobacteria
Alphaproteobacteria
Hyphomicrobiaceae
Hyphomicrobium
Ralstonia
Rubrivivax
Comamonadaceae
Burkholderiales
Betaproteobacteria
Proteobacteria
(h)
SY41−Rhizosphere bacteria: Inoculation vs Control
−log10(padj)
Bacterial ASVs
ASV828|Pseudomonas
ASV52|Pseudomonas
ASV210|Aeromonas
ASV1426|Pseudomonas
ASV643|Agrobacterium
ASV1382|Rhodococcus
ASV439|Pseudomonadaceae
ASV208|Ochrobactrum
ASV1056|Mesorhizobium
ASV1489|Ralstonia
ASV1304|Agrobacterium
Phylum
Gammaproteobacteria
Betaproteobacteria
Actinobacteria
Alphaproteobacteria
Firmicutes
Deltaproteobacteria
Bacteroidetes
Acidobacteria
Chloroflexi
Gemmatimonadetes
Verrucomicrobia
Others
DCB
Depleted
Enriched
NS
(i)
SY41−Rhizosphere bacteria: Inoculation vs Control
−log10(padj)
MetaCyc Pathways
protocatechuate degradation II (ortho−cleavage pathway)
catechol degradation to β−ketoadipate
arginine, ornithine and proline interconversion
glucose and glucose−1−phosphate degradation
ketogluconate metabolism
myo−, chiro− and scillo−inositol degradation
L−arginine degradation II (AST pathway)
superpathway of L−serine and glycine biosynthesis I
catechol degradation III (ortho−cleavage pathway)
aromatic compounds degradation via β−ketoadipate
DCB
Depleted
Enriched
NS

Figure S9 The variations of rhizosphere bacterial communities and functions of *F. graminearum*-tolerant SHW HG116 and its susceptible parents LDN and SY41 following PH-1 infection.

(a, d and g) LDA effect size taxonomic cladogram comparing the rhizosphere bacterial microbiota between control and inoculated samples of HG116 (a), LDN (d) and SY41(g), respectively. Significantly discriminant bacterial taxon nodes were colored and branch areas were shaded according to the highest-ranked variety for that taxon. If the taxon was not significantly differentially represented between sample groups, the corresponding node was colored yellow. (b, e and h) Manhattan plots showing the variations of Core ASVs in rhizosphere bacterial microbiota of HG116 (b), LDN (e) and SY41(h) following *F. graminearum* infection. Core ASVs that were significantly enriched were depicted as vertical triangles, that were significantly depleted were depicted as inverted triangles, otherwise depicted as circles. The red and blue dashed line corresponded to the FDR-corrected pvalue of 0.01 and 0.05, respectively. The color of each point represented the phylum-level taxonomic affiliation of the Core ASVs, and the size corresponded to the baseMean of the Core ASVs. (c, f and i) Manhattan plots showing the shifts of MetaCyc pathways in rhizosphere bacterial microbiota of HG116 (c), LDN (f) and SY41(i) following *F. graminearum* infection. Predicted bacterial functions using PICRUSt2 that were significantly enhanced were depicted as burgundy vertical triangles, that were significantly weakened were depicted as steel blue inverted triangles, otherwise depicted as grey circles. The red and blue dashed line corresponded to the FDR-corrected pvalue of 0.01 and 0.05, respectively. Each point represented each MetaCyc pathway, and the size of each point corresponded to the baseMean of functional abundance of MetaCyc pathway.

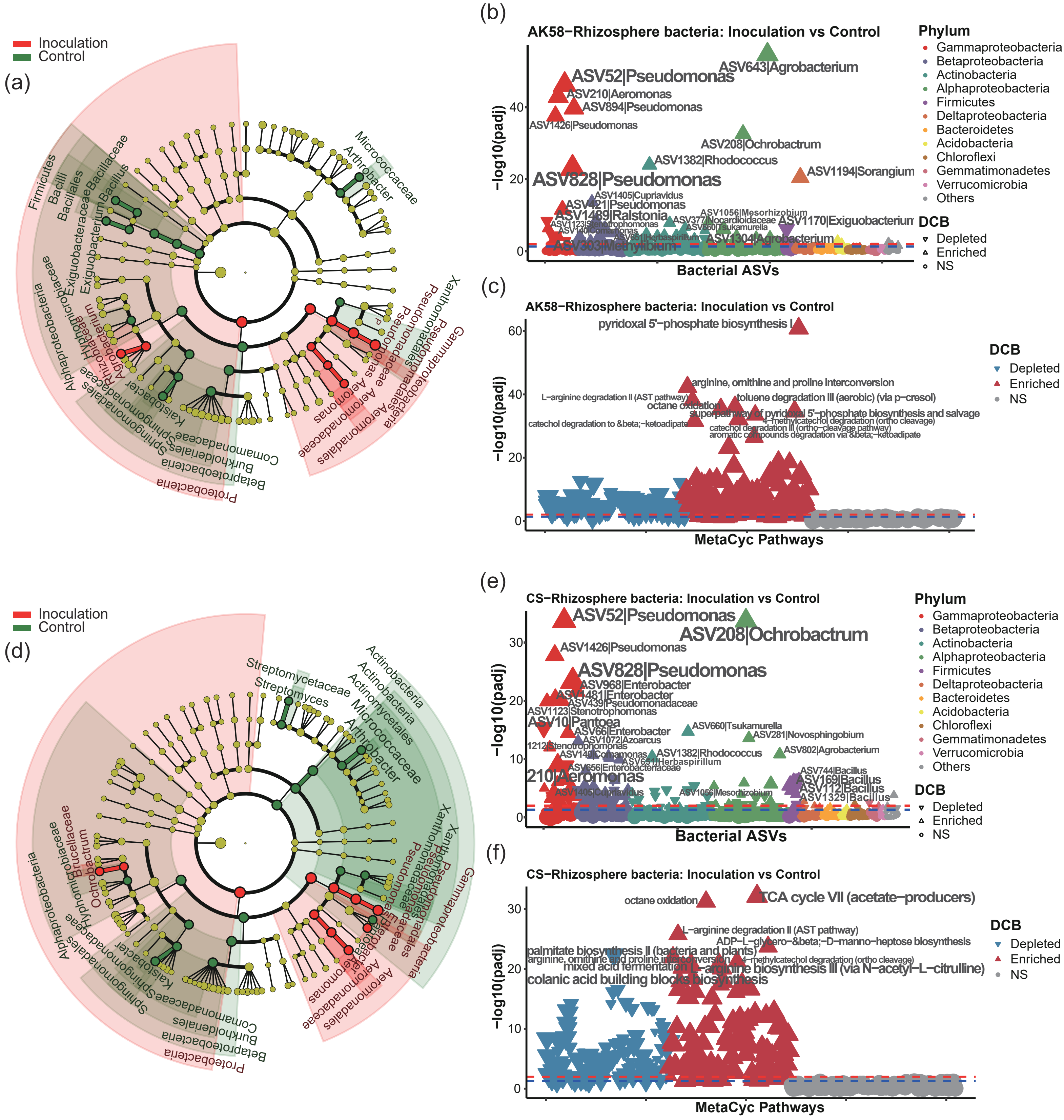

Figure S10 The variations of rhizosphere bacterial communities and functions of *F. graminearum*-resistant allohexaploid AK58 and susceptible variety CS following PH-1 infection.

(a and d) LDA effect size taxonomic cladogram comparing the rhizosphere bacterial microbiota between control and inoculated samples of AK58 (a) and CS (d), respectively. Significantly discriminant bacterial taxon nodes were colored and branch areas were shaded according to the highest-ranked variety for that taxon. If the taxon was not significantly differentially represented between sample groups, the corresponding node was colored yellow. (b and e) Manhattan plots showing the variations of Core ASVs in rhizosphere bacterial microbiota of AK58 (b) and CS (e) following *F. graminearum* infection. Core ASVs that were significantly enriched were depicted as vertical triangles, that were significantly depleted were depicted as inverted triangles, otherwise depicted as circles. The red and blue dashed line corresponded to the FDR-corrected pvalue of 0.01 and 0.05, respectively. The color of each point represented the phylum-level taxonomic affiliation of the Core ASVs, and the size corresponded to the baseMean of the Core ASVs. (c and f) Manhattan plots showing the shifts of MetaCyc pathways in rhizosphere bacterial microbiota of AK58 (c) and CS (f) following *F. graminearum* infection. Predicted bacterial functions using PICRUSt2 that were significantly enhanced were depicted as burgundy vertical triangles, that were significantly weakened were depicted as steelblue inverted triangles, otherwise depicted as grey circles. The red and blue dashed line corresponded to the FDR-corrected pvalue of 0.01 and 0.05, respectively. Each point represented each MetaCyc pathway, and the size of each point corresponded to the baseMean of functional abundance of MetaCyc pathway.

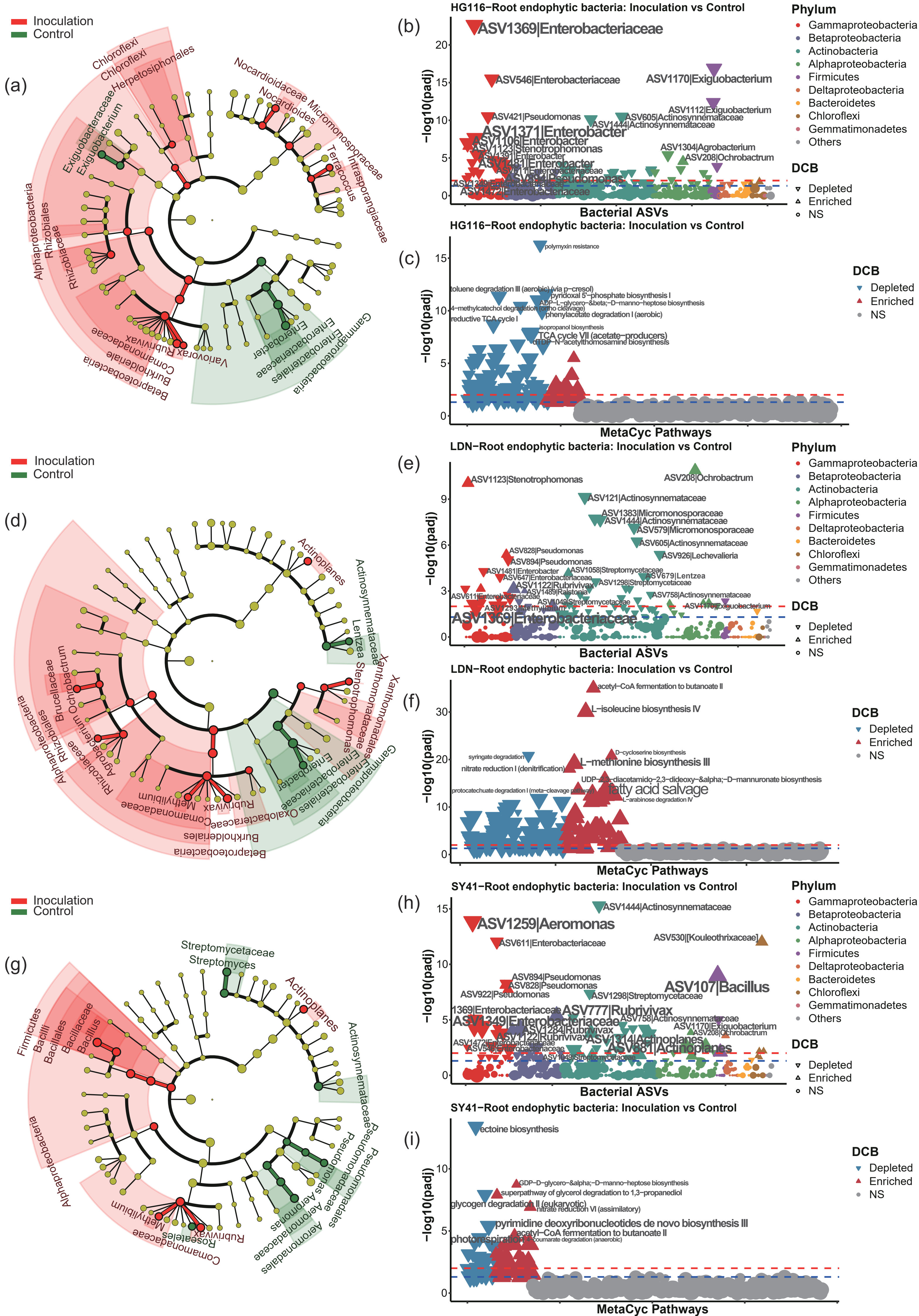
(a)
Inoculation
Control
Chloroflexi
Chloroflexi
Herpetosiphonales
Nocardioidaceae
Nocardioides
Micromonosporaceae
Terracoccus
Intrasporangiaceae
Exiguobacteraceae
Exiguobacterium
Alphaproteobacteria
Rhizobiales
Rhizobiaceae
Rubrivivax
Comamonadaceae
Burkholderiales
Betaproteobacteria
Variovorax
Enterobacter
Enterobacteriaceae
Enterobacteriales
Gammaproteobacteria
(b)
HG116−Root endophytic bacteria: Inoculation vs Control
−log10(padj)
Bacterial ASVs
ASV1369|Enterobacteriaceae
ASV546|Enterobacteriaceae
ASV1170|Exiguobacterium
ASV1112|Exiguobacterium
ASV421|Pseudomonas
ASV605|Actinosynnemataceae
ASV1444|Actinosynnemataceae
ASV1371|Enterobacter
ASV1106|Enterobacter
ASV1123|Stenotrophomonas
ASV1304|Agrobacterium
ASV208|Ochrobactrum
ASV1472|Enterobacteriaceae
Phylum
Gammaproteobacteria
Betaproteobacteria
Actinobacteria
Alphaproteobacteria
Firmicutes
Deltaproteobacteria
Bacteroidetes
Chloroflexi
Gemmatimonadetes
Others
DCB
Depleted
Enriched
NS
(c)
HG116−Root endophytic bacteria: Inoculation vs Control
−log10(padj)
MetaCyc Pathways
polymyxin resistance
toluene degradation III (aerobic) (via p−cresol)
pyridoxal 5'−phosphate biosynthesis I
ADP−L−glycero−β−D−manno−heptose biosynthesis
4−methylcatechol degradation (ortho cleavage)
reductive TCA cycle I
phenylacetate degradation I (aerobic)
isopropanol biosynthesis
TCA cycle VII (acetate−producers)
dTDP−N−acetylthomosamine biosynthesis
DCB
Depleted
Enriched
NS
(d)
Inoculation
Control
Actinoplanes
Actinosynnemataceae
Lentzea
Xanthomonadales
Xanthomonadaceae
Stenotrophomonas
Enterobacteriaceae
Enterobacteriales
Gammaproteobacteria
Enterobacter
Oxalobacteraceae
Rubrivivax
Methylibium
Comamonadaceae
Burkholderiales
Betaproteobacteria
Alphaproteobacteria
Rhizobiales
Brucellaceae
Ochrobactrum
Agrobacterium
Rhizobiaceae
(e)
LDN−Root endophytic bacteria: Inoculation vs Control
−log10(padj)
Bacterial ASVs
ASV1123|Stenotrophomonas
ASV208|Ochrobactrum
ASV121|Actinosynnemataceae
ASV1383|Micromonosporaceae
ASV1444|Actinosynnemataceae
ASV579|Micromonosporaceae
ASV605|Actinosynnemataceae
ASV828|Pseudomonas
ASV926|Lechevalieria
ASV894|Pseudomonas
ASV1481|Enterobacter
ASV1058|Streptomycetaceae
ASV647|Enterobacteriaceae
ASV679|Lentzea
ASV1122|Rubrivivax
ASV1298|Streptomycetaceae
ASV1489|Ralstonia
ASV758|Actinosynnemataceae
ASV611|Enterobacteriaceae
ASV1049|Streptomycetaceae
ASV1170|Exiguobacterium
ASV1369|Enterobacteriaceae
Phylum
Gammaproteobacteria
Betaproteobacteria
Actinobacteria
Alphaproteobacteria
Firmicutes
Deltaproteobacteria
Bacteroidetes
Chloroflexi
Gemmatimonadetes
Others
DCB
Depleted
Enriched
NS
(f)
LDN−Root endophytic bacteria: Inoculation vs Control
−log10(padj)
MetaCyc Pathways
acetyl−CoA fermentation to butanoate II
L−isoleucine biosynthesis IV
D−cycloserine biosynthesis
syringate degradation
nitrate reduction I (denitrification)
L−methionine biosynthesis III
UDP−2,3−diacetamido−2,3−dideoxy−α−D−mannuronate biosynthesis
protocatechuate degradation I (meta−cleavage pathway)
fatty acid salvage
L−arabinose degradation IV
DCB
Depleted
Enriched
NS
(g)
Inoculation
Control
Streptomycetaceae
Streptomyces
Actinoplanes
Actinosynnemataceae
Firmicutes
Bacilli
Bacillales
Bacillaceae
Bacillus
Alphaproteobacteria
Pseudomonas
Pseudomonadaceae
Pseudomonadales
Aeromonas
Aeromonadaceae
Aeromonadales
Rubrivivax
Methylibium
Roseateles
Comamonadaceae
(h)
SY41−Root endophytic bacteria: Inoculation vs Control
−log10(padj)
Bacterial ASVs
ASV1444|Actinosynnemataceae
ASV1259|Aeromonas
ASV611|Enterobacteriaceae
ASV530|[Kouleothrixaceae]
ASV894|Pseudomonas
ASV828|Pseudomonas
ASV107|Bacillus
ASV922|Pseudomonas
ASV1298|Streptomycetaceae
ASV777|Rubrivivax
ASV1349|Enterobacteriaceae
ASV758|Actinosynnemataceae
ASV1170|Exiguobacterium
ASV208|Ochrobactrum
ASV1284|Rubrivivax
ASV1122|Rubrivivax
ASV1314|Actinoplanes
ASV681|Actinoplanes
ASV1472|Enterobacteriaceae
ASV1049|Streptomycetaceae
Phylum
Gammaproteobacteria
Betaproteobacteria
Actinobacteria
Alphaproteobacteria
Firmicutes
Deltaproteobacteria
Bacteroidetes
Chloroflexi
Gemmatimonadetes
Others
DCB
Depleted
Enriched
NS
(i)
SY41−Root endophytic bacteria: Inoculation vs Control
−log10(padj)
MetaCyc Pathways
ectoine biosynthesis
GDP−D−glycero−α−D−manno−heptose biosynthesis
superpathway of glycerol degradation to 1,3−propanediol
glycogen degradation II (eukaryotic)
nitrate reduction VI (assimilatory)
pyrimidine deoxyribonucleotides de novo biosynthesis III
acetyl−CoA fermentation to butanoate II
photorespiration
4−coumarate degradation (anaerobic)
DCB
Depleted
Enriched
NS

Figure S11 The variations of root endophytic bacterial communities and functions of *F. graminearum*-tolerant SHW HG116 and its susceptible parents LDN and SY41 following PH-1 infection.

(a, d and g) LDA effect size taxonomic cladogram comparing the root endophytic bacterial microbiota between control and inoculated samples of HG116 (a), LDN (d) and SY41(g), respectively. Significantly discriminant bacterial taxon nodes were colored and branch areas were shaded according to the highest-ranked variety for that taxon. If the taxon was not significantly differentially represented between sample groups, the corresponding node was colored yellow. (b, e and h) Manhattan plots showing the variations of Core ASVs in root endophytic bacterial microbiota of HG116 (b), LDN (e) and SY41(h) following *F. graminearum* infection. Core ASVs that were significantly enriched were depicted as vertical triangles, that were significantly depleted were depicted as inverted triangles, otherwise depicted as circles. The red and blue dashed line corresponded to the FDR-corrected pvalue of 0.01 and 0.05, respectively. The color of each point represented the phylum-level taxonomic affiliation of the Core ASVs, and the size corresponded to the baseMean of the Core ASVs. (c, f and i) Manhattan plots showing the shifts of MetaCyc pathways in root endophytic bacterial microbiota of HG116 (c), LDN (f) and SY41(i) following *F. graminearum* infection. Predicted bacterial functions using PICRUSt2 that were significantly enhanced were depicted as burgundy vertical triangles, that were significantly weakened were depicted as steel blue inverted triangles, otherwise depicted as grey circles. The red and blue dashed line corresponded to the FDR-corrected pvalue of 0.01 and 0.05, respectively. Each point represented each MetaCyc pathway, and the size of each point corresponded to the baseMean of functional abundance of MetaCyc pathway.

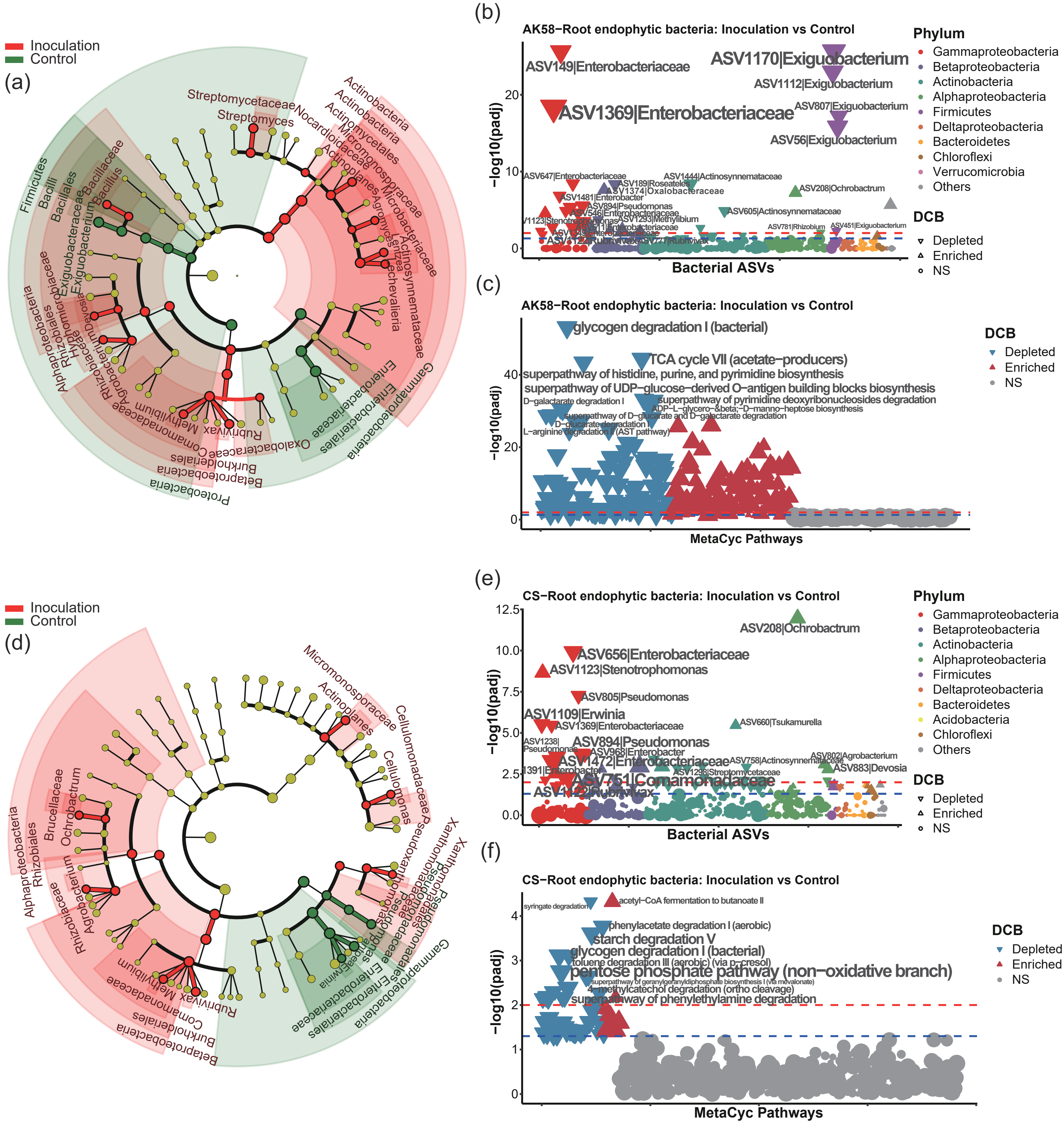

Figure S12 The variations of root endophytic bacterial communities and functions of *F. graminearum*-resistant allohexaploid AK58, and susceptible variety CS following PH-1 infection.

(a and d) LDA effect size taxonomic cladogram comparing the root endophytic bacterial microbiota between control and inoculated samples of AK58 (a) and CS (d), respectively. Significantly discriminant bacterial taxon nodes were colored and branch areas were shaded according to the highest-ranked variety for that taxon. If the taxon was not significantly differentially represented between sample groups, the corresponding node was colored yellow. (b and e) Manhattan plots showing the variations of Core ASVs in root endophytic bacterial microbiota of AK58 (b) and CS (e) following *F. graminearum* infection. Core ASVs that were significantly enriched were depicted as vertical triangles, that were significantly depleted were depicted as inverted triangles, otherwise depicted as circles. The red and blue dashed line corresponded to the FDR-corrected pvalue of 0.01 and 0.05, respectively. The color of each point represented the phylum-level taxonomic affiliation of the Core ASVs, and the size corresponded to the baseMean of the Core ASVs. (c and f) Manhattan plots showing the shifts of MetaCyc pathways in root endophytic bacterial microbiota of AK58 (c) and CS (f) following *F. graminearum* infection. Predicted bacterial functions using PICRUSt2 that were significantly enhanced were depicted as burgundy vertical triangles, that were significantly weakened were depicted as steelblue inverted triangles, otherwise depicted as grey circles. The red and blue dashed line corresponded to the FDR-corrected pvalue of 0.01 and 0.05, respectively. Each point represented each MetaCyc pathway, and the size of each point corresponded to the baseMean of functional abundance of MetaCyc pathway.

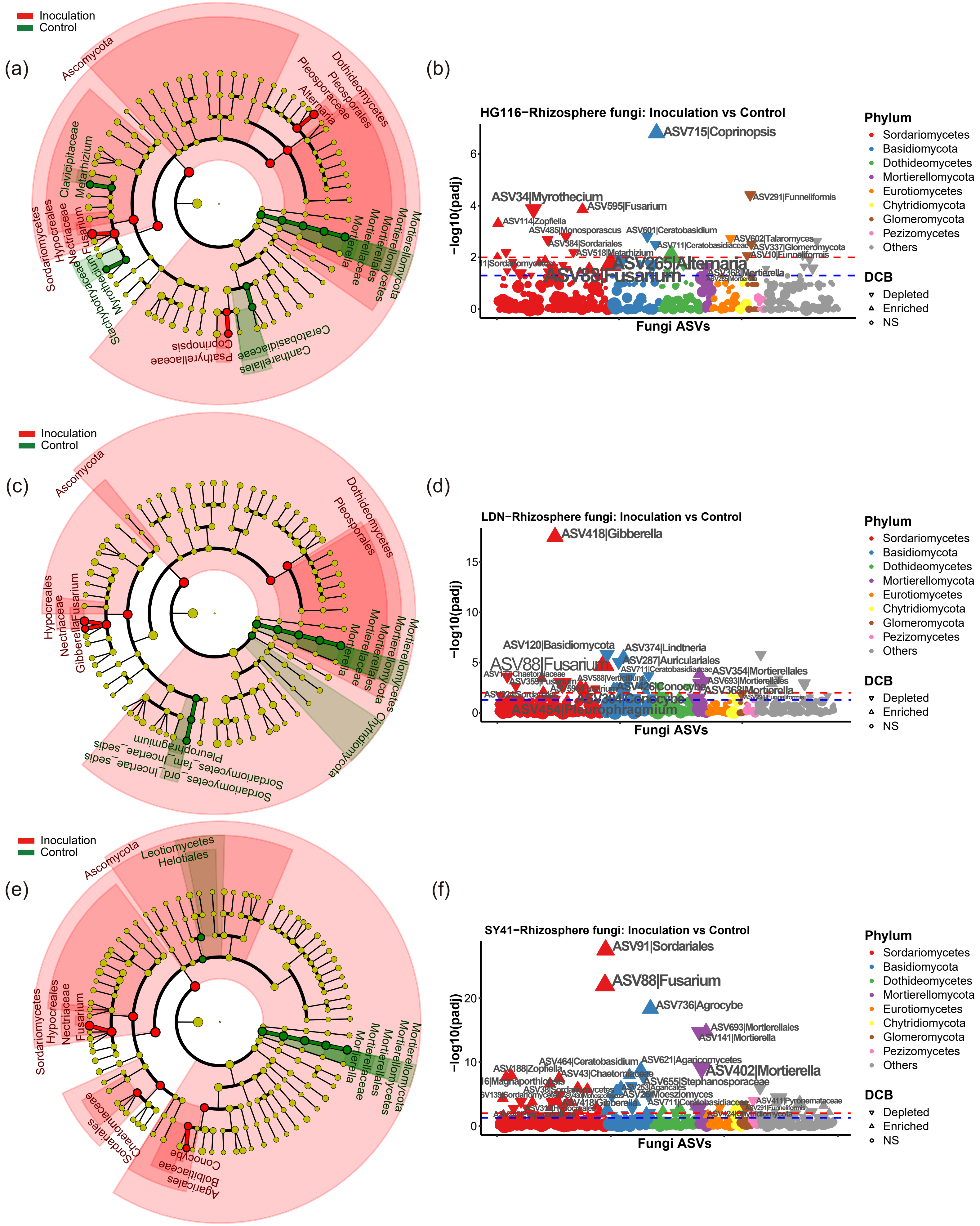

Figure S13 The variations of rhizosphere fungal communities of *F. graminearum*-tolerant SHW HG116 and its susceptible parents LDN and SY41 following PH-1 infection.
(a, c and e) LDA effect size taxonomic cladogram comparing the rhizosphere fungal microbiota between control and inoculated samples of HG116 (a), LDN (c) and SY41(e), respectively. Significantly discriminant bacterial taxon nodes were colored and branch areas were shaded according to the highest-ranked variety for that taxon. If the taxon was not significantly differentially represented between sample groups, the corresponding node was colored yellow. (b, d and f) Manhattan plots showing the variations of Core ASVs in rhizosphere fungal microbiota of HG116 (b), LDN (d) and SY41(f) following *F. graminearum* infection.Core ASVs that were significantly enriched were depicted as vertical triangles, that were significantly depleted were depicted as inverted triangles, otherwise depicted as circles. The red and blue dashed line corresponded to the FDR-corrected pvalue of 0.01 and 0.05, respectively. The color of each point represented the phylum-level taxonomic affiliation of the Core ASVs, and the size corresponded to the baseMean of the Core ASVs.

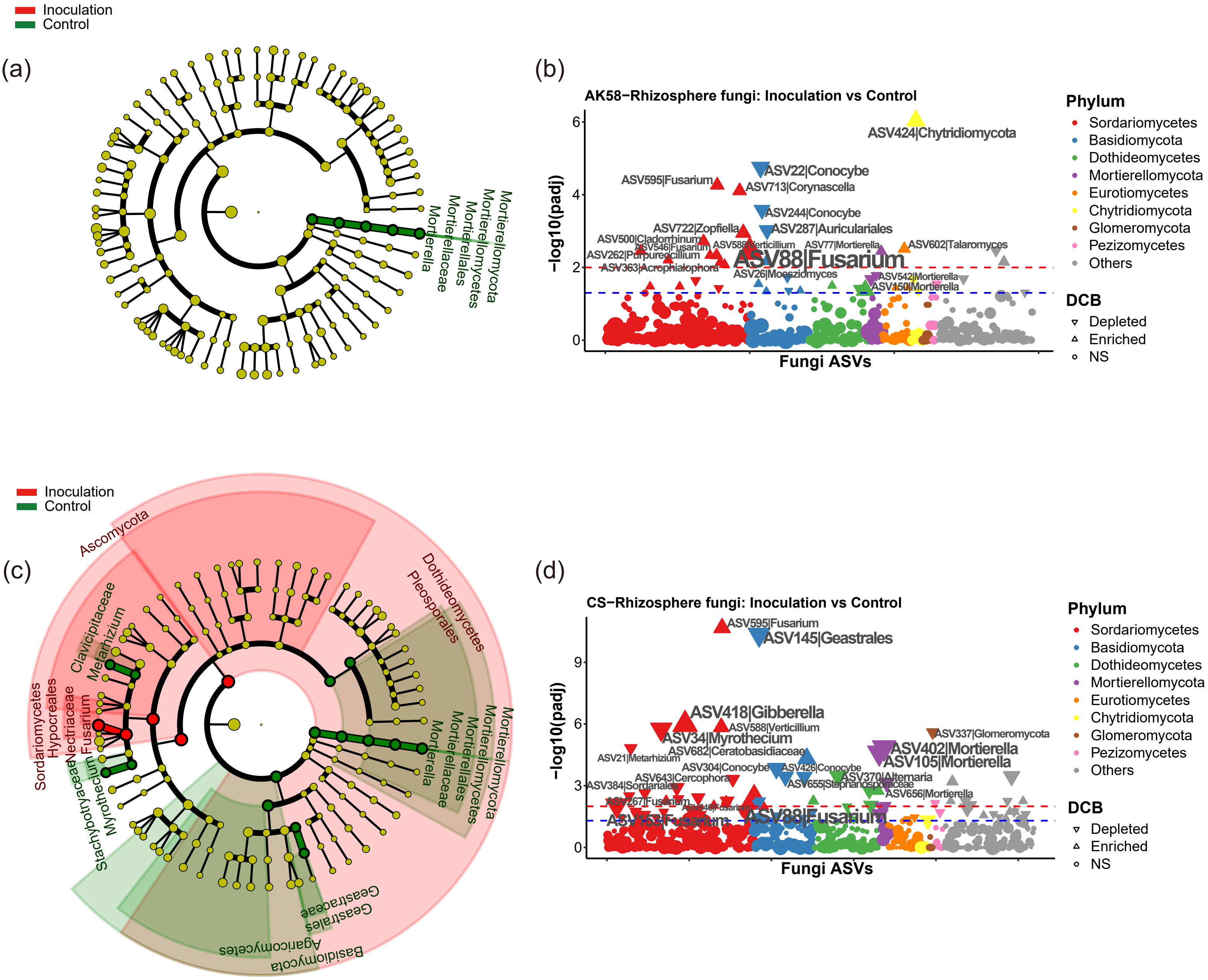

Figure S14 The variations of rhizosphere fungal communities of *F. graminearum*-resistant allohexaploid AK58 and susceptible variety CS following PH-1 infection. (a and c) LDA effect size taxonomic cladogram comparing the rhizosphere fungal microbiota between control and inoculated samples of AK58 (a) and CS (c), respectively. Significantly discriminant bacterial taxon nodes were colored and branch areas were shaded according to the highest-ranked variety for that taxon. If the taxon was not significantly differentially represented between sample groups, the corresponding node was colored yellow. (b and d) Manhattan plots showing the variations of Core ASVs in rhizosphere fungal microbiota of AK58 (b) and CS (d) following *F. graminearum* infection. Core ASVs that were significantly enriched were depicted as vertical triangles, that were significantly depleted were depicted as inverted triangles, otherwise depicted as circles. The red and blue dashed line corresponded to the FDR-corrected pvalue of 0.01 and 0.05, respectively. The color of each point represented the phylum-level taxonomic affiliation of the Core ASVs, and the size corresponded to the baseMean of the Core ASVs.

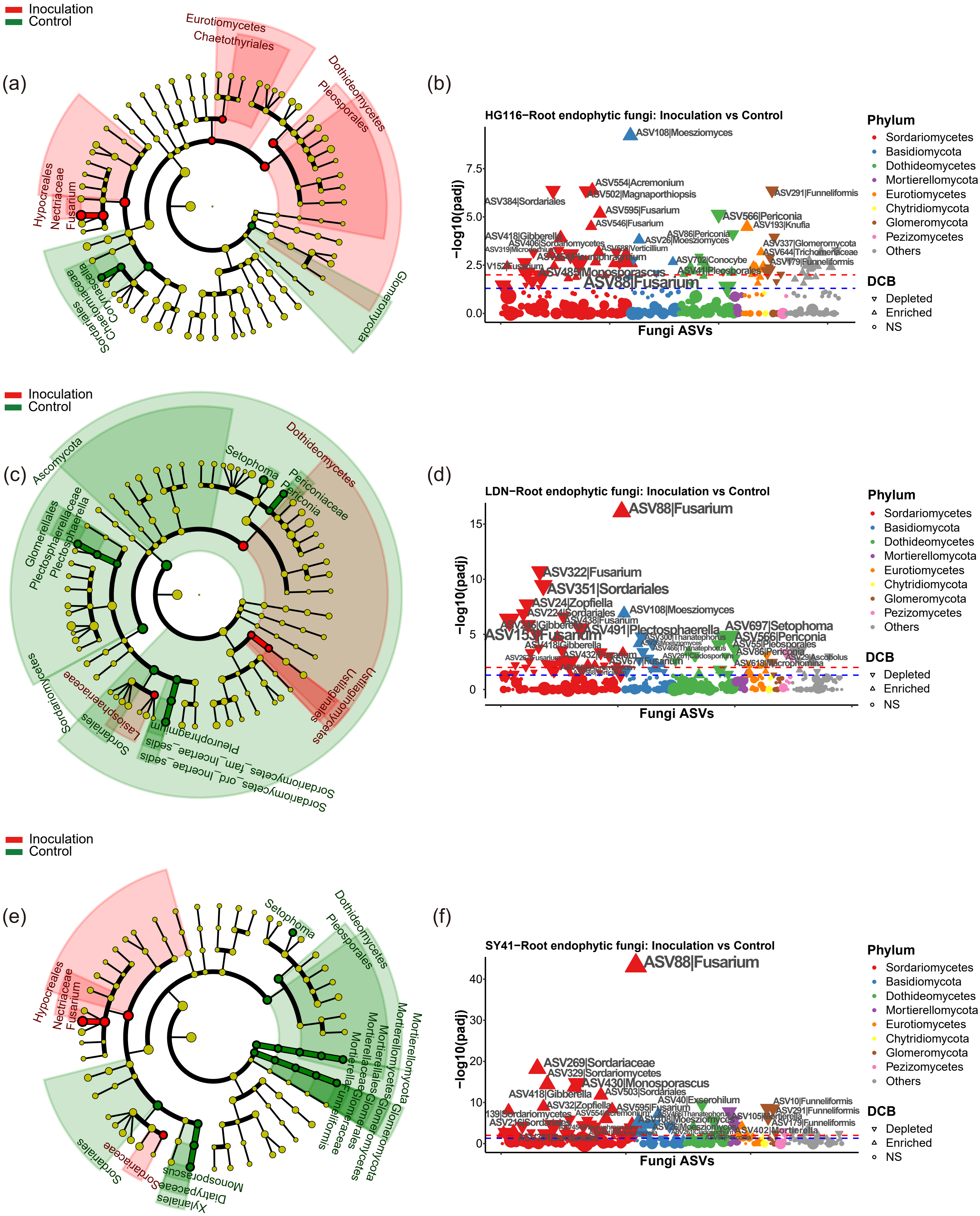

Figure S15 The variations of root endophytic fungal communities of *F. graminearum*-tolerant SHW HG116 and its susceptible parents LDN and SY41 following PH-1 infection.(a, c and e) LDA effect size taxonomic cladogram comparing the root endophytic fungal microbiota between control and inoculated samples of HG116 (a), LDN (c) and SY41(e), respectively. Significantly discriminant bacterial taxon nodes were colored and branch areas were shaded according to the highest-ranked variety for that taxon. If the taxon was not significantly differentially represented between sample groups, the corresponding node was colored yellow. (b, d and f) Manhattan plots showing the variations of Core ASVs in root endophytic fungal microbiota of HG116 (b), LDN (d) and SY41(f) following *F. graminearum* infection. Core ASVs that were significantly enriched were depicted as vertical triangles, that were significantly depleted were depicted as inverted triangles, otherwise depicted as circles. The red and blue dashed line corresponded to the FDR-corrected pvalue of 0.01 and 0.05, respectively. The color of each point represented the phylum-level taxonomic affiliation of the Core ASVs, and the size corresponded to the baseMean of the Core ASVs.

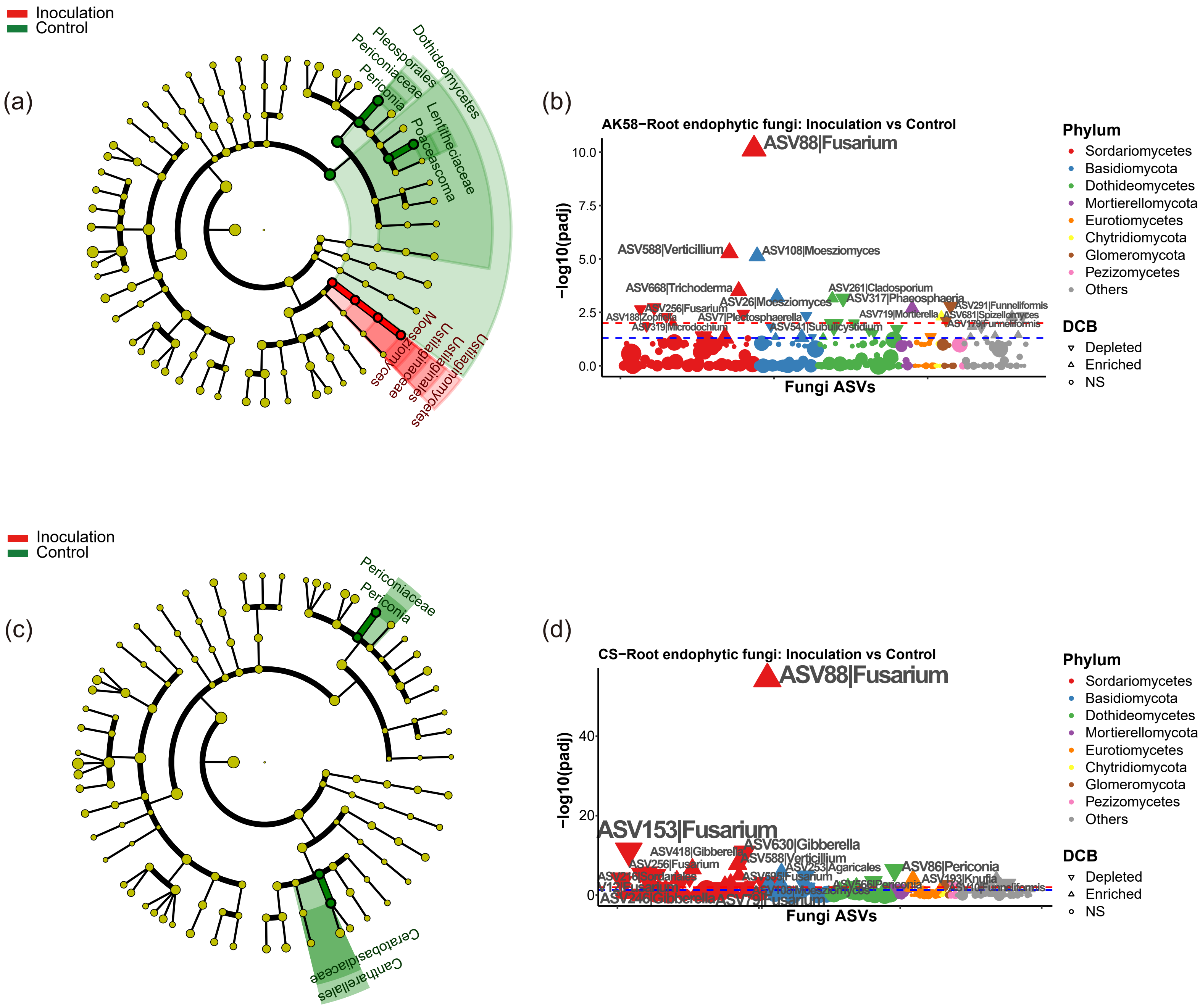

Figure S16 The variations of root endophytic fungal communities of *F. graminearum*-resistant allohexaploid AK58 and susceptible variety CS following PH-1 infection.

(a and c) LDA effect size taxonomic cladogram comparing the root endophytic fungal microbiota between control and inoculated samples of AK58 (a) and CS (c), respectively. Significantly discriminant bacterial taxon nodes were colored and branch areas were shaded according to the highest-ranked variety for that taxon. If the taxon was not significantly differentially represented between sample groups, the corresponding node was colored yellow. (b and d) Manhattan plots showing the variations of Core ASVs in root endophytic fungal microbiota of AK58 (b) and CS (d) following *F. graminearum* infection. Core ASVs that were significantly enriched were depicted as vertical triangles, that were significantly depleted were depicted as inverted triangles, otherwise depicted as circles. The red and blue dashed line corresponded to the FDR-corrected pvalue of 0.01 and 0.05, respectively. The color of each point represented the phylum-level taxonomic affiliation of the Core ASVs, and the size corresponded to the baseMean of the Core ASVs.

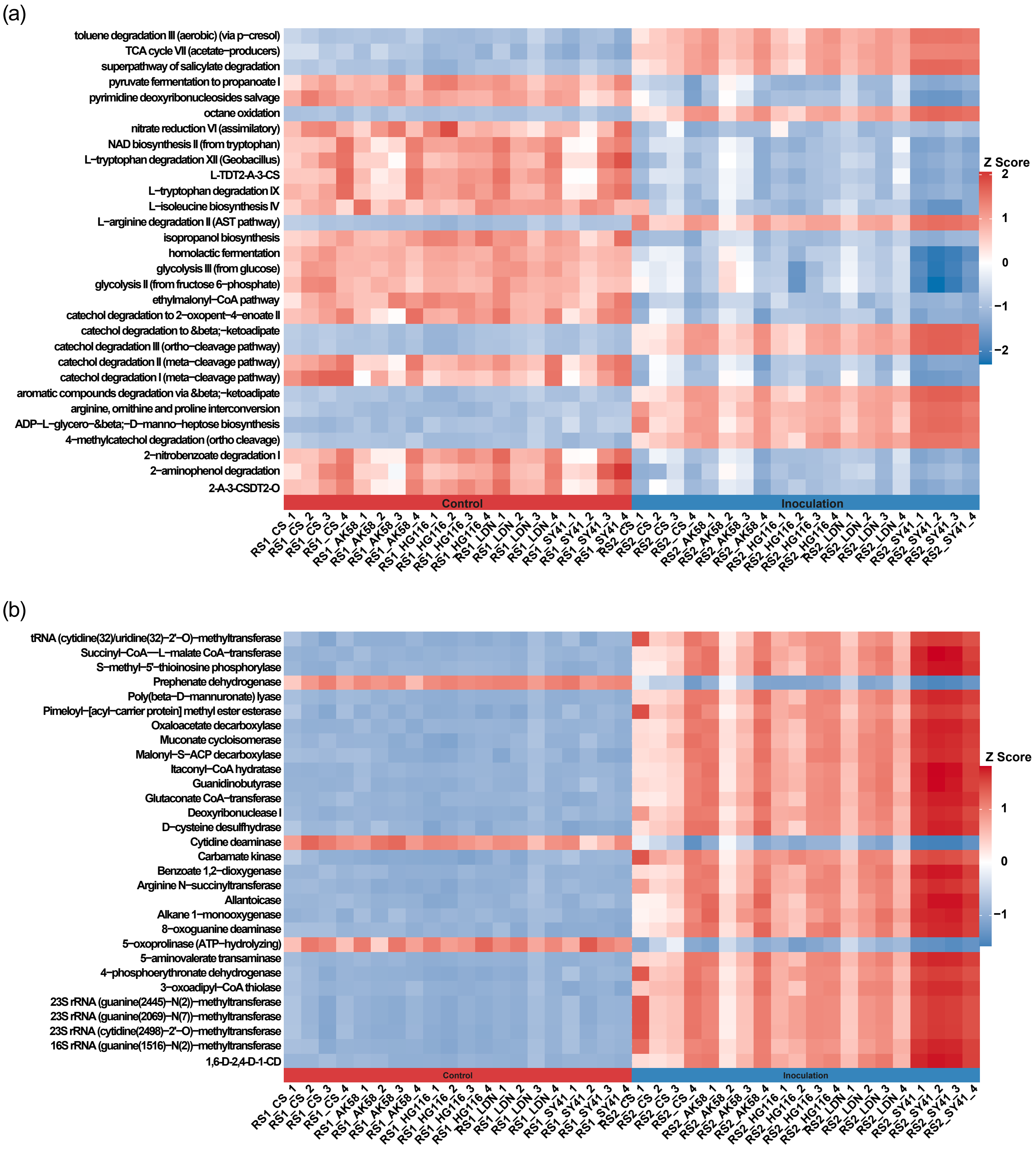

Figure S17 Heatmap of functional profiles with significant differences predicted by PICRUSt2 between wheat varieties with different disease resistances and their respective controls.

(a-b) Heatmap of the top 30 MetaCyc pathways of BH-adjusted p values (BH-adjusted pvalue < 0.05) between control and inoculated groups in rhizosphere (a) and root endophytic (b) bacterial microbiota of wheat varieties with different disease-resistances. The color indicated high or low functional abundances of MetaCyc pathways. The functional abundances of MetaCyc pathways had been standardized using Z-score. L-TDT2-A-3-CS: L-tryptophan degradation to 2-amino-3-carboxymuconate semialdehyde; 2-A-3-CSDT2-O: 2-amino-3-carboxymuconate semialdehyde degradation to 2-oxopentenoate; 1,6-D-2,4-D-1-CD: 1,6-dihydroxycyclohexa-2,4-diene-1-carboxylate dehydrogenase.

(a)

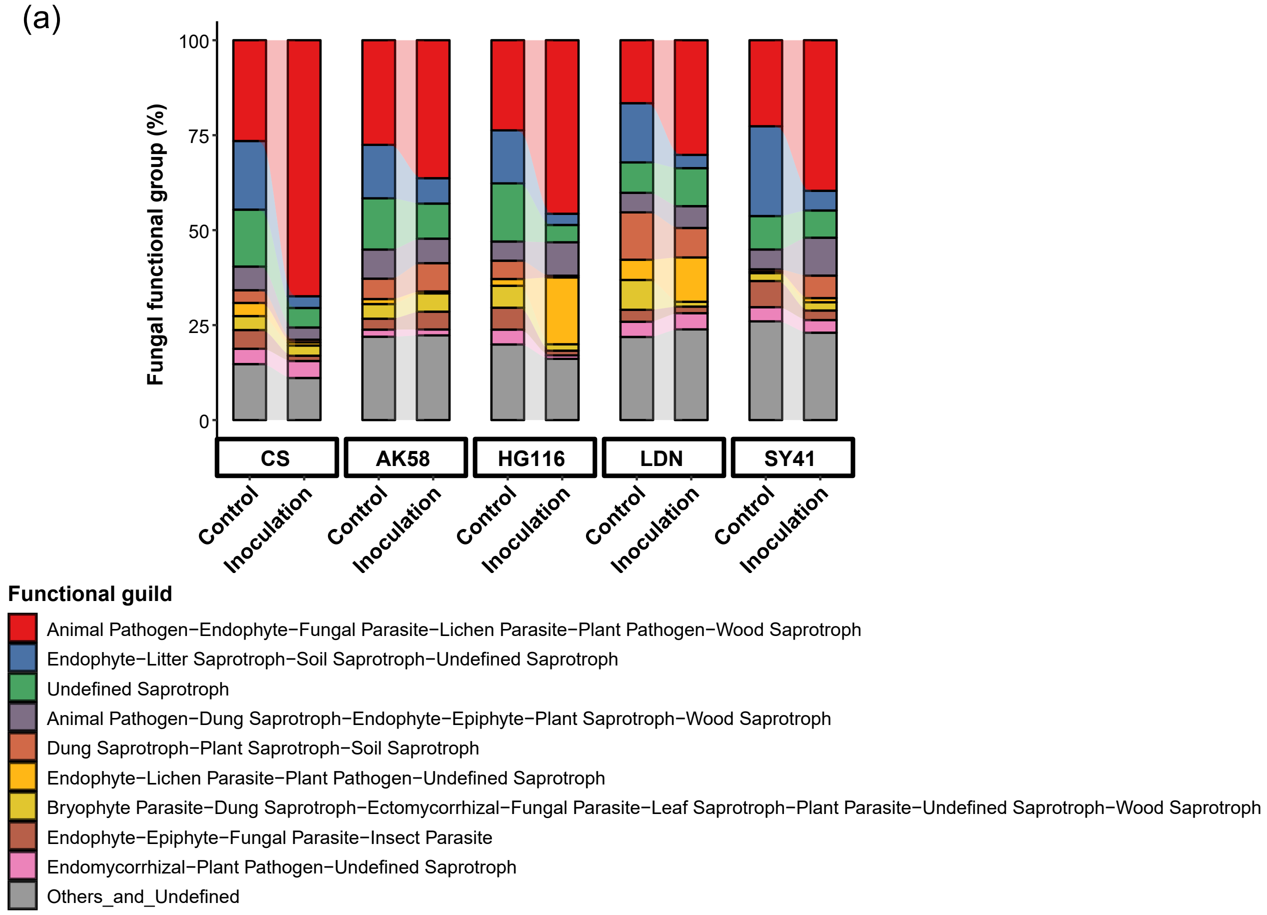

(b)

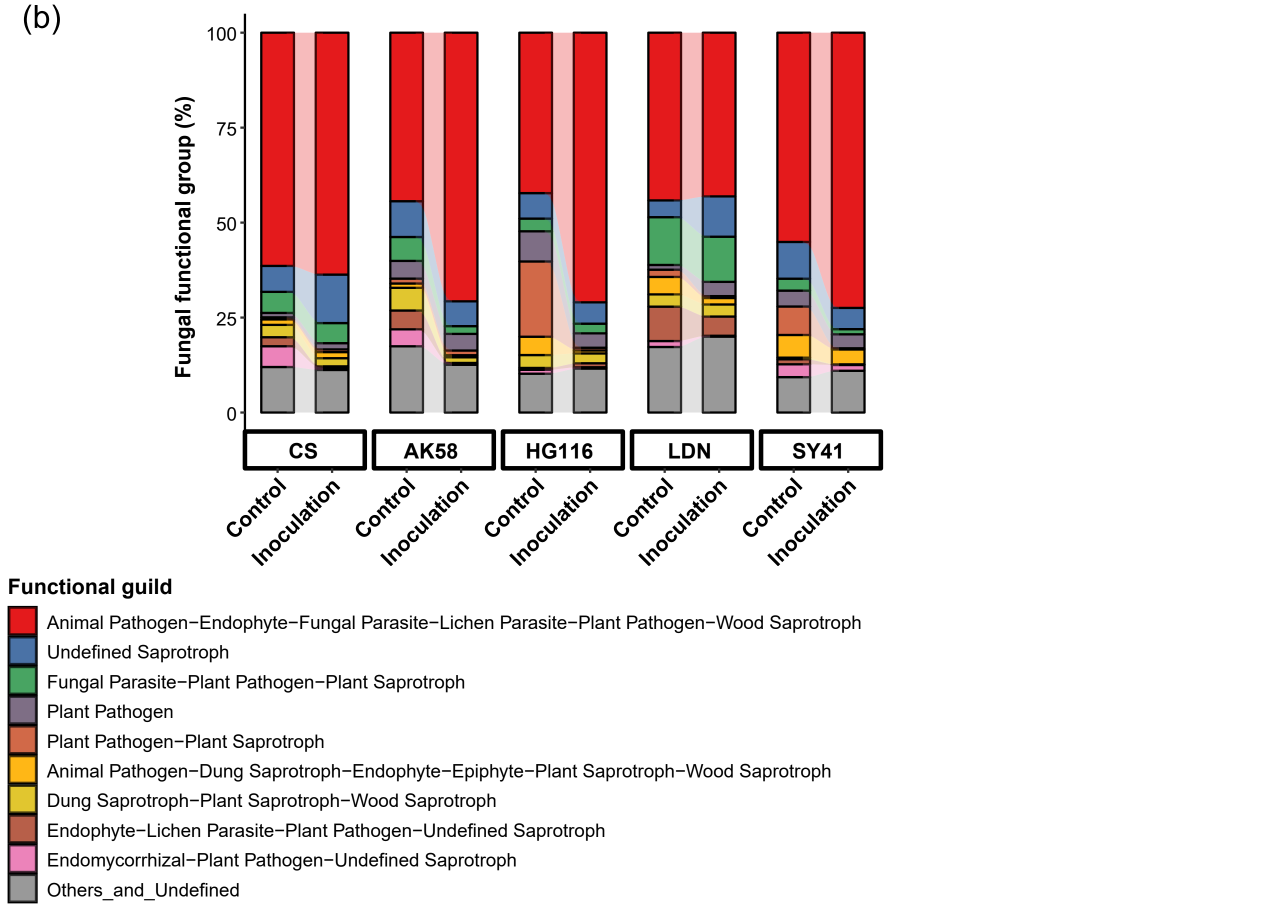

Figure S18 Functional shifts in rhizosphere and root endophytic fungal microbiota across wheat varieties following PH-1 infection.
(a-b) Comparison of the functional guild distribution of the rhizosphere (a) and root endophytic (b) fungal microbiota between wheat varieties with different disease-resistances and their respective controls. Average functional guild abundances of 4 biological replicates were displayed in separate stacked bars.

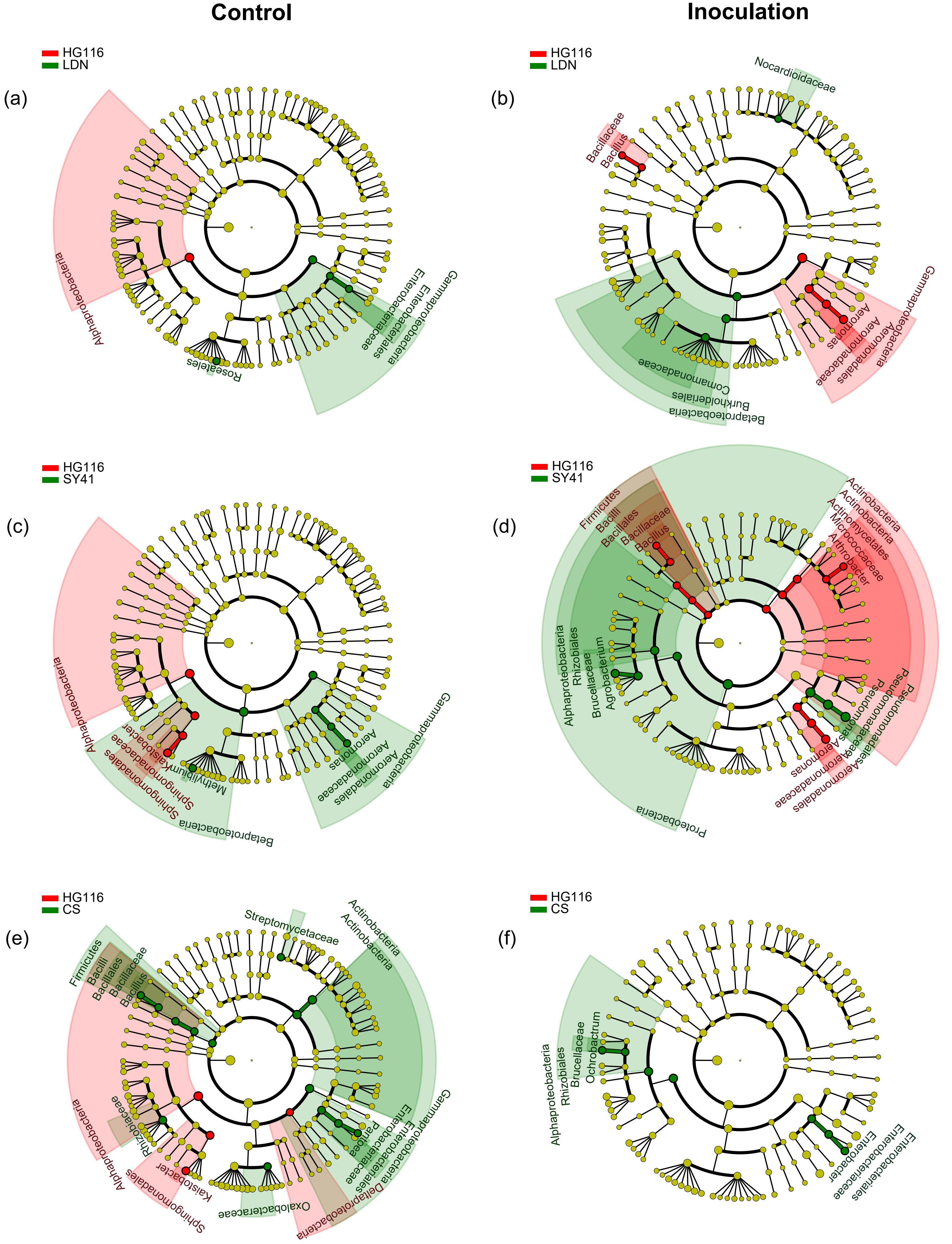

Figure S19 Comparison of the differential rhizosphere bacterial biomarkers between *F. graminearum*-tolerant SHW HG116 and other susceptible wheat varieties by LEfSe analysis.

(a-f). LDA effect size taxonomic cladogram comparing rhizosphere bacterial microbiota between HG116 and LDN control samples (a), between HG116 and LDN inoculated samples (b), between HG116 and SY41 control samples (c), between HG116 and SY41 inoculated samples (d), between HG116 and CS control samples (e), and between HG116 and CS inoculated samples (f). Significantly discriminant bacterial taxon nodes were colored and branch areas were shaded according to the highest-ranked variety for that taxon. If the taxon was not significantly differentially represented between sample groups, the corresponding node was colored yellow.

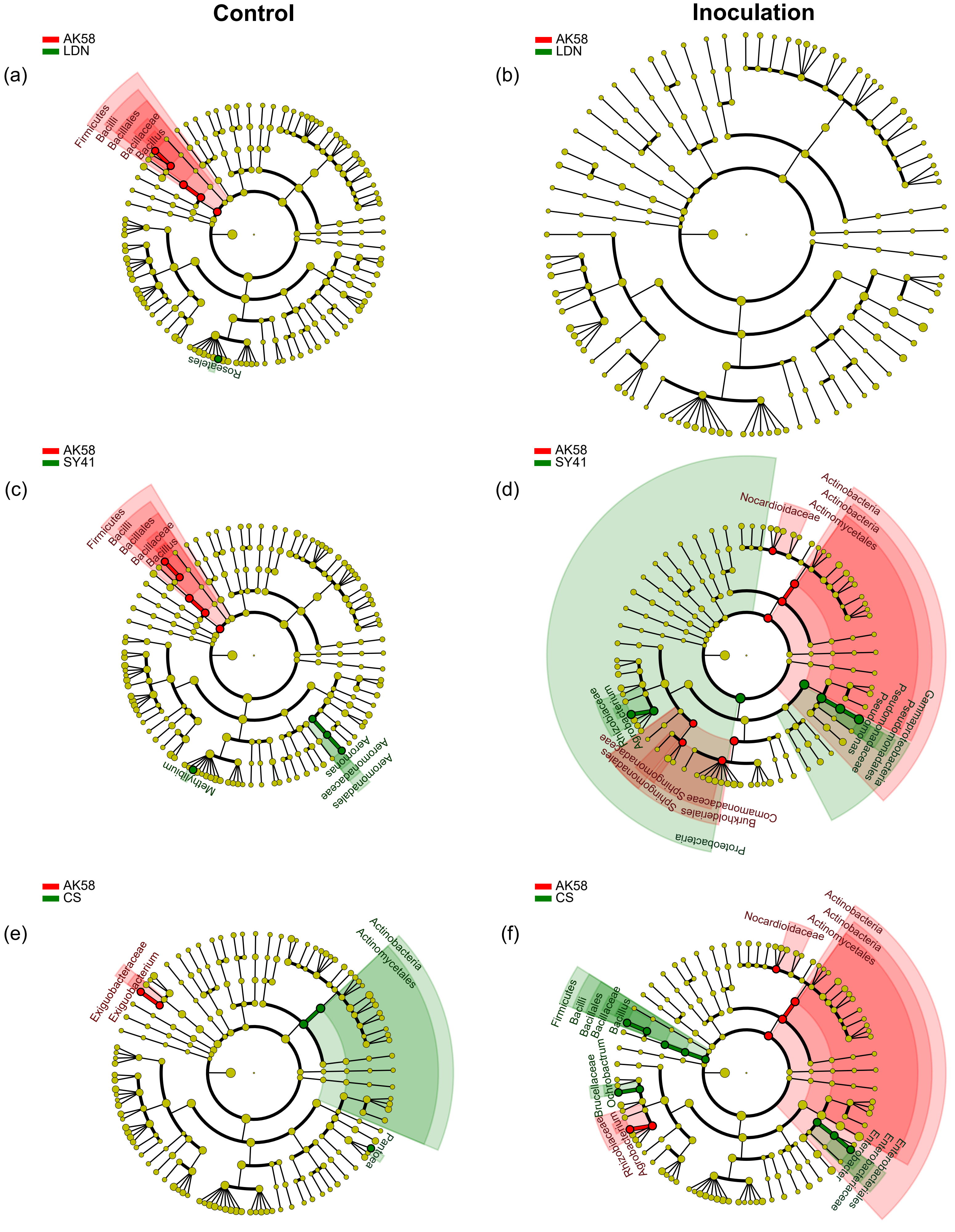

Figure S20 Comparison of the differential rhizosphere bacterial biomarkers between *F. graminearum*-resistant allohexaploid AK58 and other susceptible wheat varieties by LEfSe analysis.
(a-f). LDA effect size taxonomic cladogram comparing rhizosphere bacterial microbiota between AK58 and LDN control samples (a), between AK58 and LDN inoculated samples (b), AK58 and SY41 control samples (c), between AK58 and SY41 inoculated samples (d), between AK58 and CS control samples (e), and between AK58 and CS inoculated samples (f). Significantly discriminant bacterial taxon nodes were colored and branch areas were shaded according to the highest-ranked variety for that taxon. If the taxon was not significantly differentially represented between sample groups, the corresponding node was colored yellow.

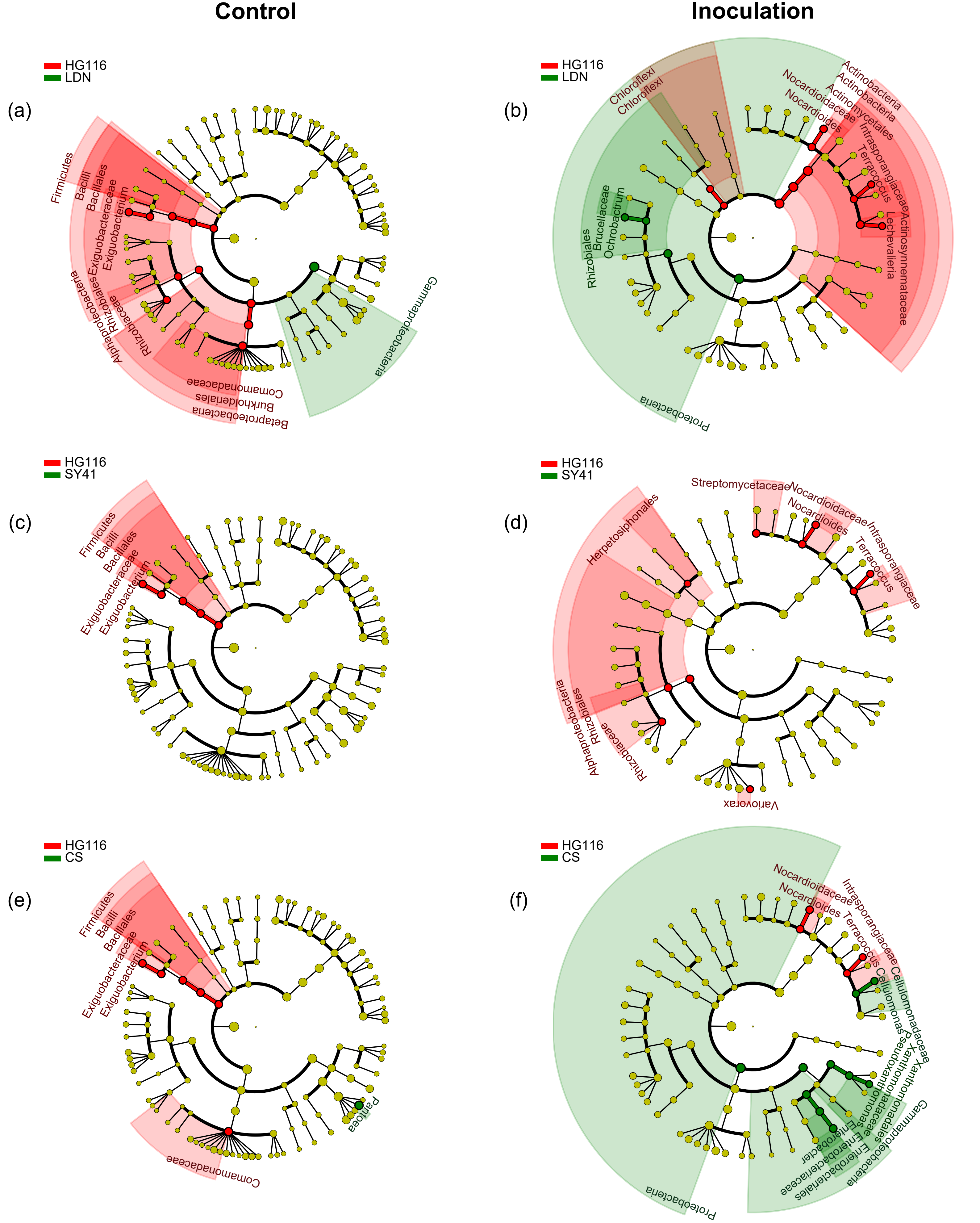

Figure S21 Comparison of the differential root endophytic bacterial biomarkers between *F. graminearum*-tolerant SHW HG116 and other susceptible wheat varieties by LEfSe analysis.

(a-f). LDA effect size taxonomic cladogram comparing root endophytic bacterial microbiota between HG116 and LDN control samples (a), between HG116 and LDN inoculated samples (b), between HG116 and SY41 control samples (c), between HG116 and SY41 inoculated samples (d), between HG116 and CS control samples (e), and between HG116 and CS inoculated samples (f). Significantly discriminant bacterial taxon nodes were colored and branch areas were shaded according to the highest-ranked variety for that taxon. If the taxon was not significantly differentially represented between sample groups, the corresponding node was colored yellow.

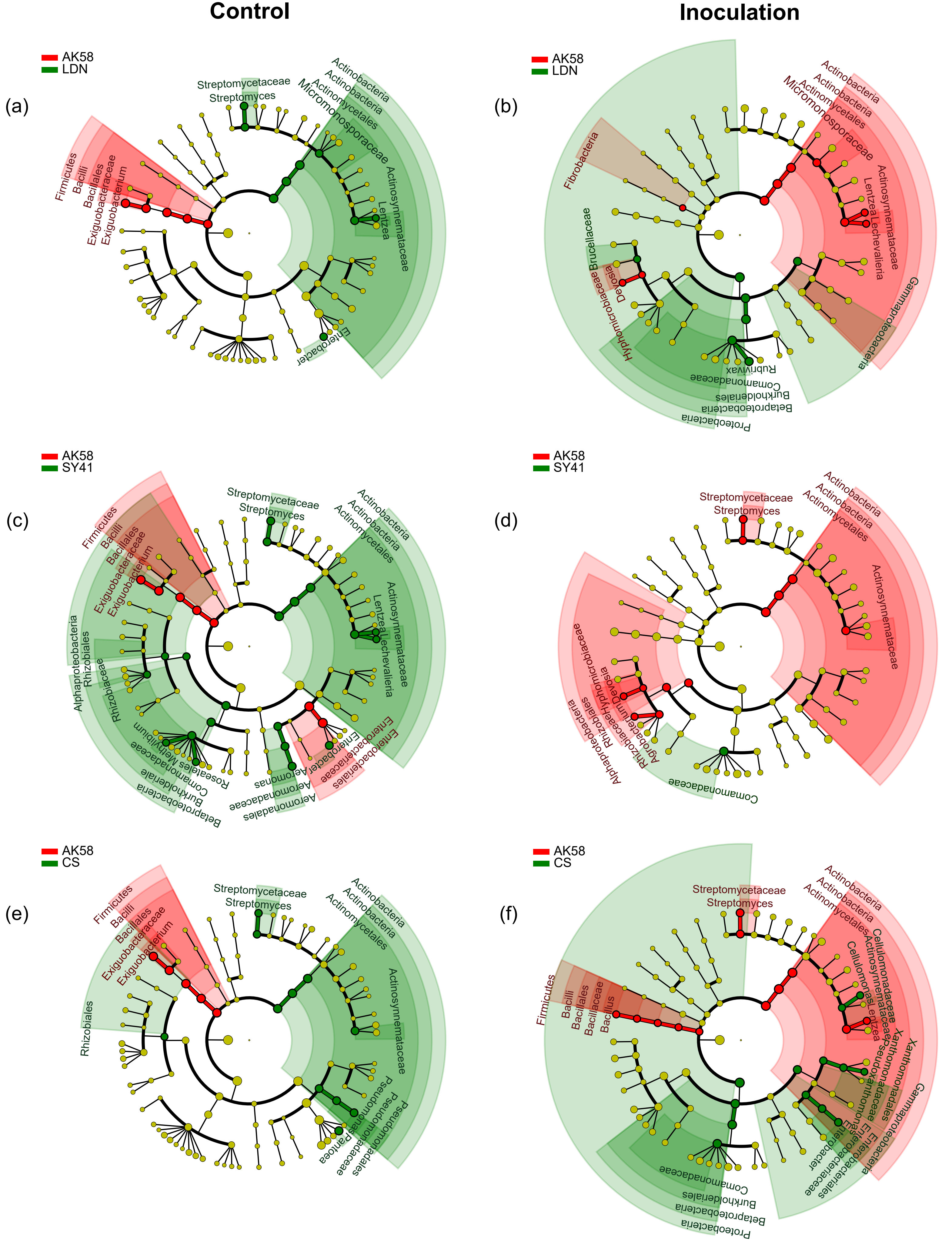

Figure S22 Comparison of the differential root endophytic bacterial biomarkers between *F. graminearum*-resistant allohexaploid AK58 and other susceptible wheat varieties by LEfSe analysis.

(a-f). LDA effect size taxonomic cladogram comparing root endophytic bacterial microbiota between AK58 and LDN control samples (a), between AK58 and LDN inoculated samples (b), AK58 and SY41 control samples (c), between AK58 and SY41 inoculated samples (d), between AK58 and CS control samples (e), and between AK58 and CS inoculated samples (f). Significantly discriminant bacterial taxon nodes were colored and branch areas were shaded according to the highest-ranked variety for that taxon. If the taxon was not significantly differentially represented between sample groups, the corresponding node was colored yellow.

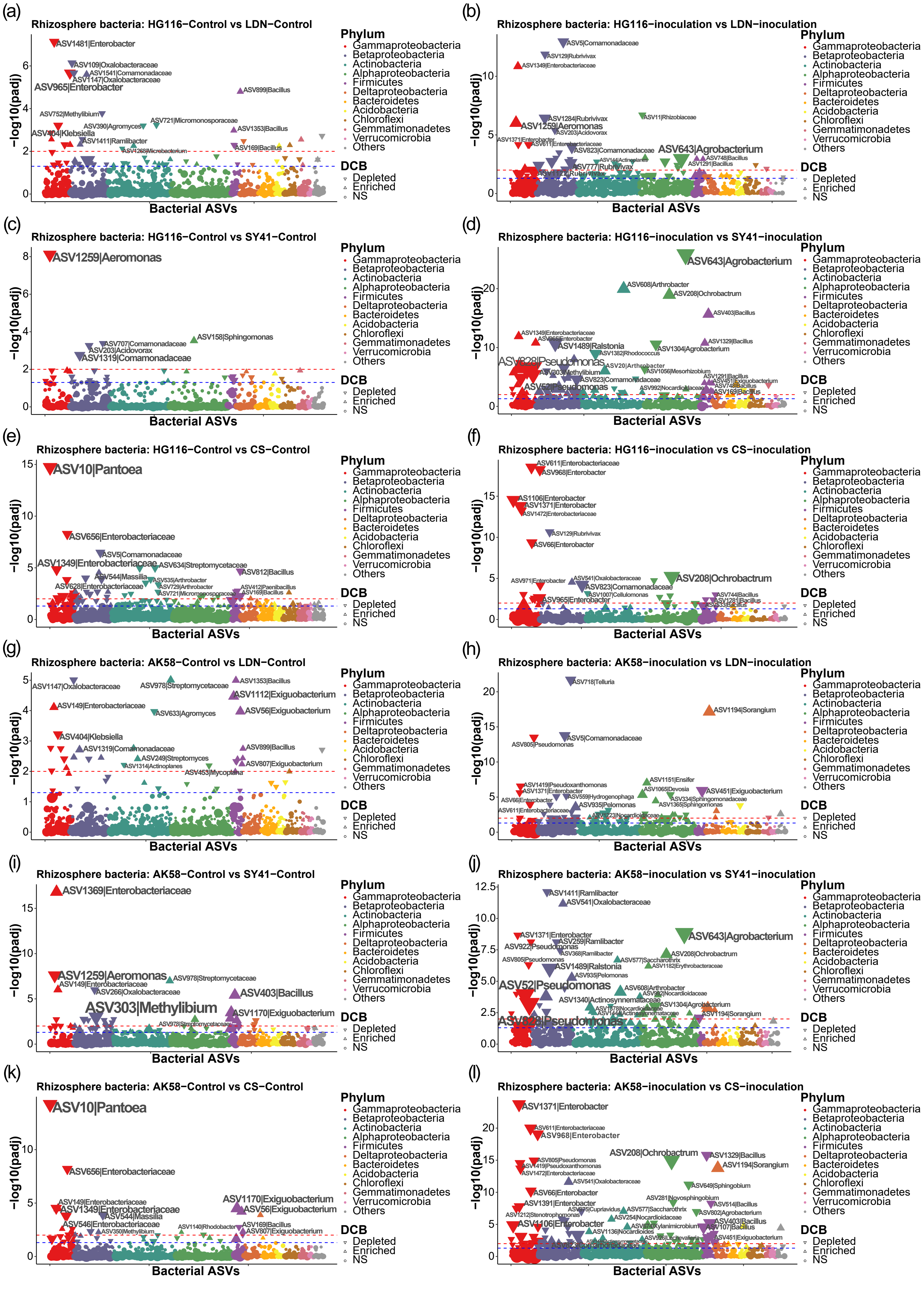

(a)
Rhizosphere bacteria: HG116−Control vs LDN−Control
ASV1481|Enterobacter
ASV109|Oxalobacteraceae
ASV1541|Comamonadaceae
ASV1147|Oxalobacteraceae
ASV965|Enterobacter
ASV899|Bacillus
ASV752|Methylibium
ASV721|Micromonosporaceae
ASV390|Agromyces
ASV404|Klebsiella
ASV1353|Bacillus
ASV1411|Ramlibacter
ASV4268|Microbacterium
ASV169|Bacillus
−log10(padj)
Bacterial ASVs
(b)
Rhizosphere bacteria: HG116−inoculation vs LDN−inoculation
ASV5|Comamonadaceae
ASV129|Rubrivivax
ASV1349|Enterobacteriaceae
ASV1284|Rubrivivax
ASV11|Rhizobiaceae
ASV1259|Aeromonas
ASV203|Acidovorax
ASV1371|Enterobacter
ASV611|Enterobacteriaceae
ASV823|Comamonadaceae
ASV643|Agrobacterium
ASV144|Actinoplanes
ASV748|Bacillus
ASV1291|Bacillus
ASV777|Rubrivivax
ASV1122|Rubrivivax
−log10(padj)
Bacterial ASVs
(c)
Rhizosphere bacteria: HG116−Control vs SY41−Control
ASV1259|Aeromonas
ASV158|Sphingomonas
ASV707|Comamonadaceae
ASV203|Acidovorax
ASV1319|Comamonadaceae
−log10(padj)
Bacterial ASVs
(d)
Rhizosphere bacteria: HG116−inoculation vs SY41−inoculation
ASV643|Agrobacterium
ASV608|Arthrobacter
ASV208|Ochrobactrum
ASV403|Bacillus
ASV1349|Enterobacteriaceae
ASV965|Enterobacter
ASV1489|Ralstonia
ASV1329|Bacillus
ASV1304|Agrobacterium
ASV1382|Rhodococcus
ASV828|Pseudomonas
ASV20|Arthrobacter
ASV303|Methylibium
ASV1056|Mesorhizobium
ASV823|Comamonadaceae
ASV1291|Bacillus
ASV451|Exiguobacterium
ASV52|Pseudomonas
ASV992|Nocardioidaceae
ASV748|Bacillus
ASV169|Bacillus
−log10(padj)
Bacterial ASVs
(e)
Rhizosphere bacteria: HG116−Control vs CS−Control
ASV10|Pantoea
ASV656|Enterobacteriaceae
ASV5|Comamonadaceae
ASV1349|Enterobacteriaceae
ASV634|Streptomycetaceae
ASV812|Bacillus
ASV544|Massilia
ASV535|Arthrobacter
ASV628|Enterobacteriaceae
ASV729|Arthrobacter
ASV412|Paenibacillus
ASV721|Micromonosporaceae
ASV169|Bacillus
−log10(padj)
Bacterial ASVs
(f)
Rhizosphere bacteria: HG116−inoculation vs CS−inoculation
ASV611|Enterobacteriaceae
ASV968|Enterobacter
AS1106|Enterobacter
ASV1371|Enterobacter
ASV1472|Enterobacteriaceae
ASV129|Rubrivivax
ASV66|Enterobacter
ASV971|Enterobacter
ASV541|Oxalobacteraceae
ASV208|Ochrobactrum
ASV823|Comamonadaceae
ASV1007|Cellulomonas
ASV965|Enterobacter
ASV744|Bacillus
ASV1281|Bacillus
ASV533|Bacillus
−log10(padj)
Bacterial ASVs
(g)
Rhizosphere bacteria: AK58−Control vs LDN−Control
ASV1147|Oxalobacteraceae
ASV978|Streptomycetaceae
ASV1353|Bacillus
ASV1112|Exiguobacterium
ASV149|Enterobacteriaceae
ASV633|Agromyces
ASV56|Exiguobacterium
ASV404|Klebsiella
ASV1319|Comamonadaceae
ASV899|Bacillus
ASV249|Streptomyces
ASV1314|Actinoplanes
ASV807|Exiguobacterium
ASV453|Mycoplana
−log10(padj)
Bacterial ASVs
(h)
Rhizosphere bacteria: AK58−inoculation vs LDN−inoculation
ASV718|Telluria
ASV1194|Sorangium
ASV5|Comamonadaceae
ASV805|Pseudomonas
ASV1151|Ensifer
ASV1419|Pseudoxanthomonas
ASV1065|Devosia
ASV1371|Enterobacter
ASV451|Exiguobacterium
ASV559|Hydrogenophaga
ASV334|Sphingomonadaceae
ASV66|Enterobacter
ASV935|Pelomonas
ASV1365|Sphingomonas
ASV611|Enterobacteriaceae
ASV1223|Nocardioidaceae
−log10(padj)
Bacterial ASVs
(i)
Rhizosphere bacteria: AK58−Control vs SY41−Control
ASV1369|Enterobacteriaceae
ASV1259|Aeromonas
ASV978|Streptomycetaceae
ASV149|Enterobacteriaceae
ASV266|Oxalobacteraceae
ASV403|Bacillus
ASV303|Methylibium
ASV1170|Exiguobacterium
ASV978|Streptomycetaceae
−log10(padj)
Bacterial ASVs
(j)
Rhizosphere bacteria: AK58−inoculation vs SY41−inoculation
ASV1411|Ramlibacter
ASV541|Oxalobacteraceae
ASV1371|Enterobacter
ASV259|Ramlibacter
ASV922|Pseudomonas
ASV643|Agrobacterium
ASV368|Ramlibacter
ASV208|Ochrobactrum
ASV805|Pseudomonas
ASV577|Saccharothrix
ASV1489|Ralstonia
ASV1182|Erythrobacteraceae
ASV935|Pelomonas
ASV52|Pseudomonas
ASV608|Arthrobacter
ASV992|Nocardioidaceae
ASV1340|Actinosynnemataceae
ASV1304|Agrobacterium
ASV1194|Sorangium
ASV828|Pseudomonas
−log10(padj)
Bacterial ASVs
(k)
Rhizosphere bacteria: AK58−Control vs CS−Control
ASV10|Pantoea
ASV656|Enterobacteriaceae
ASV149|Enterobacteriaceae
ASV1170|Exiguobacterium
ASV1349|Enterobacteriaceae
ASV56|Exiguobacterium
ASV544|Massilia
ASV546|Enterobacteriaceae
ASV1140|Rhodobacter
ASV169|Bacillus
ASV350|Methylibium
ASV807|Exiguobacterium
−log10(padj)
Bacterial ASVs
(l)
Rhizosphere bacteria: AK58−inoculation vs CS−inoculation
ASV1371|Enterobacter
ASV611|Enterobacteriaceae
ASV968|Enterobacter
ASV208|Ochrobactrum
ASV1329|Bacillus
ASV805|Pseudomonas
ASV1194|Sorangium
ASV1419|Pseudoxanthomonas
ASV1472|Enterobacteriaceae
ASV541|Oxalobacteraceae
ASV649|Sphingobium
ASV66|Enterobacter
ASV281|Novosphingobium
ASV1391|Enterobacter
ASV514|Bacillus
ASV1212|Stenotrophomonas
ASV675|Cupriavidus
ASV577|Saccharothrix
ASV802|Agrobacterium
ASV1106|Enterobacter
ASV254|Nocardioidaceae
ASV403|Bacillus
ASV1136|Nocardioides
ASV74|Xylanimicrobium
ASV107|Bacillus
ASV926|Leclercia
ASV451|Exiguobacterium
−log10(padj)
Bacterial ASVs
Phylum
Gammaproteobacteria
Betaproteobacteria
Actinobacteria
Alphaproteobacteria
Firmicutes
Deltaproteobacteria
Bacteroidetes
Acidobacteria
Chloroflexi
Gemmatimonadetes
Verrucomicrobia
Others
DCB
Depleted
Enriched
NS

Figure S23 Comparison of the differential rhizosphere bacteria Core ASVs between *F. graminearum*-tolerant and -resistant hexaploid wheats (HG116 and AK58) and other susceptible wheat varieties based on DESeq2 analysis.
(a-l) Manhattan plots showing the differences of Core ASVs in rhizosphere bacteria microbiota between HG116 and LDN control samples (a), between HG116 and LDN inoculated samples (b), between HG116 and SY41 control samples (c), between HG116 and SY41 inoculated samples (d), between HG116 and CS control samples (e), between HG116 and CS inoculated samples (f), between AK58 and LDN control samples (g), between AK58 and LDN inoculated samples (h), between AK58 and SY41 control samples (i), between AK58 and SY41 inoculated samples (j), between AK58 and CS control samples (k), and between AK58 and CS inoculated samples (l). Core ASVs that were significantly enriched were depicted as vertical triangles, that were significantly depleted were depicted as inverted triangles, otherwise depicted as circles. The red and blue dashed line corresponded to the FDR-corrected pvalue of 0.01 and 0.05, respectively. The color of each point represented the phylum-level taxonomic affiliation of the Core ASVs, and the size corresponded to the baseMean of the Core ASVs.

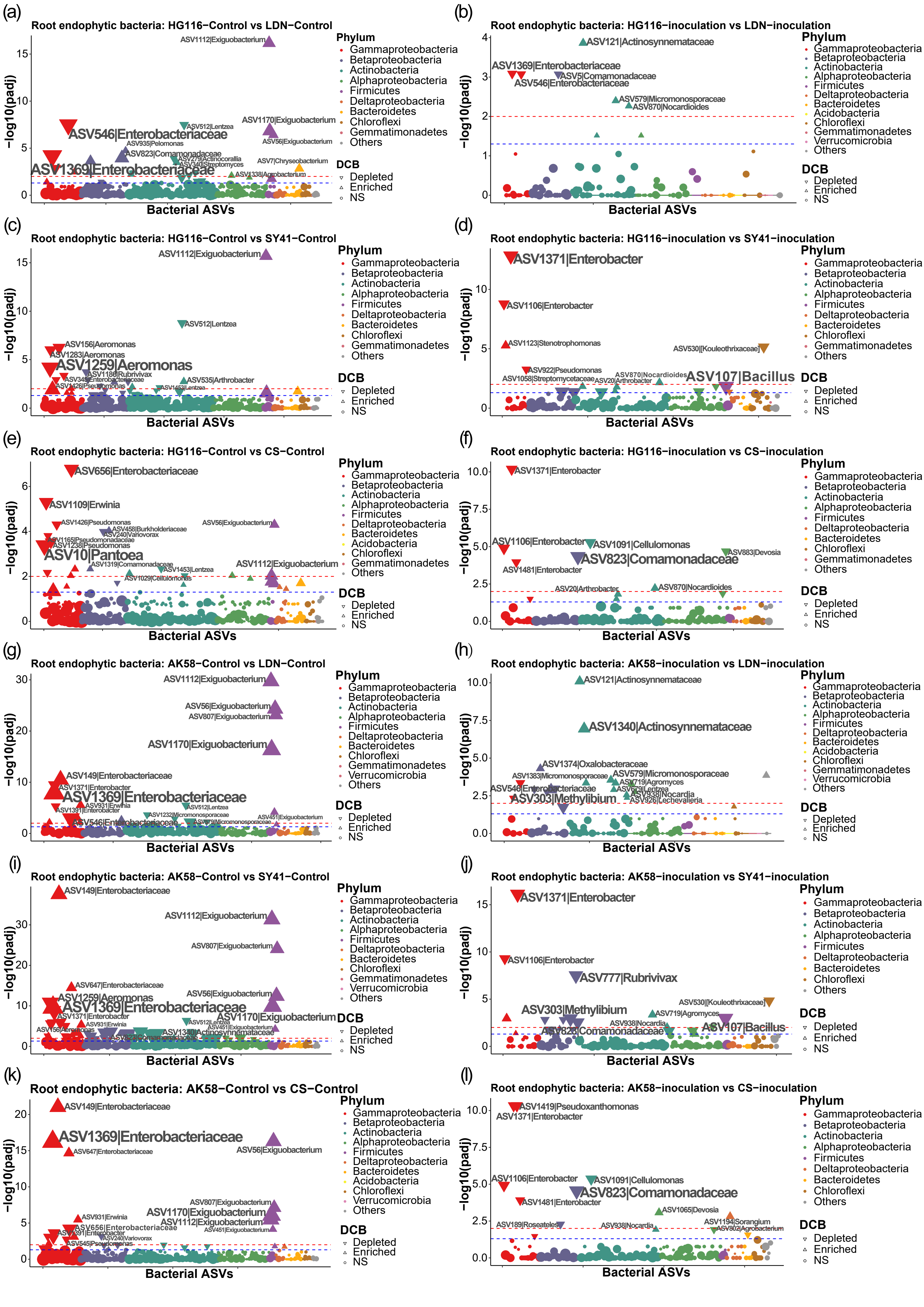

(a)
Root endophytic bacteria: HG116−Control vs LDN−Control
ASV1112|Exiguobacterium
ASV546|Enterobacteriaceae
ASV1170|Exiguobacterium
ASV512|Lentzea
ASV56|Exiguobacterium
ASV935|Pelomonas
ASV823|Comamonadaceae
ASV7|Chryseobacterium
ASV1369|Enterobacteriaceae
ASV1338|Agrobacterium
−log10(padj)
Bacterial ASVs
(b)
Root endophytic bacteria: HG116−inoculation vs LDN−inoculation
ASV121|Actinosynnemataceae
ASV1369|Enterobacteriaceae
ASV5|Comamonadaceae
ASV546|Enterobacteriaceae
ASV579|Micromonosporaceae
ASV870|Nocardioides
−log10(padj)
Bacterial ASVs
(c)
Root endophytic bacteria: HG116−Control vs SY41−Control
ASV1112|Exiguobacterium
ASV512|Lentzea
ASV156|Aeromonas
ASV1283|Aeromonas
ASV1259|Aeromonas
ASV1188|Rubrivivax
ASV535|Arthrobacter
ASV1426|Pseudomonas
ASV1453|Lentzea
−log10(padj)
Bacterial ASVs
(d)
Root endophytic bacteria: HG116−inoculation vs SY41−inoculation
ASV1371|Enterobacter
ASV1106|Enterobacter
ASV1123|Stenotrophomonas
ASV530|[Kouleothrixaceae]
ASV922|Pseudomonas
ASV870|Nocardioides
ASV1058|Streptomycetaceae
ASV20|Arthrobacter
ASV107|Bacillus
−log10(padj)
Bacterial ASVs
(e)
Root endophytic bacteria: HG116−Control vs CS−Control
ASV656|Enterobacteriaceae
ASV1109|Erwinia
ASV1426|Pseudomonas
ASV458|Burkholderiaceae
ASV240|Variovorax
ASV56|Exiguobacterium
ASV1165|Pseudomonadaceae
ASV1238|Pseudomonas
ASV10|Pantoea
ASV1319|Comamonadaceae
ASV1453|Lentzea
ASV1112|Exiguobacterium
ASV1029|Cellulomonas
−log10(padj)
Bacterial ASVs
(f)
Root endophytic bacteria: HG116−inoculation vs CS−inoculation
ASV1371|Enterobacter
ASV1106|Enterobacter
ASV1091|Cellulomonas
ASV883|Devosia
ASV823|Comamonadaceae
ASV1481|Enterobacter
ASV20|Arthrobacter
ASV870|Nocardioides
−log10(padj)
Bacterial ASVs
(g)
Root endophytic bacteria: AK58−Control vs LDN−Control
ASV1112|Exiguobacterium
ASV56|Exiguobacterium
ASV807|Exiguobacterium
ASV1170|Exiguobacterium
ASV149|Enterobacteriaceae
ASV1371|Enterobacter
ASV1369|Enterobacteriaceae
ASV931|Erwinia
ASV512|Lentzea
ASV1391|Enterobacter
ASV1232|Micromonosporaceae
ASV451|Exiguobacterium
ASV546|Enterobacteriaceae
ASV579|Micromonosporaceae
−log10(padj)
Bacterial ASVs
(h)
Root endophytic bacteria: AK58−inoculation vs LDN−inoculation
ASV121|Actinosynnemataceae
ASV1340|Actinosynnemataceae
ASV1374|Oxalobacteraceae
ASV1383|Micromonosporaceae
ASV579|Micromonosporaceae
ASV719|Agromyces
ASV546|Enterobacteriaceae
ASV303|Methylibium
ASV938|Nocardia
ASV926|Lechevalieria
−log10(padj)
Bacterial ASVs
(i)
Root endophytic bacteria: AK58−Control vs SY41−Control
ASV149|Enterobacteriaceae
ASV1112|Exiguobacterium
ASV807|Exiguobacterium
ASV647|Enterobacteriaceae
ASV56|Exiguobacterium
ASV1259|Aeromonas
ASV1369|Enterobacteriaceae
ASV1371|Enterobacter
ASV1170|Exiguobacterium
ASV512|Lentzea
ASV931|Erwinia
ASV451|Exiguobacterium
ASV156|Aeromonas
ASV1340|Actinosynnemataceae
ASV823|Comamonadaceae
−log10(padj)
Bacterial ASVs
(j)
Root endophytic bacteria: AK58−inoculation vs SY41−inoculation
ASV1371|Enterobacter
ASV1106|Enterobacter
ASV777|Rubrivivax
ASV530|[Kouleothrixaceae]
ASV303|Methylibium
ASV719|Agromyces
ASV938|Nocardia
ASV823|Comamonadaceae
ASV107|Bacillus
−log10(padj)
Bacterial ASVs
(k)
Root endophytic bacteria: AK58−Control vs CS−Control
ASV149|Enterobacteriaceae
ASV1369|Enterobacteriaceae
ASV647|Enterobacteriaceae
ASV56|Exiguobacterium
ASV807|Exiguobacterium
ASV931|Erwinia
ASV1170|Exiguobacterium
ASV656|Enterobacteriaceae
ASV1112|Exiguobacterium
ASV1391|Enterobacter
ASV240|Variovorax
ASV451|Exiguobacterium
ASV545|Pseudomonas
−log10(padj)
Bacterial ASVs
(l)
Root endophytic bacteria: AK58−inoculation vs CS−inoculation
ASV1419|Pseudoxanthomonas
ASV1371|Enterobacter
ASV1106|Enterobacter
ASV1091|Cellulomonas
ASV823|Comamonadaceae
ASV1481|Enterobacter
ASV1065|Devosia
ASV189|Roseateles
ASV938|Nocardia
ASV1194|Sorangium
ASV802|Agrobacterium
−log10(padj)
Bacterial ASVs
Phylum
Gammaproteobacteria
Betaproteobacteria
Actinobacteria
Alphaproteobacteria
Firmicutes
Deltaproteobacteria
Bacteroidetes
Acidobacteria
Chloroflexi
Gemmatimonadetes
Verrucomicrobia
Others
DCB
Depleted
Enriched
NS

Figure S24 Comparison of the differential root endophytic bacteria Core ASVs between *F. graminearum*-tolerant and -resistant hexaploid wheats (HG116 and AK58) and other susceptible wheat varieties based on DESeq2 analysis.

(a-b) Manhattan plots showing the differences of Core ASVs in root endophytic bacteria microbiota between HG116 and LDN control samples (a), between HG116 and LDN inoculated samples (b), between HG116 and SY41 control samples (c), between HG116 and SY41 inoculated samples (d), between HG116 and CS control samples (e), between HG116 and CS inoculated samples (f), between AK58 and LDN control samples (g), between AK58 and LDN inoculated samples (h), between AK58 and SY41 control samples (i), between AK58 and SY41 inoculated samples (j), between AK58 and CS control samples (k), and between AK58 and CS inoculated samples (l). Core ASVs that were significantly enriched were depicted as vertical triangles, that were significantly depleted were depicted as inverted triangles, otherwise depicted as circles. The red and blue dashed line corresponded to the FDR-corrected pvalue of 0.01 and 0.05, respectively. The color of each point represented the phylum-level taxonomic affiliation of the Core ASVs, and the size corresponded to the baseMean of the Core ASVs.

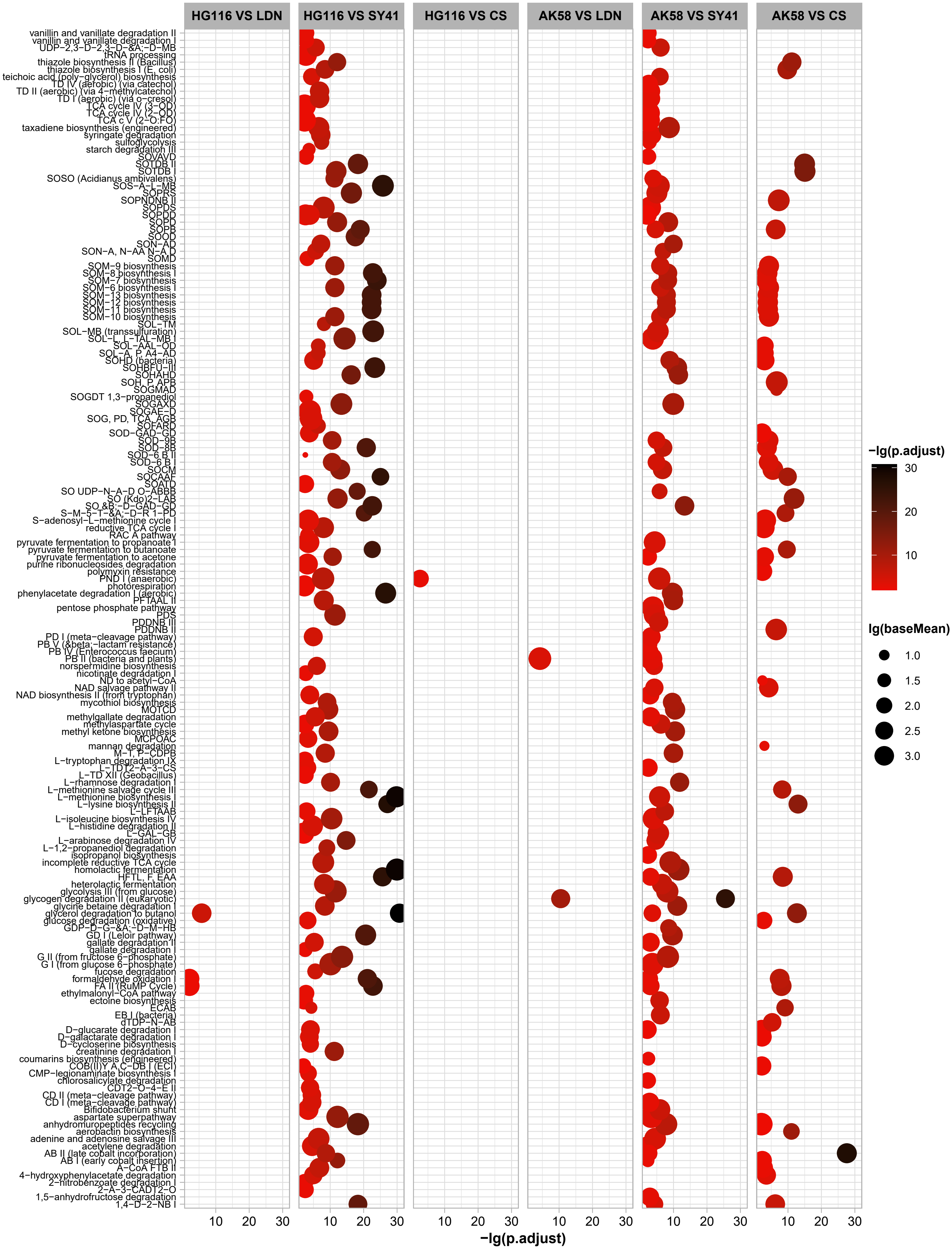

Figure S25 Bubble plot of significantly enriched functional profiles inferred by PICRUSt2 in rhizosphere bacterial microbiota of *F. graminearum*-tolerant and -resistant hexaploid wheats (HG116 and AK58) compared to other susceptible wheat varieties following PH-1 infection. Each point represented a significantly enriched MetaCyc pathway prediction in resistant allohexaploid wheats (HG116, AK58) relative to other susceptible wheat varieties based on DESeq2 analysis. The size of bubble points showing pathway abundances, the color of bubble points showing enrichment significance (*PFDR* < 0.01). The full names of the abbreviations for the descriptions of MetaCyc pathways are detailed in Table S8.

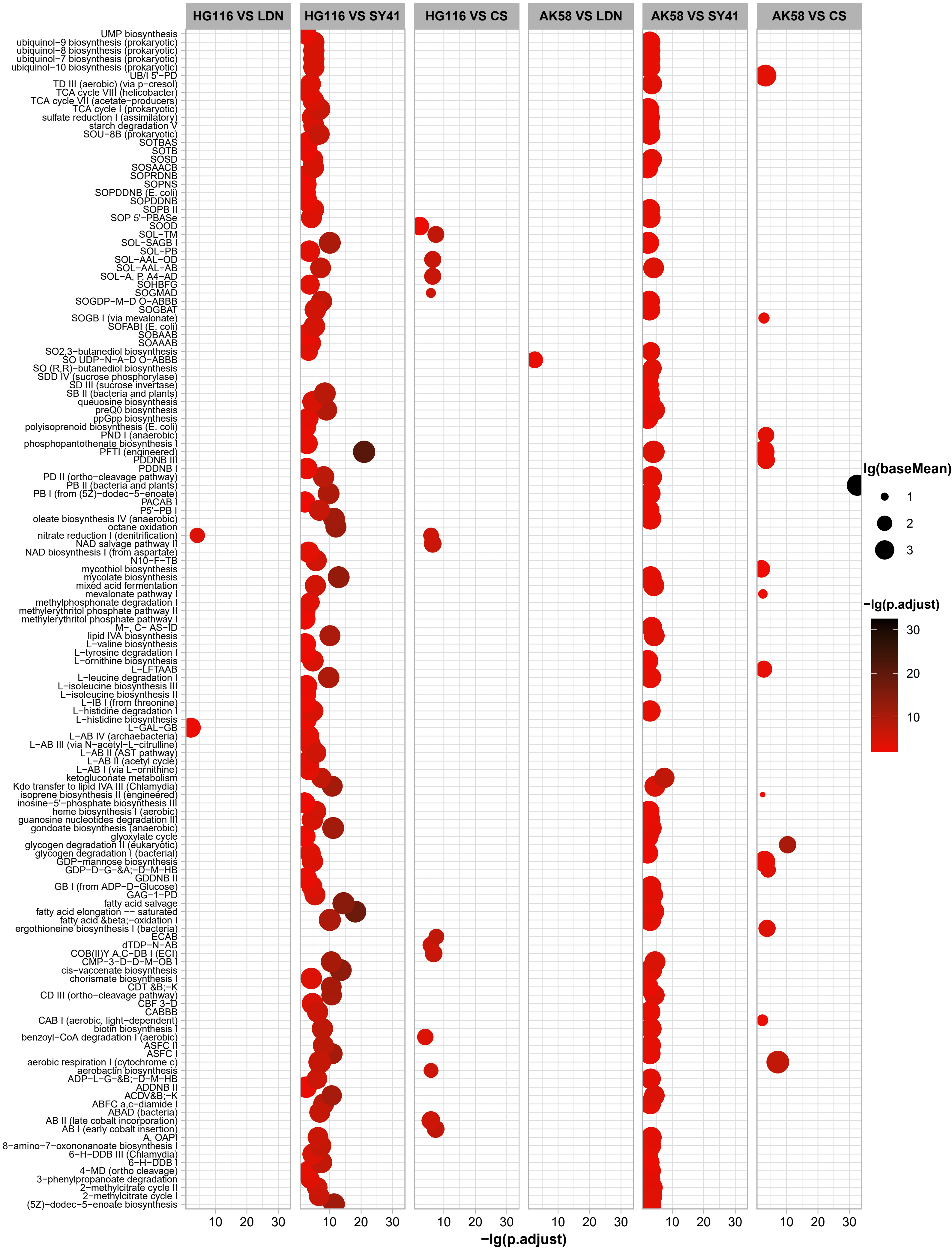

Figure S26 Bubble plot of significantly depleted functional profiles inferred by PICRUSt2 in rhizosphere bacterial microbiota of *F. graminearum*-tolerant and -resistant hexaploid wheats (HG116 and AK58) compared to other susceptible wheat varieties following PH-1 infection. Each point represents a significantly depleted MetaCyc pathway prediction in disease-resistant allohexaploid wheat (HG116, AK58) relative to other disease-susceptible wheat varieties based on DESeq2 analysis. The size of bubble points showing pathway abundances, the color of bubble points showing enrichment significance (*PFDR* < 0.01). The full names of the abbreviations for the descriptions of MetaCyc pathways are detailed in Table S8.

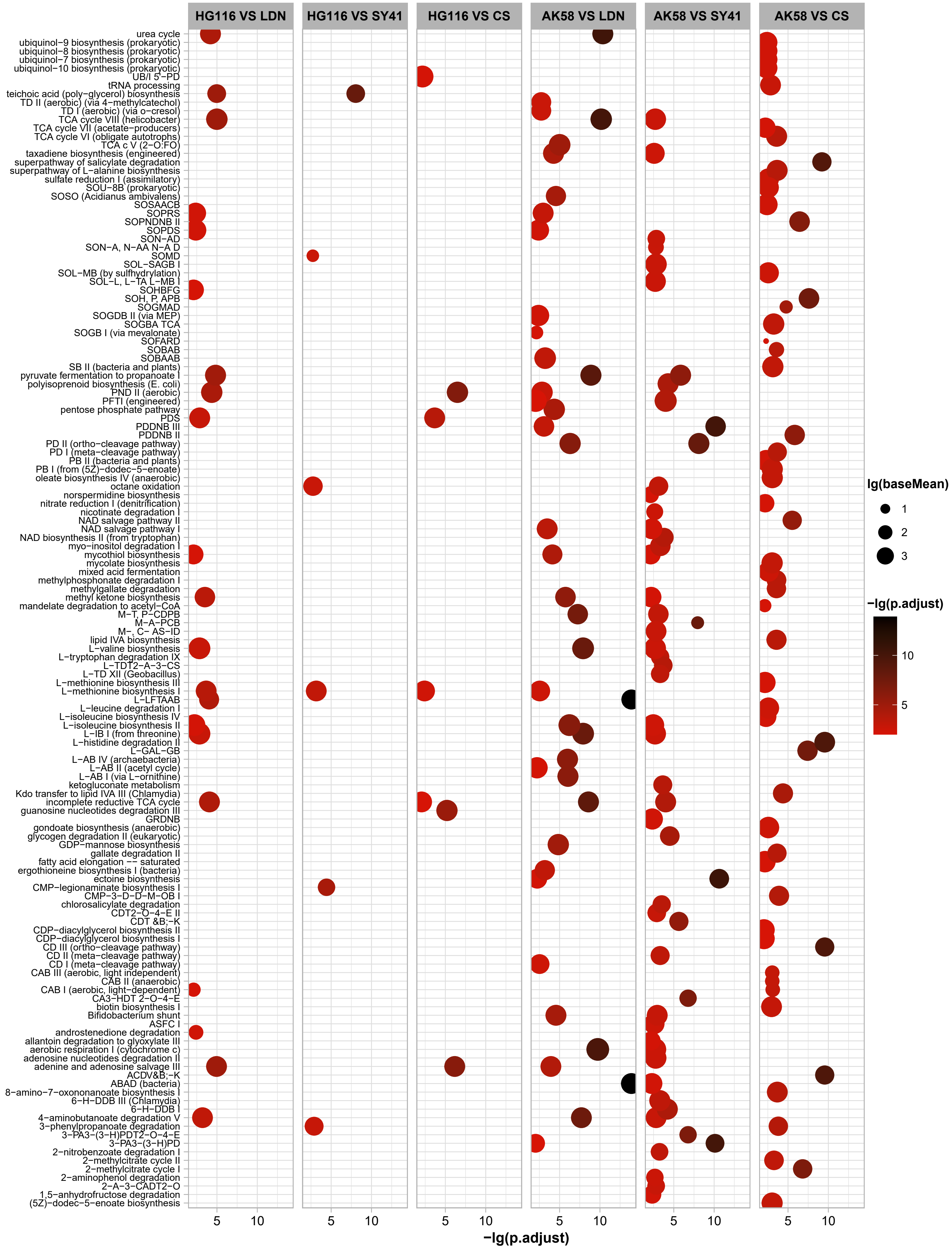

Figure S27 Bubble plot of significantly enriched functional profiles inferred by PICRUSt2 in root endophytic bacterial microbiota of *F. graminearum*-tolerant and -resistant hexaploid wheats (HG116 and AK58) compared to other susceptible wheat varieties following PH-1 infection. Each point represents a significantly enriched MetaCyc pathway prediction in disease-resistant allohexaploid wheat (HG116, AK58) relative to other disease-susceptible wheat varieties based on DESeq2 analysis. The size of bubble points showing pathway abundances, the color of bubble points showing enrichment significance (*PFDR* < 0.01). The full names of the abbreviations for the descriptions of MetaCyc pathways are detailed in Table S8.

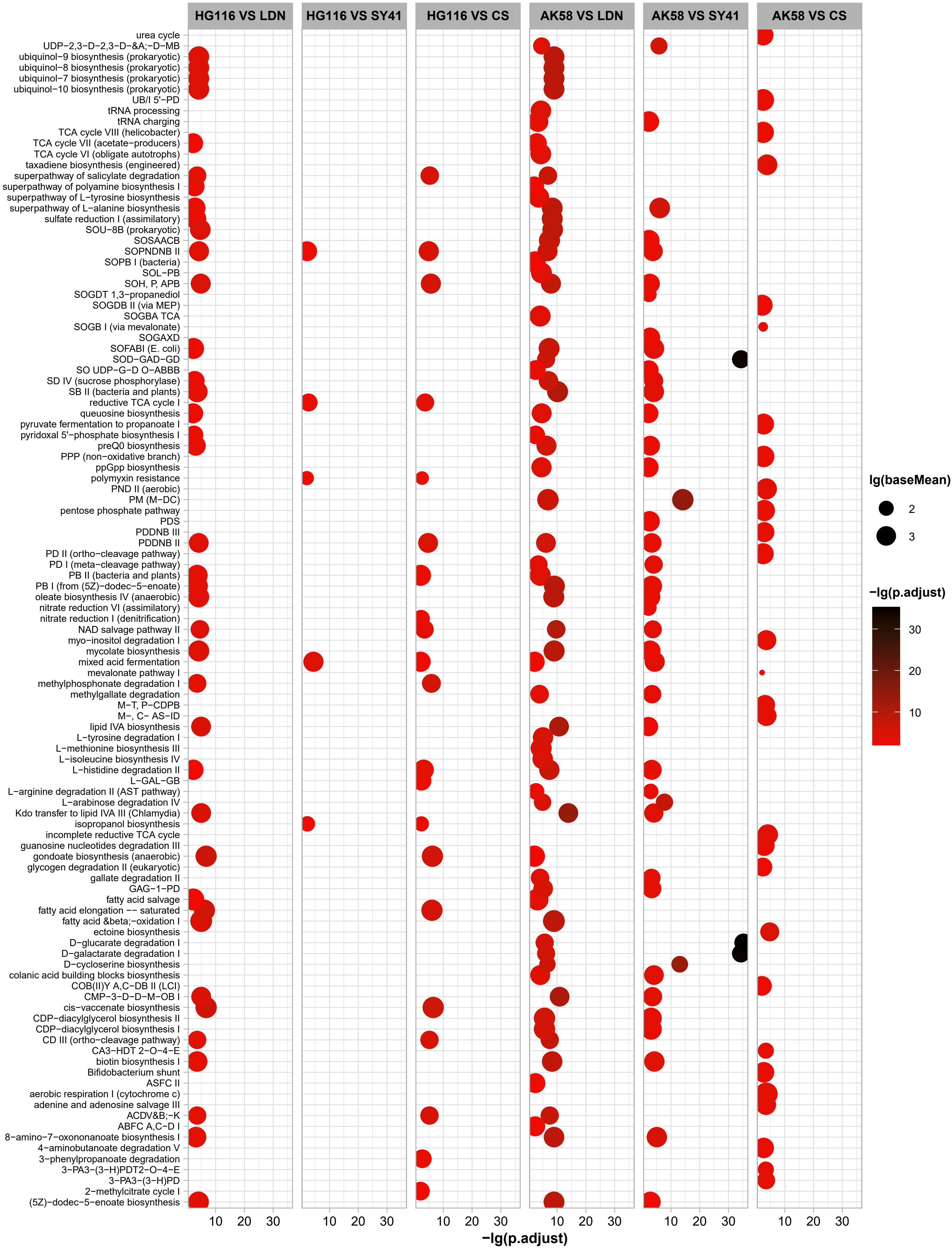

Figure S28 Bubble plot of significantly depleted functional profiles inferred by PICRUSt2 in root endophytic bacterial microbiota of *F. graminearum*-tolerant and -resistant hexaploid wheats (HG116 and AK58) compared to other susceptible wheat varieties following PH-1 infection. Each point represents a significantly depleted MetaCyc pathway prediction in disease-resistant allohexaploid wheat (HG116, AK58) relative to other disease-susceptible wheat varieties based on DESeq2 analysis. The size of bubble points showing pathway abundances, the color of bubble points showing enrichment significance (*PFDR* < 0.01). The full names of the abbreviations for the descriptions of MetaCyc pathways are detailed in Table S8.

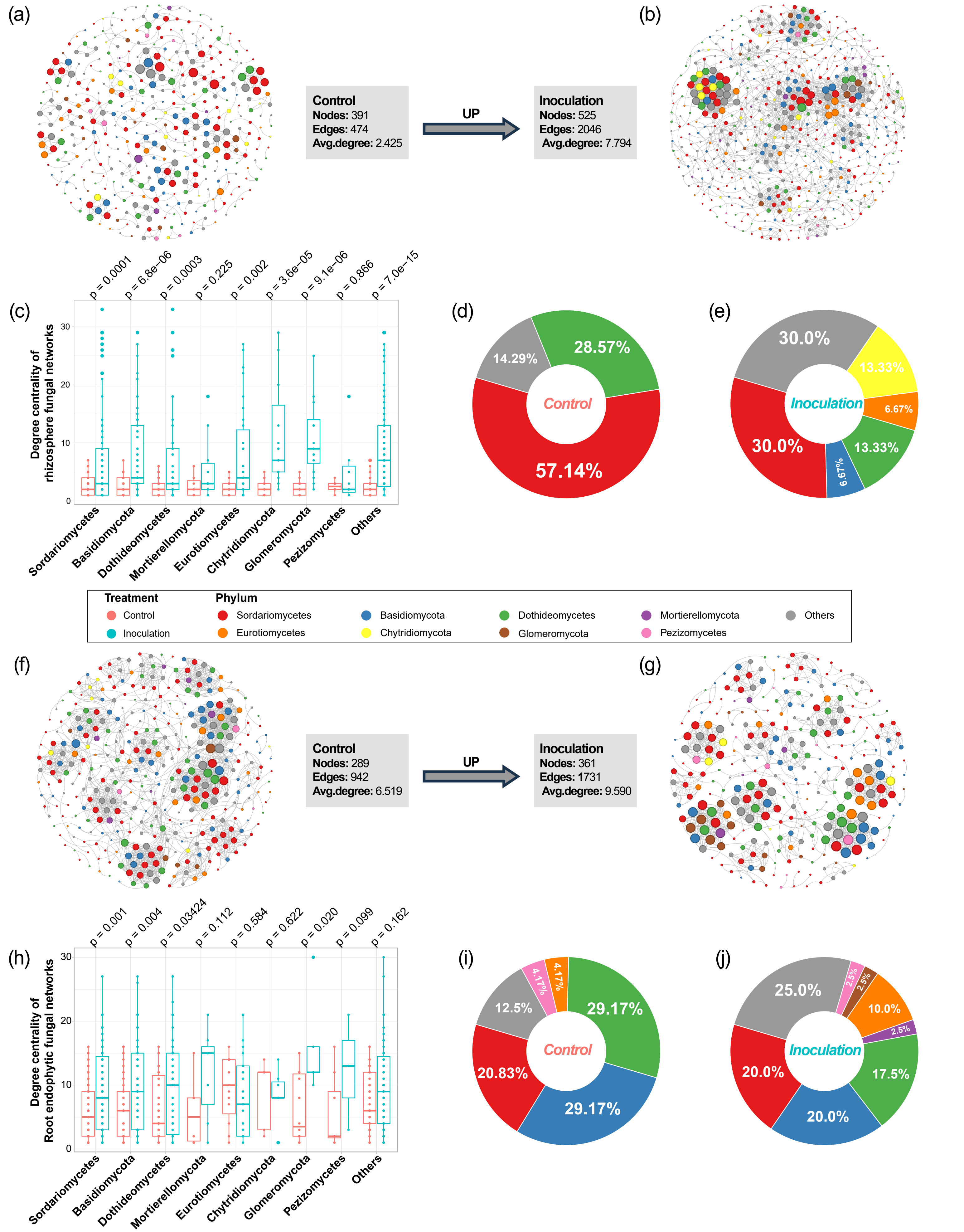

Figure S29 *F. graminearum* infection altered wheat root-associated fungal microbial networks.
(a,b, f and g) The rhizosphere (a and b) and root endophytic (f and g) fungal co-abundance networks of control (a, f) and inoculated (b, g) groups of all wheat varieties on the whole. A connection represented a significant correlation (Spearman's correlation test, $R > 0.6$, $PFDR < 0.05$). Nodes belonging to members of Sordariomycetes, Basidiomycota, Dothideomycetes, Mortierellomycota, Eurotiomycetes, Chytridiomycota, Glomeromycota and Pezizomycetes were colored. The size of each node was proportional to the degree of each node. Avg.degree was the average of the degrees across all nodes in the rhizosphere fungal co-abundance networks.
(c, h) Comparing the distribution of degree centrality of fungal phyla in rhizosphere (c) and root endophytic (h) fungal co-abundance networks between control and inoculated groups of all wheat varieties. Statistical significance was determined via Wilcoxon test and with FDR correction (α = 0.05). (d, e, i and j) Pie graph showed the proportion of fungal phyla appearing in hub nodes of the rhizosphere (d and e) and root endophytic (i and j) fungal co-abundance networks of control (d, i) and inoculated (e, j) groups. The color of each slice corresponded to the color of the node in the rhizosphere fungal co-abundance networks.

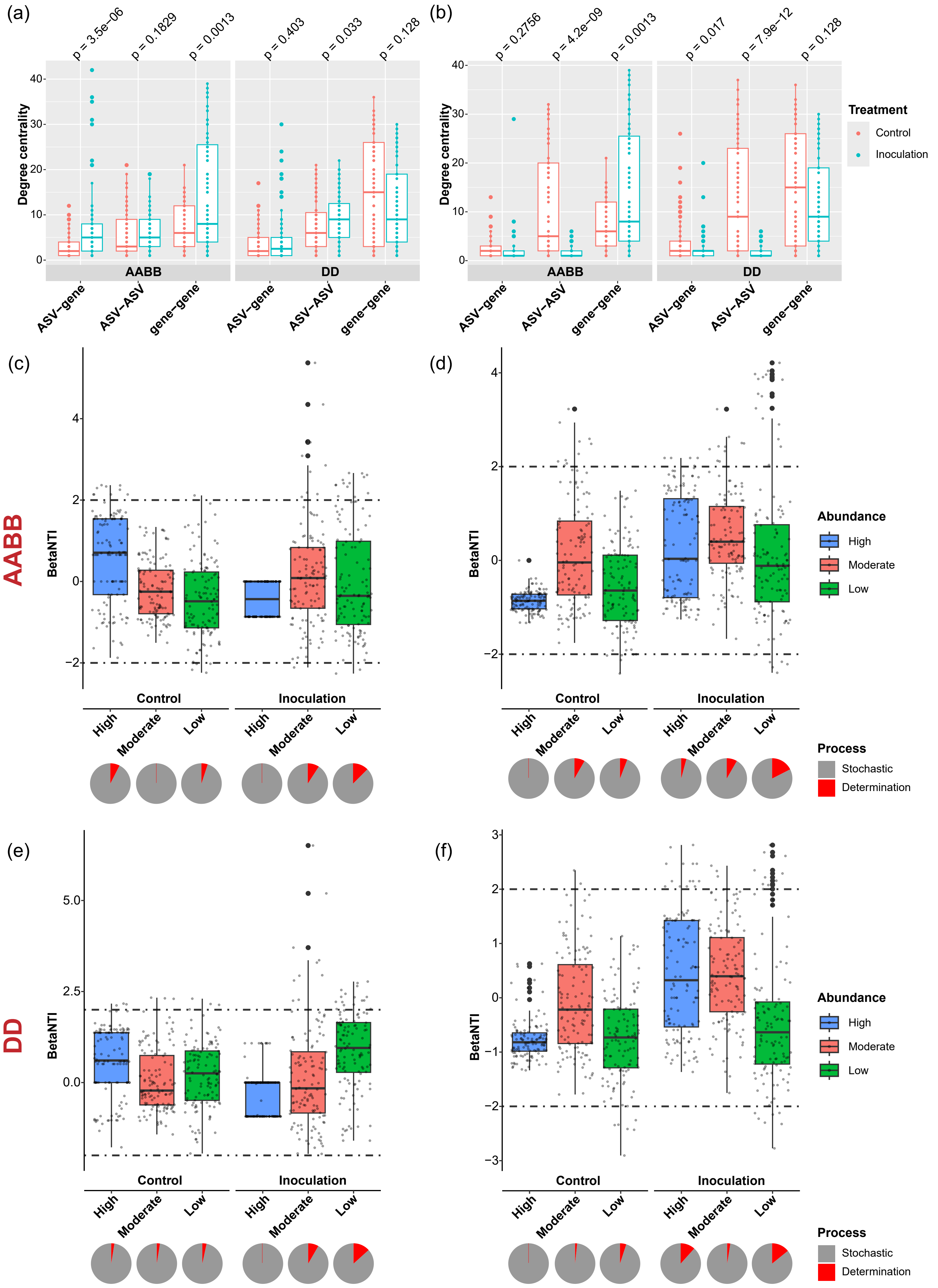
(a)
(b)
p = 3.5e−06
p = 0.1829
p = 0.0013
p = 0.403
p = 0.033
p = 0.128
p = 0.2756
p = 4.2e−09
p = 0.0013
p = 0.017
p = 7.9e−12
p = 0.128
Degree centrality
AABB
DD
ASV−gene
ASV−ASV
gene−gene
Treatment
Control
Inoculation
(c)
(d)
(e)
(f)
AABB
DD
BetaNTI
Control
Inoculation
High
Moderate
Low
Abundance
Process
Stochastic
Determination

Figure S30 The distribution of degree centrality of all subnetworks and betaNTI of bacterial microbiota in the correlation networks between the root-associated bacterial microbiome and root transcriptome.
(a and b) Comparing the distribution of degree centrality of all subnetworks in the correlation networks between the wheat rhizosphere bacterial microbiome and root transcriptome (a), and between the wheat root endophytic bacterial microbiome and root transcriptome (b), between control and inoculated groups. AABB represented wheats with AABB subgenomes. DD represented wheats with DD subgenome. Statistical significance was determined via Wilcoxon test and with FDR correction ($\alpha = 0.05$). (c-f) Comparing the distribution of beta Nearest Taxon Index (betaNTI) among high, moderate and low-abundance bacterial microbiota in the correlation networks between the rhizosphere (c and e) bacterial microbiome and root transcriptome, and between root endophytic (d and f) bacterial microbiome and root transcriptome, between control and inoculated groups, (c, d) of wheats with AABB subgenomes, (e, f) of wheats with DD subgenome. Positive (negative) betaNTI values indicated greater (lesser) than expected turnover in phylogenetic composition. $|BetaNTI| < 2$ indicated the community assembly was dominated by stochastic processes. $|BetaNTI| > 2$ indicated the community assembly was dominated by deterministic processes. Pie graph showed the contribution rates of stochastic and deterministic processes in the community assembly. Stochastic and deterministic processes were represented by the grey and red respectively.

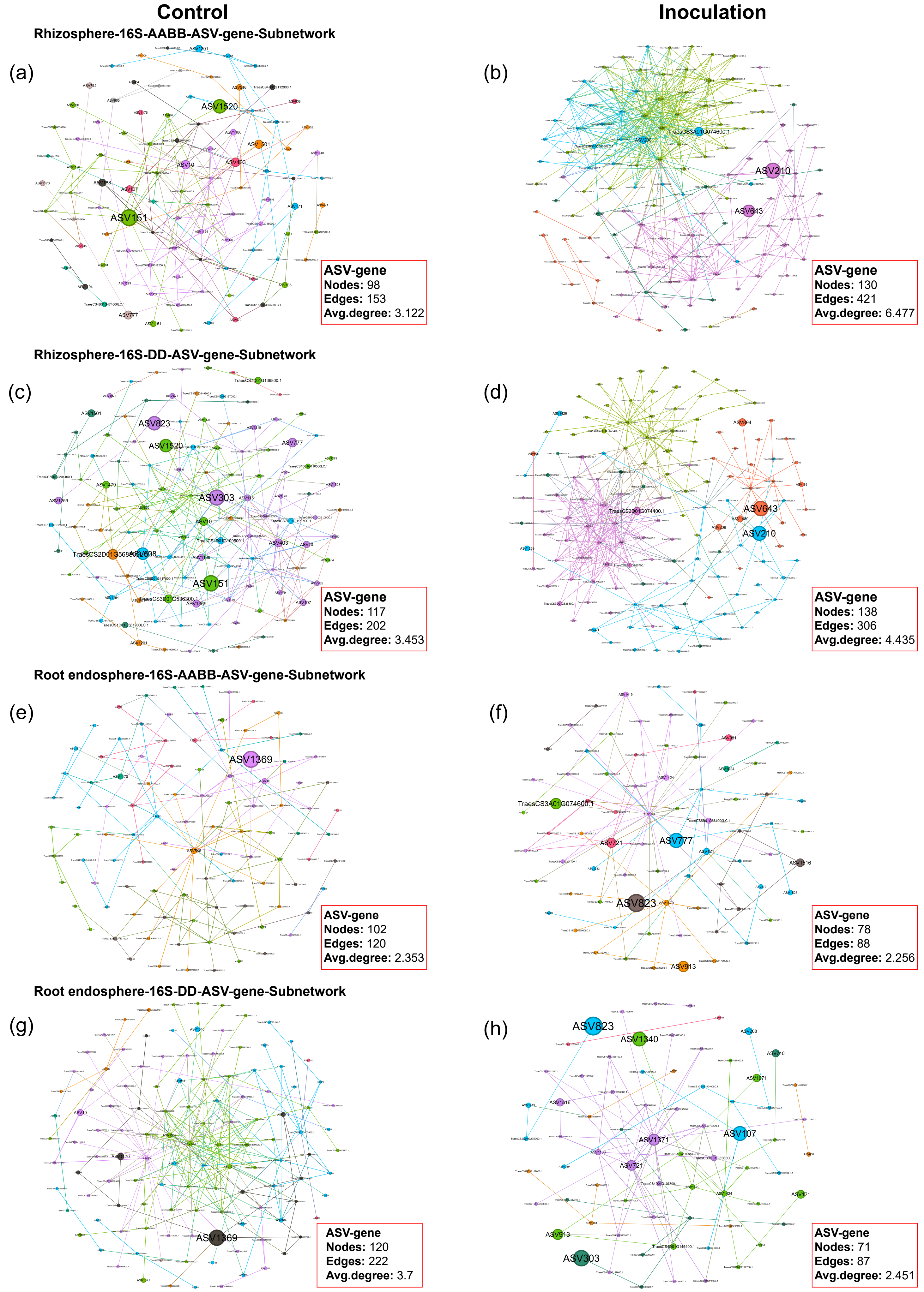

Figure S31 The comparison of ASV-gene subnetworks in the correlation networks between the root-associated bacterial microbiome and root transcriptome between control and inoculated groups.

(a-d) ASV-gene subnetworks in the correlation networks between the rhizosphere bacterial microbiomes and root transcriptome, under control (a, c) and inoculation (b, d), (a and b) of wheats with AABB subgenomes, (c and d) of wheats with DD subgenome. (e-h) ASV-gene subnetworks in the correlation networks between the root endophytic bacterial microbiomes and root transcriptome, under control (e, g) and inoculation (f, h), (e and f) of wheats with AABB subgenomes, (g and h) of wheats with DD subgenome. The size of each nodes indicated the relative abundance of each bacterial Core ASV, or the expression level of each root gene. Nodes with the same color suggested stronger correlations among them.

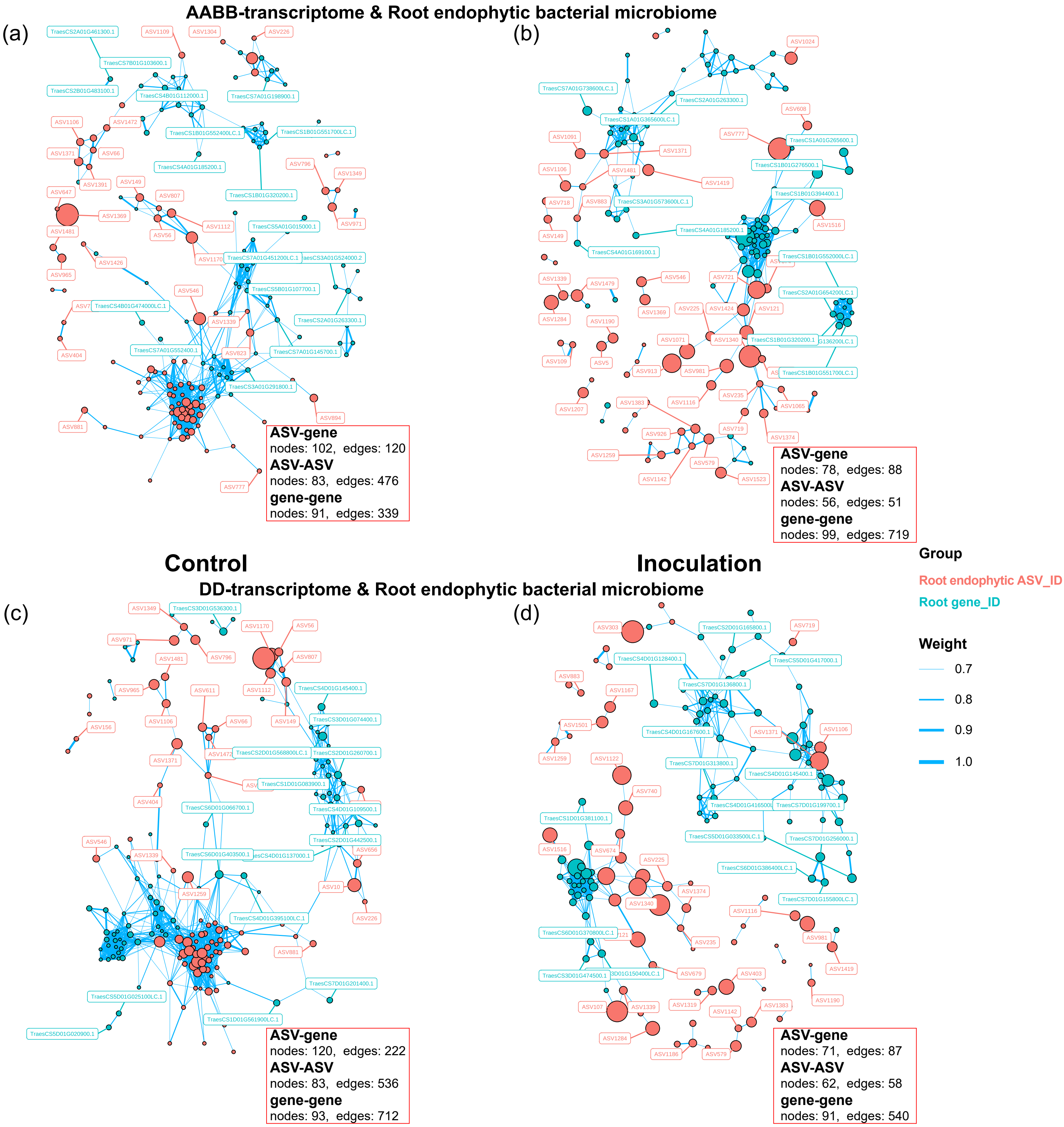

Figure S32 The correlation networks between the root endophytic bacterial microbiome and root transcriptome.
(a-d) The correlation networks between the root endophytic bacterial microbiome and root transcriptome of wheats with AABB (a and b) subgenomes, and with DD subgenome (c and d), under control (a, c) and inoculation (b, d). The root endophytic bacterial Core ASVs were indicated in salmon color, and wheat root genes were indicated in darkturquoise. Weight indicated the significant correlation between network nodes (Spearman's correlation test, R >0.07, P < 0.01). The size of each nodes indicated the relative abundance of each root endophytic bacterial Core ASV, or the expression level of each root gene. ASV-gene represented the correlation subnetwork between wheat root endophytic bacterial microbiota and genes; ASV-ASV represented the correlation subnetwork among wheat root endophytic bacteria; gene-gene represented the correlation subnetwork among wheat root genes.

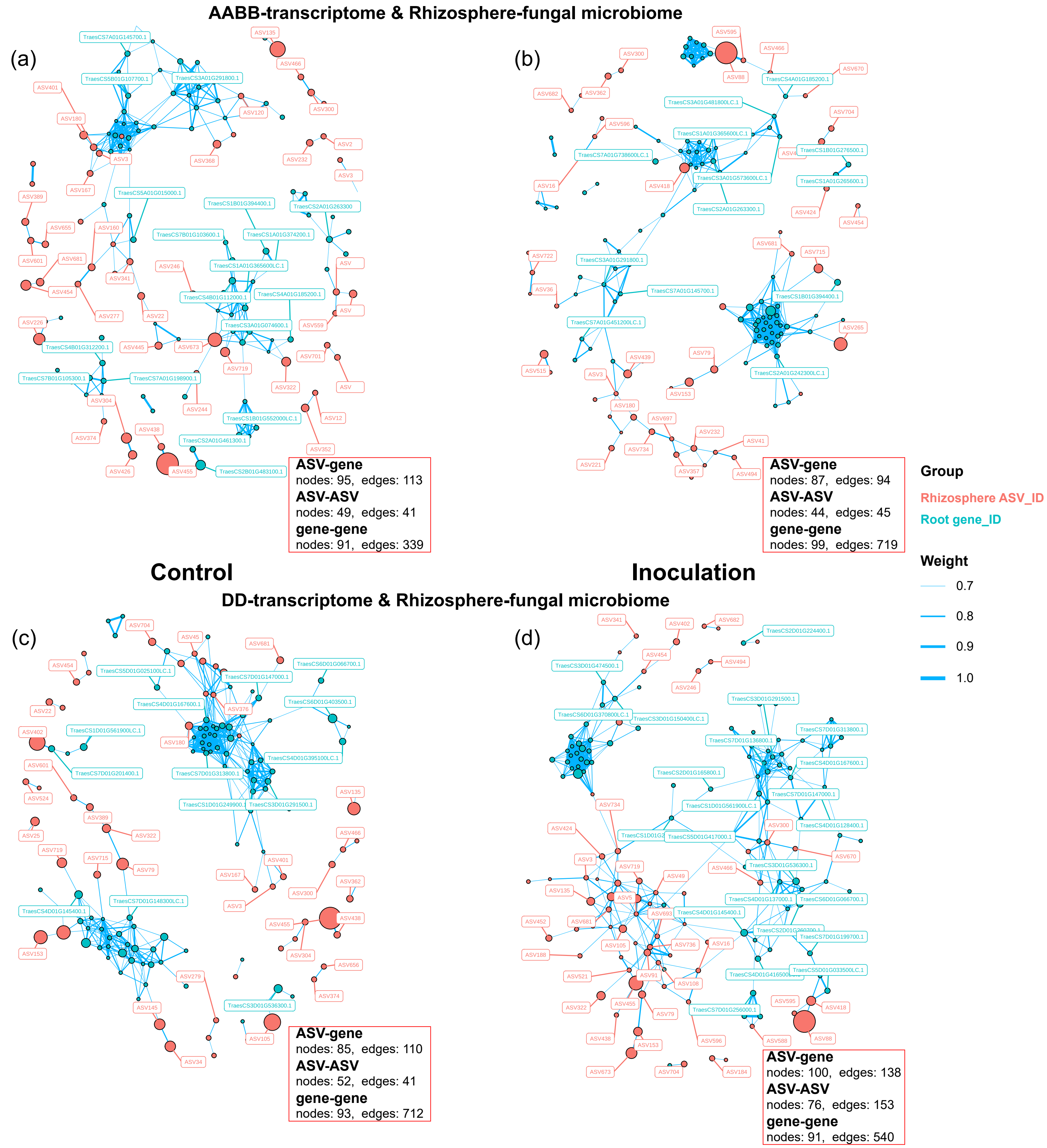

Figure S33 The correlation networks between the rhizosphere fungal microbiome and root transcriptome.
(a-d) The correlation networks between the rhizosphere fungal microbiome and root transcriptome of wheats with AABB (a and b) subgenomes, and with DD subgenome (c and d), under control (a, c) and inoculation (b, d). The rhizosphere fungal Core ASVs were indicated in salmon color, and wheat root genes were indicated in darkturquoise. Weight indicated the significant correlation between network nodes (Spearman's correlation test, R >0.07, P < 0.01). The size of each nodes indicated the relative abundance of each rhizosphere fungal Core ASV, and the expression levels of each root gene. ASV-gene represented the correlation subnetwork between wheat rhizosphere fungal microbiota and genes; ASV-ASV represented the correlation subnetwork among wheat rhizosphere fungi; gene-gene represented the correlation subnetwork among wheat root genes.

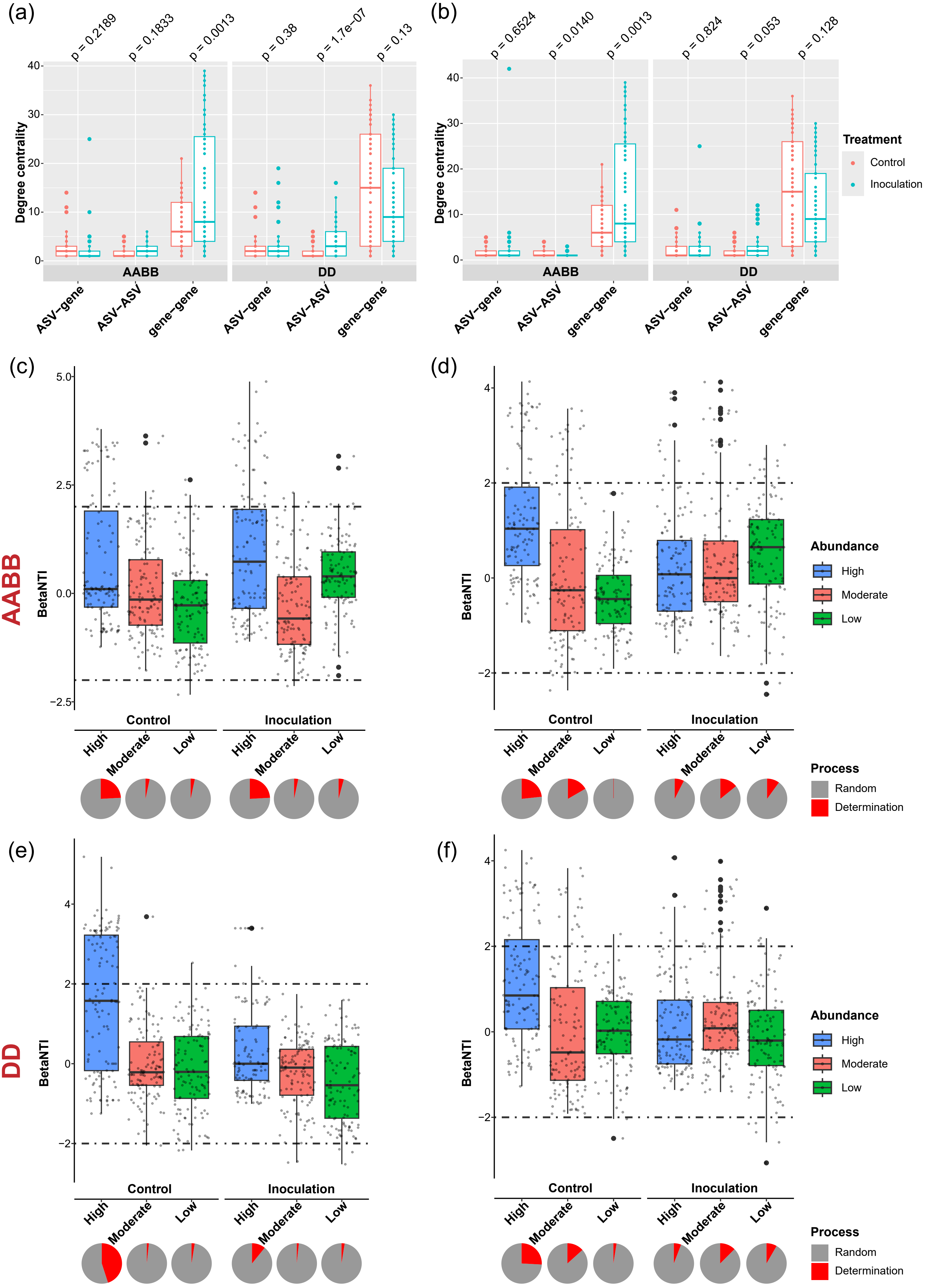

(a)
p = 0.2189
p = 0.1833
p = 0.0013
p = 0.38
p = 1.7e−07
p = 0.13
Degree centrality
AABB
DD
ASV−gene
ASV−ASV
gene−gene
(b)
p = 0.6524
p = 0.0140
p = 0.0013
p = 0.824
p = 0.053
p = 0.128
Treatment
Control
Inoculation
(c)
(d)
(e)
(f)
AABB
DD
BetaNTI
Control
Inoculation
High
Moderate
Low
Abundance
Process
Random
Determination

Figure S34 The distribution of degree centrality of all subnetworks and betaNTI of fungal microbiota in the correlation networks between the root-associated fungal microbiome and root transcriptome.
(a and b) Comparing the distribution of degree centrality of all subnetworks in the correlation networks between the wheat rhizosphere fungal microbiome and root transcriptome (a), and between the wheat root endophytic fungal microbiome and root transcriptome (b), between control and inoculated groups. AABB represented wheats with AABB subgenomes. DD represented wheats with DD subgenome. Statistical significance was determined via Wilcoxon test and with FDR correction ($\alpha = 0.05$). (c-f) Comparing the distribution of beta Nearest Taxon Index (betaNTI) among high, moderate and low-abundance fungal microbiota in the correlation networks between the rhizosphere (c and e) fungal microbiome and root transcriptome, and between root endophytic (d and f) fungal microbiomes and root transcriptome, between control and inoculated groups, (c, d) of wheats with AABB subgenomes, (e, f) of wheats with DD subgenome. Positive (negative) betaNTI values indicated greater (lesser) than expected turnover in phylogenetic composition. $|BetaNTI| < 2$ indicated the community assembly was dominated by stochastic processes. $|BetaNTI| > 2$ indicated the community assembly was dominated by deterministic processes. Pie graph showed the contribution rates of stochastic and deterministic processes in the community assembly. Stochastic and deterministic processes were represented by the grey and red respectively.

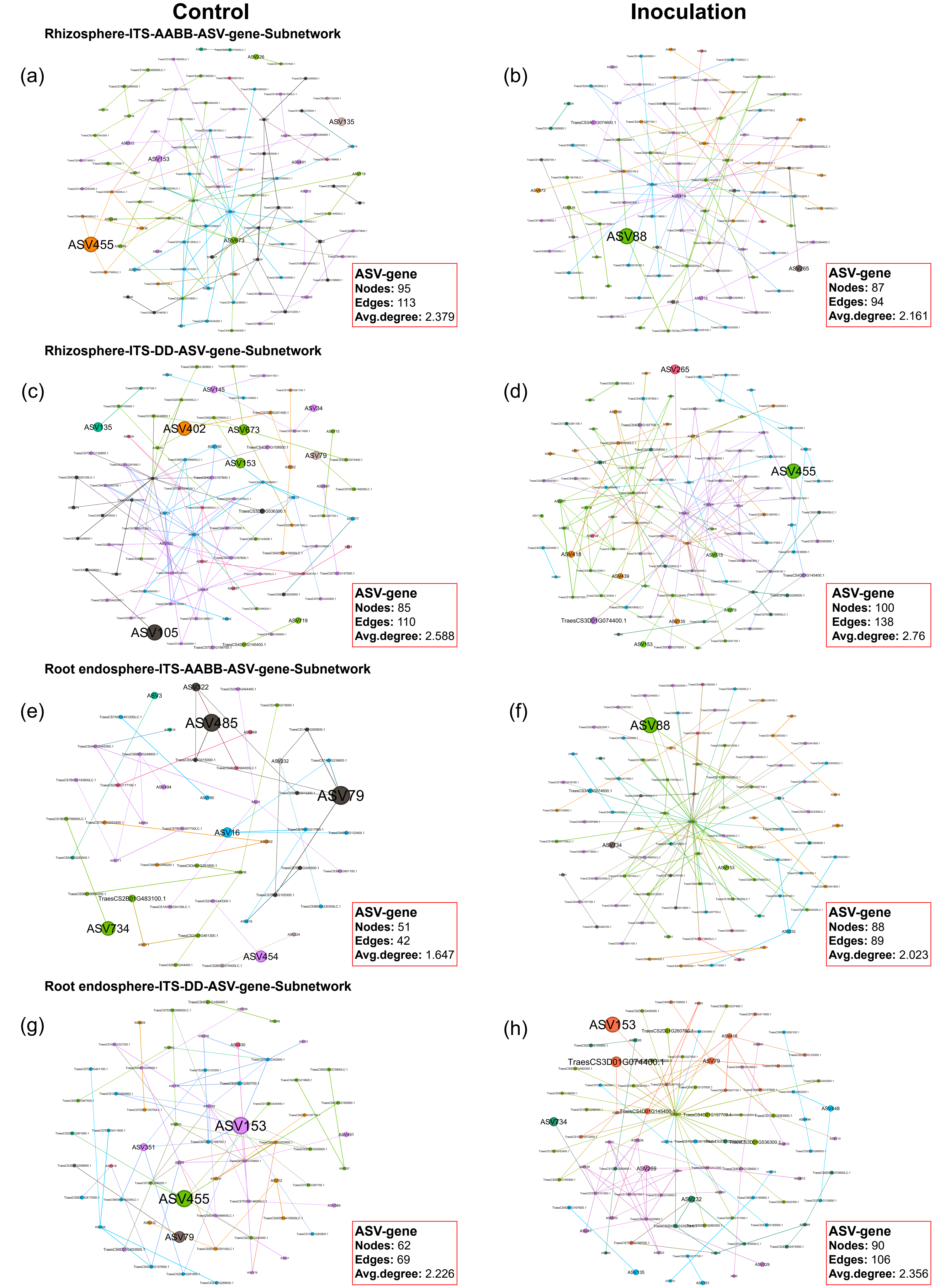

Figure S35 The comparison of ASV-gene subnetworks in the correlation networks between the root-associated fungal microbiome and root transcriptome between control and inoculated groups

(a-d) ASV-gene subnetworks in the correlation networks between the rhizosphere fungal microbiomes and root transcriptome, under control (a, c) and inoculation (b, d), (a and b) of wheats with AABB subgenomes, (c and d) of wheats with DD subgenome. (e-h) ASV-gene subnetworks in the correlation networks between the root endophytic fungal microbiome and root transcriptome, under control (e, g) and inoculation (f, h), (e and f) of wheats with AABB subgenomes, (g and h) of wheats with DD subgenome. The size of each nodes indicated the relative abundance of each fungal Core ASV, or the expression levels of each root gene. Nodes with the same color suggested stronger correlations among them.

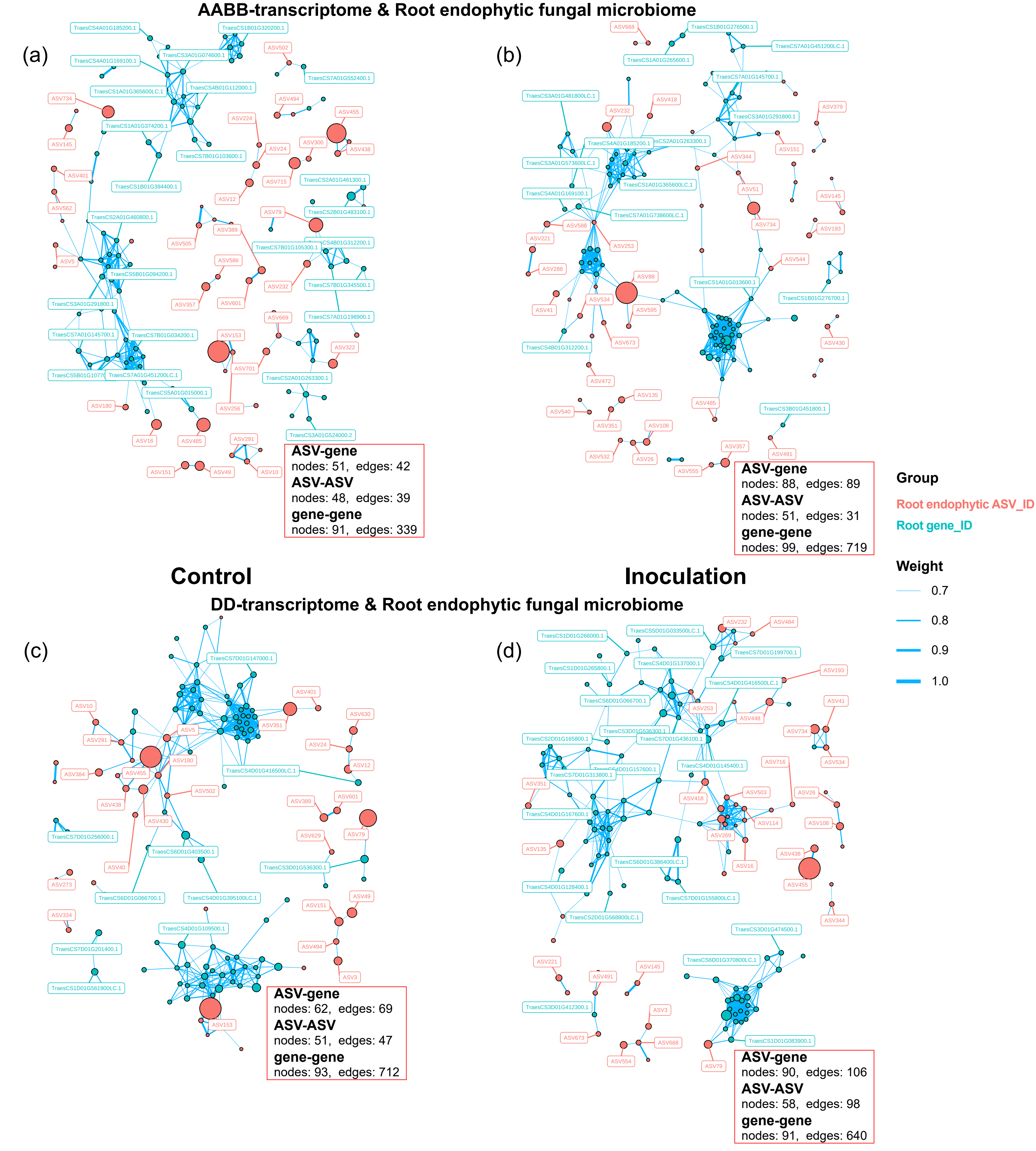

Figure S36 The correlation networks between the root endophytic fungal microbiome and root transcriptome.
(a-d) The correlation networks between the root endophytic fungal microbiome and root transcriptome of wheats with AABB (a and b) subgenomes, and with DD subgenome (c and d), under control (a, c) and inoculation (b, d). The root endophytic fungal Core ASVs were indicated in salmon color, and wheat root genes were indicated in darkturquoise. Weight indicated the significant correlation between network nodes (Spearman's correlation test, $R > 0.07$, $P < 0.01$). The size of each nodes indicated the relative abundance of each root endophytic fungal Core ASV, and the expression levels of each root gene. ASV-gene represented the correlation subnetwork between wheat root endophytic fungal microbiota and genes; ASV-ASV represented the correlation subnetwork among wheat root endophytic fungi; gene-gene represented the correlation subnetwork among wheat root genes.

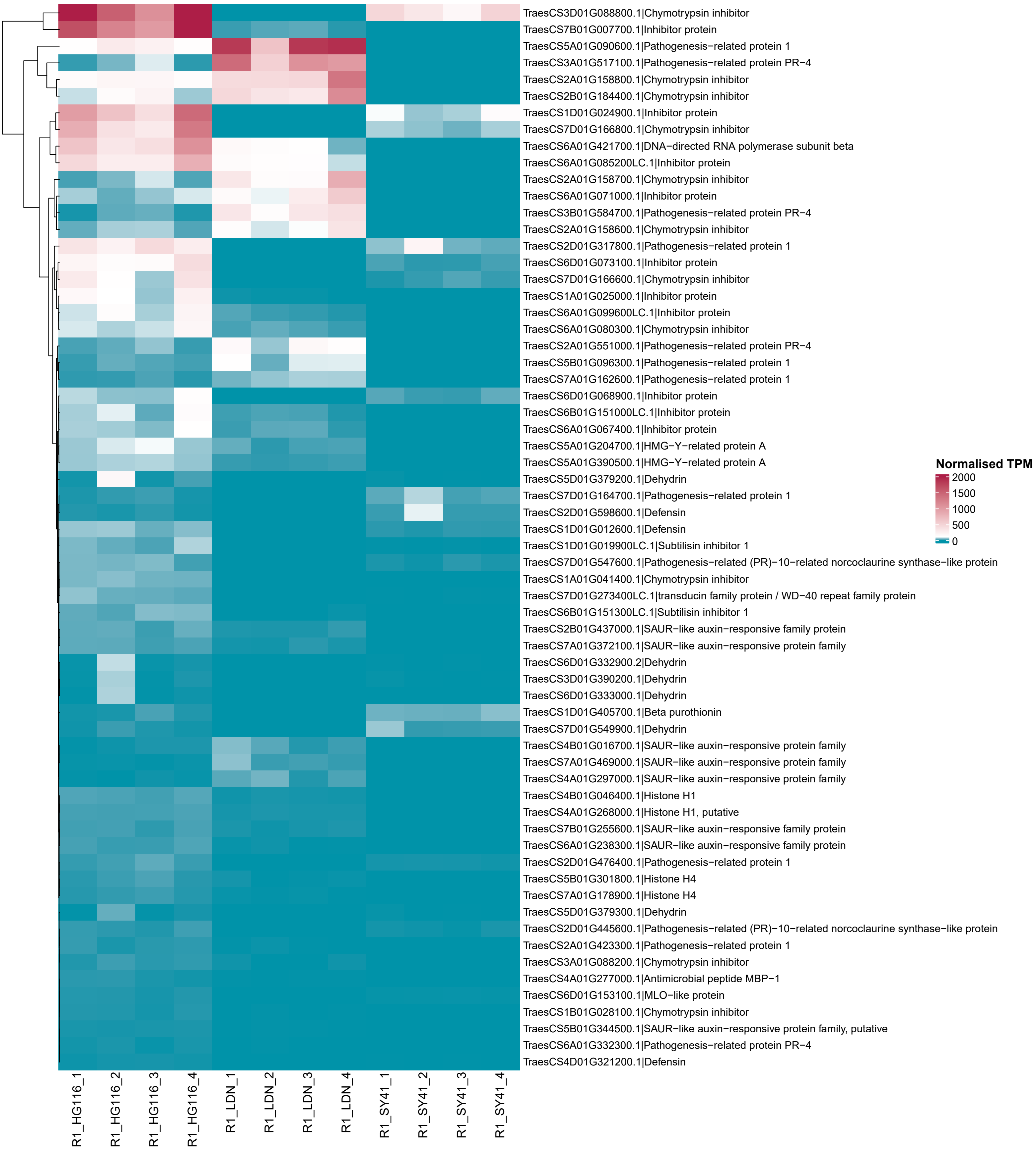

Figure S37 Hierarchical clustering analysis heatmap to compare the expression levels of differentially expressed genes (DEGs with $|\log_2 FC| > 2$ and $q < 0.05$) enriched in stress and defences related pathways between HG116 and LDN, and SY41 before PH-1 inoculation. The stress and defences related pathways included response to wounding, response to biotic stimulus, defence response to fungus, defence response to bacterium, response to auxin, nucleosome assembly, response to water and response (Figure 1e-f).

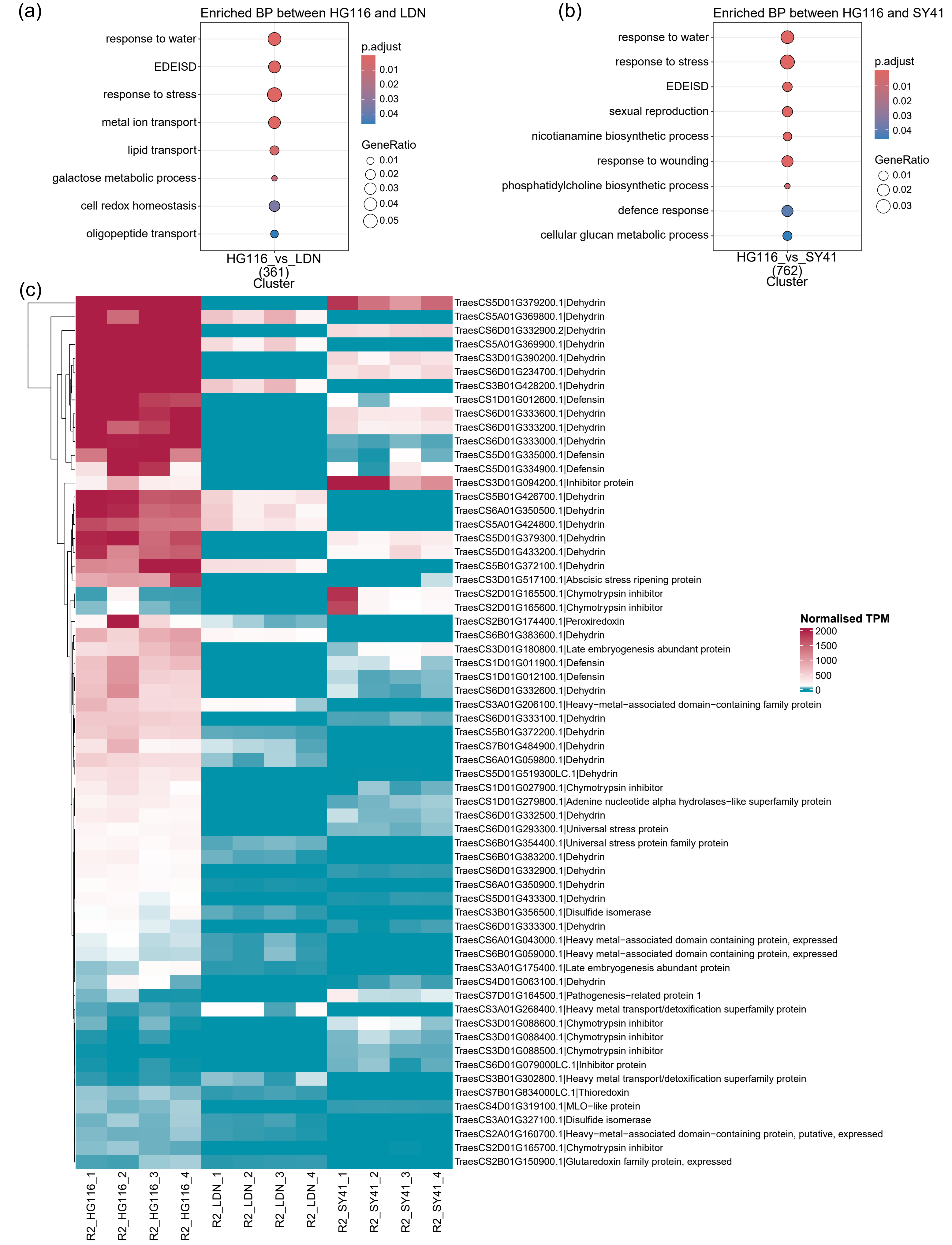

Figure S38 Gene ontology (GO) annotations and Hierarchical clustering analysis of differentially expressed genes (DEGs with $|\log_2 FC| > 2$ and $q < 0.05$) between HG116 and LDN (a), and SY41(b) after PH-1 inoculation. The GeneRatio and BH-adjusted enrichment pvalue of the biological processes were indicated using the size and color of the bubble points, respectively. The color of dots indicated high or low enrichment for a specific GO category. The size of dots displayed the overlap between the input gene lists with the collection of gene sets. EDEISD: embryo development ending in seed dormancy. (c) Hierarchical clustering analysis heatmap for comparing the expression levels of all differentially expressed genesenriched in stress and defences related pathways between HG116 and LDN, and SY41 after PH-1 inoculation. The stress and defences related pathways included response to water, response to stress, metal ion transport, cell redox homeostasis, response to wounding and defence response.
